# Correction: Human demographic history has amplified the effects of background selection across the genome

**DOI:** 10.1371/journal.pgen.1007898

**Published:** 2019-01-02

**Authors:** Raul Torres, Zachary A. Szpiech, Ryan D. Hernandez

There is an error in the second paragraph under the subheading “Simulations” of the Materials and methods section. Specifically, in the fourth and sixth sentences, it states that the two distribution of fitness effects used in the simulations were drawn from two gamma distributions with parameters (β = 0.0415, α = 0.00515625) and (β = 0.184, α = 0.00040244), with their unscaled values being (β = 0.0415, α = 80.11) and (β = 0.184, α = 6.25), respectively. This is incorrect and the α and β parameters are switched for each pair of parameters. The correct ones are (α = 0.0415, β = 0.00515625) and (α = 0.184, β = 0.00040244), with their unscaled values being (α = 0.0415, β = 80.11) and (α = 0.184, β = 6.25), respectively. This error has no quantitative or qualitative impact on the results or conclusions of the study.
