# Supplementary material for: Human demographic history has amplified the effects of background selection across the genome
Source: PLoS Genet. 2018 Jun 18;14(6):e1007387. doi: 10.1371/journal.pgen.1007387 (PMC6056204; doi:10.1371/journal.pgen.1007387)
Supplement: S1 File — (GZ) [file pgen.1007387.s001.tar.gz › SFS_CODE_torres_et_al_2018/doc/SFS_CODE_doc.pdf]

Selection on **F**inite **S**ites under **CO**mplex **D**emographic **E**vents (SFS_CODE)

Ryan D. Hernandez

Draft date: September 10, 2015

Preface

This is the user's guide to SFS_CODE. It outlines how to compile and use the program, and many (but not all) of the details regarding specific algorithms and underlying data structures implemented in the C source code. Table 6 on page 56 outlines every option implemented in SFS_CODE, with a linked page reference indicating where each option was described in the text.

Please note that this program is free software: you can redistribute it and/or modify it under the terms of the GNU General Public License as published by the Free Software Foundation, either version 3 of the License, or (at your option) any later version. This program is distributed in the hope that it will be useful, but WITHOUT ANY WARRANTY; without even the implied warranty of MERCHANTABILITY or FITNESS FOR A PARTICULAR PURPOSE. See the GNU General Public License for more details. A copy of the GNU General Public License should be in the folder doc that was distributed with this program. If not, see http://www.gnu.org/licenses/.

Contents

1 Overview **4**

2 Citing this program **5**

3 Getting Started **5**

3.1 Obtaining the Program 5

3.2 Compiling the Program 5

3.3 Usage: Arguments at the Command Line 5

4 Running SFS_CODE **6**

4.1 Population Expansions and Bottlenecks 11

4.2 Distribution of Selective Effects 14

4.3 Multiple Populations 19

4.4 Mutation Models 23

4.5 Recombination: Crossing-over and Gene Conversion 27

4.5.1 Recombination Hotspots 31

4.6 Insertions and Deletions 31

4.7 Inversions 32

4.8 Selfing and Generation-Effects 32

4.9 Changing Parameters Over Time 33

5 The Simulated Population Size *Sometimes* Does Not Matter **34**

6 Sampling From an Extinct Lineage **35**

7 Using SFS_CODE on a Cluster **37**

7.1 Your own Cluster 37

7.2 Using SFS_CODE on the CBSU Web Cluster 38

8 Understanding the Output **39**

8.1 No indels or inversions 39

8.2 With Indels and/or Inversions 42

8.3 Population-wide Allele Frequencies 43

8.4 Tracking the Trajectory of an Allele 43

8.5 Report the True Genetic Ancestry of Each Site 45

8.6 Output in VCF format 45

9 Using convertSFS_CODE to Generate Useful Data **46**

10 A Complicated Example for Converting Between `ms` and `SFS_CODE` Command-lines **51**

11 Examples **53**

11.1 Recurrent Hitchhiking 53

12 Default Parameter Values **54**

13 Summary of Options and Arguments **54**

1 Overview

The program that this document is dedicated to can be described in a single sentence as follows:

> SFS_CODE is a generalized Wright-Fisher style forward population genetic simulation program for finite-site mutation (and indel) models with selection, recombination (crossing-over/gene conversion), and demography across an arbitrary number of migrating populations.

This means that an entire population of individuals (and all their chromosomes) is followed generation by generation, from the beginning of the simulation to the time of sampling. This is contrary to coalescent simulations [such as ms; Hudson (2002)], where the history of a sample is simulated backward in time until its founder. SFS_CODE has the ability to simulate finite-site mutation models (meaning that some sites can receive several mutations). Nonetheless, SFS_CODE actually stores all mutations that are either segregating or fixed in at least one of the populations, so it can also act like an infinite-sites simulation program. However, its purpose is to generate a set of DNA sequences (an alignment) that can then be analyzed. This alignment, by the nature of the simulation, can therefore contain sites that have been the target of many mutations (as well as repeatedly being selected upon).

As described in further detail in subsequent sections, SFS_CODE allows the user to simulate highly detailed populations, with at least as much flexibility as ms. In addition to allowing for fairly complex demographic effects and migration (and/or admixture) schemes, SFS_CODE also allows the user to simulate coding versus non-coding regions, apply a distribution of selective effects to new mutations (including arbitrary models of dominance), generate domesticated populations, assume different male and female population sizes, linked and unlinked loci, sex and autosomal chromosomes, polyploids (haploid, diploid, or tetraploid), as well as a suite of built in or custom mutation models.

The basic algorithm used in this program is as follows:

1. Sample a sequence from the stationary distribution of the mutation model.
2. Burn-in a single population to mutation/selection balance.
3. Perform demographic and other evolutionary events.
4. Sample individuals from populations.

Each generation consists of the following components:

1. Produce each individual by randomly sampling a mother and a father from the previous generation (with replacement according to their relative fitness for their sex, unless simulating haploids, in which case there is no sex).
2. Randomly select individuals to migrate among populations.
3. Distribute a Poisson number of recombination/mutation events.

2 Citing this program

R. D. Hernandez. A flexible forward simulator for populations subject to selection and demography. Bioinformatics. 24(23):2786-7 (2008).

3 Getting Started

3.1 Obtaining the Program

`SFS_CODE` can be download from `http://sfscode.sourceforge.net/`. After following the download link, I highly recommend you to click the "Monitor this package" link (it looks like an envelope with a plus sign). You need to set up a free account through sourceforge.net, but will allow you to stay on top of updates. There are also two mailing lists that I encourage you to use: sfscode-users and sfscode-updates. The former allows users to email each other to ask questions about the functionality of `SFS_CODE`, and the latter is where I will announce new releases and bug fixes.

3.2 Compiling the Program

This section is only if you have downloaded the source code and wish to compile the program yourself. If you are using the web-based version of the program, then you can skip this section.

After obtaining the program (likely a file called "`sfscode_yearmonthday.tgz`"), move it to a folder on your computer. You can unzip the file by double-clicking it and hoping your computer knows what to do, or open a command-line terminal and type "`tar -xzvf file.tgz`". Inside this folder, you will now find a file called `makefile` and a few subdirectories (*e.g.*`doc/`, which contains this documentation; `sfs_code_DIR/`, which contains the `.c` and `.h` files that are `SFS_CODE`; etc.). The `makefile` will be used to compile all the programs provided with this distribution. It uses GNU's `gcc` compiler. Using your favorite command-line terminal (Windows users should download and install `Cygwin` from `http://www.cygwin.com` or another such application), and type `make`. This will create the directory `bin/`, which will contain the executables `sfs_code`, `convertSFS_CODE`, as well as any other programs in the current distribution.

If you get compiling errors, it is likely that either you do not have `gcc` installed. If this is true, then you either need to remedy the situation, or change the `makefile` to use your favorite compiler. If you are using `Cygwin`, you may need to update your version, making sure to install `gcc` (there are a series of boxes to click whilst updating the program). If you use a mac, then make sure you've installed the developer toolkit. Note, if you are planning to use the Intel compiler, you may need to edit the source code `sfs_code.c` by uncommenting the very first line (delete the "`/*`" at the beginning and "`*/`" at the end; this enables functions that Intel deems as "safe" but are not part of the standard `C` library, and will get rid of annoying warning messages). If you use a mac, further optimizations can be obtained by replacing `-O3` with `-fast` near line 11 of the `makefile`.

3.3 Usage: Arguments at the Command Line

`SFS_CODE` is a command-line program. If you have already compiled the program, then you should be ready to go. Change directory to `SFS_CODE/bin`. A full list of options can be found in Table 6 on page 56.

The basic command to run SFS_CODE is as follows:

```
./sfs_code <Npops> <Niter> [<options> [arguments]]
```

Where <Npops> is the total number of populations you want to simulate, and <Niter> is the total number of iterations you want to run. In this documentation, arguments and options that are enclosed in <angled brackets> are required, and those in [square brackets] are optional. Subsequently, those in both angled and square brackets can be required in some potentially optional instances (*e.g.*[<options>...], if you include anything after <Niter> then they must be options, which may contain required and/or optional arguments).

In SFS_CODE, all options have both a **long name** and a **short name**, except for timed events (beginning with '-T', described later, and only use the short name). For example, to set the mutation rate, you could use either "-t θ" or "--theta θ" to achieve the same result. Though both long and short names are case-sensitive, long names are of arbitrary length and tend to be more descriptive of the option. Short names are a single letter (so the options that were implemented later have short names chosen from the dwindling list of letters). Note that long names are preceded by two dashes ("--") while short names are preceded by only a single dash ("-"). Both the long and short names of all options are provided in Table 6 on page 56.

In the text of this document, I will provide templates for each option, as well as numbered examples. In option templates, I will first give the long name, then the short name in parenthesis, followed by the format of its arguments, as in the following pattern.

```
--long_name (-short_name) [arguments]
```

As a first example, the help menu can be obtained using the option

help menu

```
--help (-h)
```

This means you would access the help menu by typing "./sfs_code 1 1 -h". In this special example, the number of populations and number of iterations do not need to be specified, so you could just type "./sfs_code --help" or "./sfs_code -h".

Many parameters of the simulation can be set specifically for a single population (if multiple populations are simulated, see section 4.3) or for a single locus (if multiple loci are simulated, see page 8). These features will be indicated in an option template as follows.

```
--long_name (-short_name) [P <pop>] [L <locus>] [args]
```

This would indicate that one could specify a single population or a single locus. This P should not be confused with the italicized P, which refers to the ploidy number such that PN is the total number of chromosomes in a population.

4 Running SFS_CODE

The most basic simulation is the following:

Ex. 1. $ ./sfs_code 1 1

Typing example 1 (excluding the $, which just represents the bash shell; in Windows, you also might not need the "./" bit either) into the command prompt will result in running a single iteration of the default simulation. The default parameter values are given in section 12 toward the end of the documentation, and consists of simulating a coding sequence of length 5001 nucleotide base pairs from a "standard neutral" population of 500 diploid individuals, where the population scaled mutation rate $\theta = 0.001$/site with no recombination, from which a sample of 6 individuals (12 chromosomes) will be drawn. By "standard neutral" population, I am referring to a population that is devoid of every evolutionary force other than mutation and drift. The full list of default parameter values is given in the Default Parameters section below.

The **mutation rate** per site ($\theta = 2 \times P \times N_e\mu$, where P is the ploidy [i.e. 1 for haploid, 2 for a diploid, and 4 for tetraploid; see page 12]) can easily be increased to a value of 0.01 per site using the option

--theta (-t) [P <pop>] <θ>

mutation rate

as follows:

Ex. 2. $ ./sfs_code 1 1 -t 0.01

Recombination can involve both crossing-over and gene conversion (which may be GC-biased). Crossing-over is easy to incorporate using the following template (see section 4.5 for a longer discussion involving gene conversion):

--rho (-r) [P <pop>] [F <filename>] <ρ>

cross-over rate

where $\rho = 4N_e r$ is the population scaled rate of cross-over between adjacent sites for a diploid population (though the males in a population will never recombine on sex chromosomes, see page 10). There are two options. In the simple case, just specify the value of ρ for a uniform cross-over rate across all sites. For example, the following would simulate a standard neutral population with per site mutation and cross-over rates equal to 0.01.

Ex. 3. $ ./sfs_code 1 1 -t 0.01 -r 0.01

Alternatively, you can specify a generic recombination map (which allows for hotspots, see section 4.5.1) using --rho F <filename> <ρ>. This general recombination map will apply to both cross-overs and gene conversions. Note that this option is not available to CBSU web cluster users. The recombination file should contain the following information. **Note that this file has a new format as of February 2009**. The first line has one value, the number of points that the map is evaluated at. For example, in human genetics, the HapMap study produced a recombination map with a recombination rate evaluated at all HapMap SNP locations, so the number of points on the map is the number of SNPs contained in the region being simulated. Each additional line then contains two values: the basepair position, and the cumulative probability of a recombination event occurring before this point. The last line should contain the total number of basepairs simulated as the first entry, and 1.0 as the second entry, since with probability 1 a recombination will have occurred somewhere (if a recombination event occurs). The file does not need to contain information regarding loci (discussed later), that is automatically accounted for by the program.

Recombination can also be a **sex biased** phenomenon. To alter the proportion of recombinants (both cross-overs and gene conversion) occurring during male versus female meiosis, use the option

sex-biased recombination rate

--pMaleRec (-Y) [P <pop>] <p>

in which case recombination events will be assigned to the paternal lineage with probability p ($p = 0$ implies that recombination only occurs in females while $p = 1$ implies recombination is only in males. $p = 0.5$ is the default, meaning that recombination occurs in each sex uniformly). This does not alter the total number of recombination events that are distributed. More specifically, each generation, a Poisson number of recombination events are uniformly distributed to individuals in a population. For each chosen individual, the inherited recombinant chromosome will come from the paternal lineage with probability p, otherwise it will come from the maternal lineage.

multiple iterations

In general, you will want to do several (perhaps several thousand) simulations. Doing so requires some patience (this is a forward simulation, after all). However, **multiple simulations** can be performed iteratively by changing the parameter <Niter>. Doing multiple simulations this way is beneficial, as compared to running them all independently, because SFS_CODE is able to take advantage of all the effort that went into all the previous burn-in periods. After an extensive initial burn-in period, the population will be at stationarity. It is much easier to obtain a pseudo-independent draw from a population at stationarity than it is to reach stationarity. Figure 1 shows how this is done.

The default initial burn-in time is $5\times PN$ generations (where P is the ploidy, see page 12, and N is the initial simulated population size, see page 11), while subsequent burn-in periods are only $2\times PN$. You can change the initial burn-in time using

initial burn-in time

--BURN (-B) <burn>

and change the subsequent burn-in periods (for iterations > 1) using

subsequent burn-in times

--BURN2 (-b) <burn>

This would set the initial or subsequent burn-in times to <burn>$\times PN$ generations.

In SFS_CODE, it is also possible to simulate an **arbitrary number of loci** (linked or unlinked) of arbitrary length using the following option.

number of loci & length

--length (-L) <nloci> <L_1> [<L_2>...<L_{nloci}>] [R]

This option allows you to simulate <nloci>. The first locus will have length <L_1>. You can stop here to set all loci to the same length. Otherwise, you have two options. You can specify each of <L_2>...<L_{nloci}> to set the lengths of each locus, or if you have a repeating pattern (*e.g.*a short locus followed by a long one) you can specify a subset of lengths followed by the character 'R'. For example, if you want to simulate 4 loci, with lengths (500bp, 1kb, 500bp, 1kb), then you could use either of the following commands.

Ex. 4.
```
$ ./sfs_code 1 1 -L 4 500 1000 500 1000
$ ./sfs_code 1 1 -L 4 500 1000 R
```

Note that simulating long loci in SFS_CODEcan be a computational challenge. This is because SFS_CODEonly stores unique haplotypes at each locus, but if the locus is long then the number of unique haplotypes approaches the total population size. Instead, simulating multiple linked loci

can be beneficial. Included in the distribution is the perl script optimizeLL.pl. This script will allow you to enter relevant parameters of your desired simulation (L, N, θ, ρ, and selection parameters). It will then perform a heuristic grid search for the optimal partitioning of the desired locus. This can also be performed on your own using a cluster for potentially much faster results.

You can **change the linkage among loci** using the next option.

```
--linkage (-l) <p/g> <d1> [<d2>...<dnloci-1>] [R]
```

linkage among loci

The first argument to this option must either be ‘p’ or ‘g’, indicating whether the distance between loci will be be <p>hysical distance (in basepairs) or <g>enetic distance (recombination fraction). The second argument is the distance between the first two loci. This is all you need if you want all adjacent loci to have the same distance. Otherwise, (again) you have two options. You can either specify the distance between each pair of adjacent loci (i.e. provide <nloci>-1 values), or, if you have a repeating linkage structure you can specify a subset of distances followed by the character ‘R’. For **independent loci**, you can use “--linkage p -1” or “--linkage g 0.5”. As an example, consider simulating 2 independent genes, each having 4 exons with lengths as in example 4 that are equally spaced with 2kb introns. You could simulate this as follows.

Ex. 5. $ ./sfs_code 1 1 -L 8 500 1000 R -l p 2000 2000 2000 -1 R

Moreover, you can **annotate** each locus as being either coding or non-coding, and sex or autosomal. By default all loci are autosomal coding regions. If you would like to specify

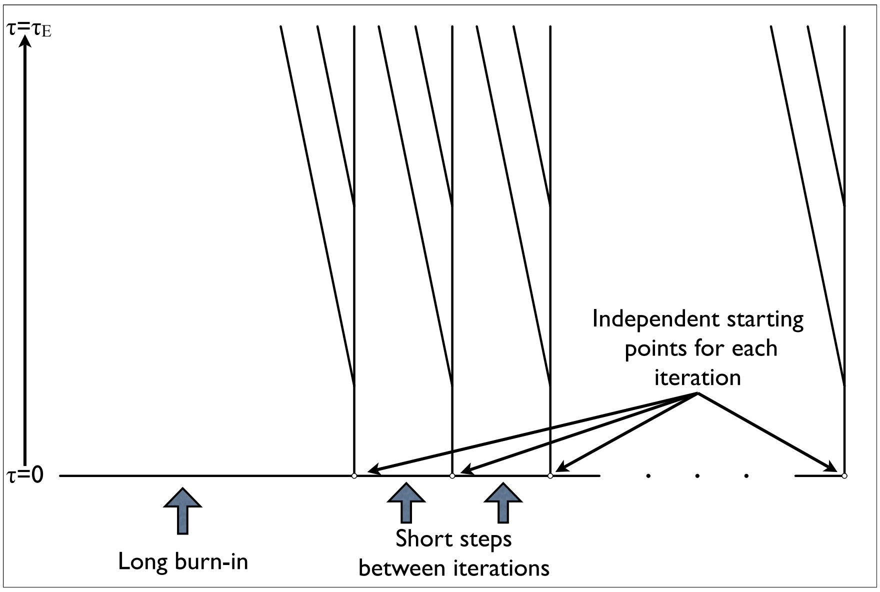

Figure 1: Simulating multiple iterations in SFS_CODE begins with a long burn-in time, followed by relatively short steps ($\sim 2PN$ generations) between each iteration. Ancestral information at the beginning of each iteration is stored, such that the each starting point is a random draw of a population at mutation/selection/drift balance (each iteration uses the burn-in of all previous iterations).

whether each locus is **coding or not**, use the following option:

annotate coding or non-coding

`--annotate (-a) [F <filename>] [<a1> [<a2>..<aR>] [R]]`

There are two options here. You can either specify the annotation of each locus using a file (in which case you would use `F <filename>`) or you can specify the the annotation for each locus individually, where $\mathtt{a}_i$ = 'C' or 'N' to indicate that the ith locus is coding or non-coding (respectively). If you want all loci to have have the same coding/non-coding annotation, just specify <$\mathtt{a}_1$>. Otherwise, you can either specify the annotation of all R loci, or specify the pattern to be repeated followed by the character 'R'. If you are using a file to annotate your simulation (an option that is not available to users of the CBSU web cluster), the file should contain the following information. On the first line should be a single number, the total number of loci, followed by a semicolon. Each additional line should correspond to the information about each locus (either the annotation of that locus or the physical distance between loci). To annotate a locus, a line should contain a coma-delimited list of 5 elements: the length of the locus, the nucleotides used for this locus (or blank to generate random sequence), 'N' or 'C' to indicate non-coding or coding, the type of selection followed by its parameters separated by a space (*e.g.*'0' for neutral, "1 <γ> <p_{pos}> <p_{neg}>" for a constant selective effect, etc.), and the constraint parameter f_0 for the given locus. Information on the last two entries can be found in section 4.2. You can also specify the physical distance between loci in this file (genetic distance is not allowed), but it is optional (default is to assume all loci are physically adjacent). To specify the distance, simply include the number of bases followed by a semicolon. If you are going to include inter-locus distances, they should be interleaved with the locus annotation. For example, consider the following annotation file:

```
3;
3000,,C,0,1;
1000;
3000,,C,0,1;
1000;
3000,,C,0,1;
```

This specifies that there are 3 loci, each is 3kb, will be composed of a random sequence, is coding, with no selective effects or constraint. Each of the loci has a 1kb gap between them that will not be simulated. Note that for the gap to be meaningful, you must also specify a recombination rate (as described on page 7). Note that if you do not specify an inter-locus distance, then it is assumed to be zero.

Important note: SFS_CODEdoes not allow mutations to stop codons in coding regions. They are not lethal mutations, they are simply never allowed to occur. However, in some instances, stop codons can creep into a coding sequence via recombination. SFS_CODEdoes not check for these rare occurrences.

To specify whether each locus is **sex or autosomal**, use the following option:

annotate sex/autosome

`--sex (-x) <x1> [<x2>..<xR>] [R]`

which has the same structure as option `--annotate`, but x_i = '0' or '1' to represent autosome or X-linked (respectively). Note that it is assumed that males never recombine on sex chromosomes (they are simulated to have an X and a Y chromosome). To generate pseudoautosomal regions, you can use two or more loci and make at least one of the loci autosomal. However, doing so would make the simulated Y chromosome useless. Note that in the output, the first chromosome (or pair if tetraploid) refer to the X and the second (pair if tetraploid) refers to the Y. This may not be a very good model of sex chromosomes in tetraploids.

Note that options `--linkage`, `--annotate`, and `--sex` must be specified **after** indicating the number of loci to simulate using option `-L` (unless there is only a single locus being simulated).

The **ancestral population size** used in a population genetic simulation is not as important as one might imagine (so long as all parameters are population-scaled, the actual size cancels). However, it can be changed from the default of 500 using the following option.

`--popSize (-N) [P <pop>] <size>` *population size*

This option would set the ancestral population size to the value `<size>`. For efficiency sake, the value you use should be kept as small as possible (but no smaller!!). The default is 500 diploid individuals, which should be sufficient for most purposes. However, if you are simulating a distribution of selective effects where the mean of the distribution is greater than the population size (in absolute value), then the entire population might go extinct. A realistic distribution inferred from human polymorphism data might induce such an effect.

It is important to note at this point that many parameters implemented in `SFS_CODE` can be updated at any time during the simulation. These are described in the next section as well as in section 4.9. A full list of parameters that can be changed at a specific time can be found in Table 6, and are indicated by an asterisk (*) next to the shortname.

4.1 Population Expansions and Bottlenecks

Natural populations fluctuate in population size, and any simulation program should accommodate this biological feature. However, it is often not necessary to simulate the exact trajectory of the population size, just the major trends (i.e. the time of an expansion, or the severity of a contraction along with the degree of recovery). `SFS_CODE` implements four types of **demographic events**:

1. set the population size to a **new value**:

   `-TN` <τ> `[P <pop>]` <N_{new}> *population size change*

2. change the population size by a **relative amount** ($\nu = N_{\text{new}}/N_{\text{old}}$):

   `-Td` <τ> `[P <pop>]` <ν> *relative size change*

3. allow the population size to start changing **exponentially**:

exponential growth

-Tg <τ> [P <pop>] <α>

4. or commence **logistic** growth/decay:

logistic growth

-Tk <τ> [P <pop>] <K> <r>

Each of these options begin with '-T'. This indicates to SFS_CODE that an evolutionary event will occur at a specific time (<τ>, the first argument). The next character (one of 'N', 'd', 'g', or 'k') indicates the type of demographic event (NOTE: only short names are accepted for timed events). The first argument for these options is the time parameter <τ>. Time is scaled by the effective size of the *ancestral population* (essentially the number of generations *since the end of the burn-in* divided by the number of chromosomes in the ancestral population). Next there is an optional parameter that would allow you to specify a specific population. If you want the demographic event to be applied to all populations (or you are only simulating a single population), then this is not necessary. Otherwise, if you only want to apply the demographic effect to population 0 (see description below on how to simulate multiple populations), then you would use 'P 0' here. Using the character 'P' in your command tells SFS_CODE that the next parameter is a population and not the value for the size change effect.

Finally, if you are using '-TN' include the **new size** of the population <N_new>. If you are using '-Td' include the **relative size** change <ν> = new size/current size (note that current size is NOT necessarily the ancestral size if you have multiple changes). If you are using '-Tg' include the **exponential** rate of growth/decay <α>. The parameter α determines the size of the population at time t by the equation $N(t) = N_0 e^{\alpha(t-\tau)}$, where time is scaled by PN_A (the number of chromosomes in the ancestral population, n.b. in a diploid population $P = 2$), τ is the time that the population size started changing, and N_0 is the size of the population when it started changing (not necessarily the ancestral size!). This implies that if you want the population to grow from N_0 individuals to N_F individuals in $(t-\tau)\times PN_A$ generations, you would invert the exponential equation to find $\alpha = \ln\left(\frac{N_F}{N_A}\right)/(t-\tau)$. If you are using '-Tk' for **logistic** growth, include the carrying capacity <K> (the final population size) and the rate to approach it <r>. For logistic growth, the size of the population at time t is determined by the equation $N(t) = \frac{KN_A e^{r(t-\tau)}}{K+N_A(e^{r(t-\tau)}-1)}$.

SFS_CODE is a forward simulation program, so it thinks about time going forward. You can think of the burn-in period as "negative time", with the simulation actually starting at time zero (when the burn-in ends), and progressing forward in generations. Rather than referencing a specific number of generations, however, time is referenced in terms of PN_A generations, where N_A is the ancestral (original) population size and P is the **ploidy** (if you are simulating a diploid population, then $P = 2$ [the default], while $P = 1$ for a haploid population and $P = 4$ is a tetraploid population). You can change the ploidy using the following option:

ploidy

--ploidy (-P) <P>

where P can be 1, 2, or 4. If P=4, you can specify either autotetraploid population or allotetraploid using

--tetraType (-p) <0/1> *type of tetraploid*

where 0 indicates auto- and 1 indicates allotetraploid.

Keep in mind that the time scaling does not change as the population sizes change (though the amount of evolution taking place each generation can be considerably different). This is similar to `ms`, but instead of having a diploid time scaled in units of $4N_0$ generations (with N_0 the size at the time of sampling), `SFS_CODE` would scale time in units of $2N_A$ generations.

In `SFS_CODE`, it is also necessary to tell the simulation program **when to end** using the option

-TE <τ> [pop] *ending simulation*

where again, time (τ) is scaled in units of PN_0 generations. In the simple applications above, the simulation actually ended when the burn-in period was over (i.e. at time $\tau = 0$). In general, you can end the simulation for any population at any time (useful for generating samples from now extinct populations, such as neandertal), but in most situations you will terminate the evolution of all populations when you sample at the end of the simulation. To be more specific, the simulation ends when the last evolutionary event takes place. The "-TE" option just allows you to put a place holder until a specific generation.

If you want to simulate a model for an *African* population of humans, you might consider a simple 2-epoch model, where there was a constant ancestral population size (N_{A}) which instantaneously changed by a factor $\nu = N_{\mathrm{C}}/N_{\mathrm{A}}$ some time τ ago (in units of $2N_A$ generations). A diagram of this model is shown in figure 2. To implement this model in `SFS_CODE`, you would consider time during which the population has its ancestral size as the burn-in period. At the end of the burn-in period, the population instantaneously grows by a factor ν, and maintains the new size for $2N_{\mathrm{A}}\tau$ generations, when the simulation ends. Abstractly, this is implemented in `SFS_CODE` as

Ex. 6. $ ./sfs_code 1 1 -Td 0 ν -TE τ

Notice that the demographic event actually occurs at time zero, with the population maintaining it's new size for τ units of time until the simulation ends. The parameters of such a model were inferred by Boyko et al. (2008) using synonymous SNPs across the human genome from an African American (AA) population. Our inferred demographic model is shown in figure 2. Simulating the AA demographic history using their inferred parameters is easy:

Ex. 7. `$ ./sfs_code 1 1 -Td 0 3.3 -TE 0.4377`

The equivalent command in `ms` would be:

```
./ms 12 1 -t 16.5 -eN 0.066 0.303.
```

Note that `ms` requires $\theta = 16.5$. This ensures that the ancestral population has $\theta = 5$, which is the case for the `SFS_CODE` simulations (θ/per site $= 0.001$ across 5kb).

A simple demographic model for European populations is a 3-epoch bottleneck model. This model is also shown in figure 2, and consists of an ancestral population size (N_{A}), a bottlenecked population size (N_{B}), and a current population size (N_{C}). In SFS_CODE, generations begin accumulating when the first demographic event occurs (i.e. $\tau = 0$, when the population decreases in size). The second demographic event occurs at the end of the bottleneck ($\tau^2_{\rightarrow} = 7703\text{gen.}/(2N_{\mathrm{A}}) = 0.48$), and the simulation ends at $\tau^{\mathrm{E}}_{\rightarrow} = 8577\text{gen.}/(2N_{\mathrm{A}}) = 0.54$. Given these parameters, this model is also straightforward to implement:

Ex. 8. $ ./sfs_code 1 1 -Td 0 0.722 -Td 0.48 5.27 -TE 0.54

The corresponding command in ms would be:

ms 12 1 -t 19.02 -eN 0.00728 0.19 -eN 0.0714 0.263.

You can **change the sample size** using the option

sample size --sampSize (-n*) [P <pop>] [R <τ_d> S <τ_1>]] <SS$_1$> [<SS$_2$>...<SS$_{\mathtt{Npops}}$>]

If you are only simulating a single population or you want to sample the same number of individuals from each population, then you can simply use "-n <SS>". If you want to set a specific sample size for each of n populations, use "-n <SS$_1$>...<SS$_n$>". Alternatively, if you just want to change the sample size of population i, then use "-n P i <SS$_i$>". Note that *individuals* are sampled, so if you simulate a diploid population (P=2), then 2 chromosomes will be printed at each locus for each individual. This option allows you to sample the population at arbitrary times by setting a timed event (see section 4.9) using -Tn. In this case, you must include the time of sampling ¡τ¿ (in population-scaled generations) and the sample sizes. You can set up recurrent sampling using R <τ_d>, where a sample will be taken every τ_d population-scaled generations after τ. Sampling will continue to the end of the simulation, or stop at an arbitrary time S <τ_1>. Sampling can be population-specific by including the P flag as before.

4.2 Distribution of Selective Effects

One of the many important components of a forward population genetic simulation program is natural selection. SFS_CODE assumes a simple multiplicative (the default) or additive model of genic selection by default. In the case of multiplicative selection, this means that the fitness of an individual is just the product of the fitness effects of each mutation they carry. In general, a new mutation will have fitness $1+hs$, where h is the dominance coefficient ($h = 1.0$ means codominance or genic selection) and s is the selective effect ($s > 0$ indicates positive selection, $s < 0$ indicates negative selection, and $s = 0$ indicates neutrality). An individual that is homozygous for such a mutation would then have fitness $(1 + s)^2$. The selection coefficient is related to the population scaled selection coefficient $\gamma = 2N_e s$. Because population genetic theory is generally based on inference of γ, SFS_CODE draws γ from a specified distribution (discussed below), then divides it by PN_{C}, the number of chromosomes in the population when the mutation arises (note that P is the ploidy, which is 2 for the default diploid population). SFS_CODE then uses s to determine the fitness of each individual, and normalizes by the mean fitness in the population.

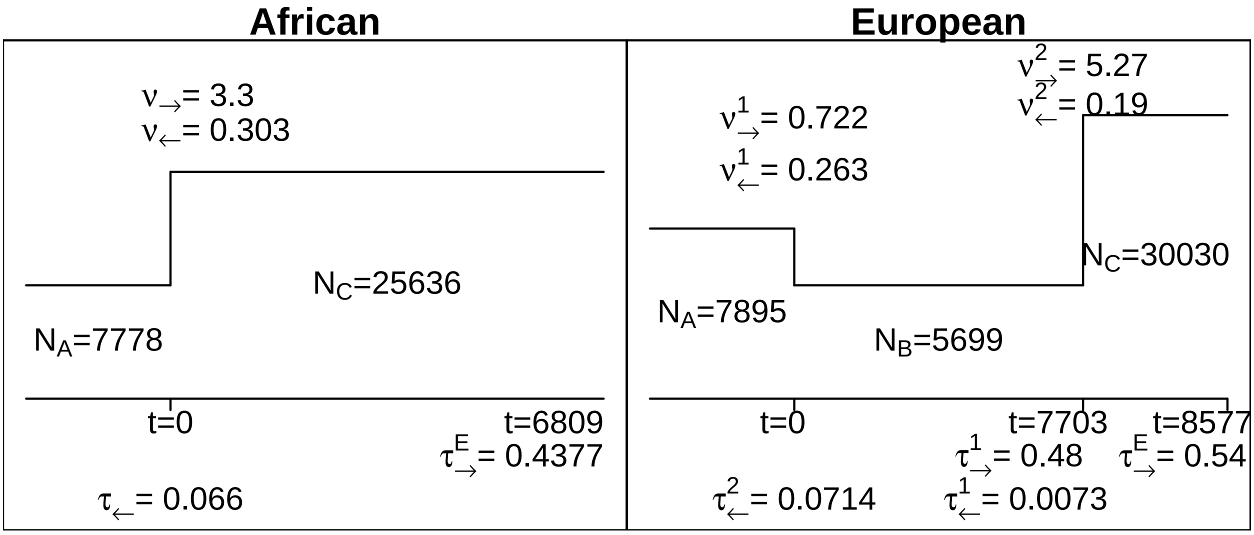

Figure 2: The simple demographic scenarios considered considered in section 4.1. Parameters (τ and ν) with subscript $\rightarrow$ are for `SFS_CODE` (forward time), while those with subscript $\leftarrow$ are for `ms` (pastward time). The horizontal axis represents time in generations (with $t = 0$ at the first demographic event). To obtain $\tau_{\rightarrow}$, divide the accumulated number of generations by $(2 \times N_A)$. To obtain $\nu_{\rightarrow}$ divide the new population size by the current population size at each transition. This methodology differs from `ms`, where the population size at time of sampling is generally the base. The number of generations and the effective population sizes for both populations were inferred by Boyko et al. (2008).

Instead of a multiplicative model of selection, you can simulate an additive model using the following command

`--additive (-Z)` *additive model*

with no arguments. In this case, the fitness of an individual is calculated as 1 plus the sum of the fitness effects of all mutations carried by the individual. Note that the additive and multiplicative models are very similar, and usually result in only subtle differences. A individual homozygous for a mutation with selective effect s will have fitness $(1+s)^2 = 1+2s+s^2$ under the multiplicative model while the same individual will have fitness $1+2s$ under the additive model (a difference of only s^2, which may be quite small if selection is not huge).

It is important to note that `SFS_CODE` only implements *shift* models of selection. This means that as soon as a selected mutation is fixed in the population, the fitness effect of the site returns to 1 (barring any other mutations that may have arisen at the same site). This avoids problems such as Muller's Ratchet, where the accumulation of deleterious mutations drives the population into the ground (though this can still happen if selection is strong enough and the population is small enough such that all individuals carry different lethal mutations). Shift models are also in contrast to models such as the *House of Cards* model that was developed by T. Ohta in the 1960s (whereby assuming a normal distribution of selective effects will eventually lead to the fixation of an allele with selective effect ≥ 8 standard deviations above the mean, at which point evolution nearly halts).

You can **specify the distribution of selective effects** using the following option:

selective effects

--selDistType (-W*) [P <pop>] [L <locus>] [H <type> [args]] [*F <alleleFreq> [w] [T [R <minFreq> <maxFreq> [S] [A [G <gens>]] [M <maxReps>]] [F [a] <file>]]] <type> [args]

Where <type> [args] are outlined in Table 1 on page 16, and the optional flags 'P' and 'L' allow you to specify a single population or locus (respectively, if simulating more than one population or locus). For 'L', it is also possible to specify 'O' or 'E' to indicate all "Odd" or "Even" loci, respectively, when you want parameters to alternate from one locus to the next. A fairly arbitrary model of dominance can be applied using 'H <type> [args]' (the types of distributions are analogous to the selection distributions in Table 1, except that there is only a single Γ-distribution for type=2. Note that in SFS_CODE, codominance is indicated by $h = 1.0$. The selection option can be used as a timed event (-TW) to enable selection on standing variation. When this is invoked, a time <τ> must be specified, and you can now use the flag F to indicate a particular frequency of the allele to be selected. This simulates a model of **selection on standing variation**. A SNP will be chosen randomly to match the indicated frequency, and the selection regime indicated by <type> [args] will determine the selection coefficient. For example, to specify a selection coefficient $\gamma = 2Ns = 100$, you would use 1 100 1 0. After specifying a frequency, you can include the flag w to force selection only only an indicated population (must use flag P <pop>). Note that the w flag is only functional when all other mutations are neutral, and that the output will be slightly wrong, because the selected mutation will have the given selection coefficient for all populations. Using the flag T, you can track the frequency trajectory of the selected mutation, and only print output when it achieves a particular frequency range using the flag R <minFreq> <maxFreq> (include S to stop the simulation the first time the allele is in the specified range). The simulation can automatically restart if the selected allele is lost or fixed using the flag A, or stop some number of generations after fixation using flag G <gens>. Note that with the A flag, if your frequency range includes 1.0, then the simulation will stop the generation the allele fixes. The simulation will attempt the achieve the desired sampling

Table 1: Selection: arguments for option --selDistType (-W)

<type>	[args]	description
0	∅	**Neutral** (gamma = 0 for all mutations).
1	<GAMMA> <p_pos> <p_neg>	**3-point mass model**. Single γ (> 0) for both deleterious and advantageous mutations. With probability <p_pos> the sign is positive, with probability <p_neg> it is negative, otherwise with probability 1-<p_pos>-<p_neg>, $\gamma = 0$.
2	<p_pos> <aP> <lP> <aN> <lN>	**Gamma (Γ) distributions**. With probability <p_pos> $\gamma \sim \Gamma$(<aP>,<lP>) (mean = aP/lP, var. = aP/lP^2), otherwise $\gamma \sim$ -Γ(<aN>, <lN>).
3	<mean> <var>	**Normal distribution**. Mean = <mean> and variance = <var>.
4	∅	**Advanced option**. Predefine distribution in file gencontextfreq.c, see text.

range 10,000 times then abort the mission, but you can update the number of trials using flag `M <maxReps>`. The frequency trajectory of the selected allele can be output to a file using the flag `F [a] <file>`, where `[a]` is included to append to an existing file.

As an example, to simulate rampant positive selection, where all new nonsynonymous mutations have $\gamma = 5.0$, you would use

Ex. 9. `$ ./sfs_code 1 1 -W 1 5.0 1.0 0.0`

To simulate a situation in which 70% of new nonsynonymous mutations are deleterious with $\gamma = -5$, 10% are advantageous with $\gamma = 5$, and the remainder are neutral, you would use:

Ex. 10. `$ ./sfs_code 1 1 -W 1 5.0 0.1 0.7`

To simulate selection on standing variation, where a neutral allele reached 5% frequency, and then became selected with $\gamma = 2Ns = 10$ and was sampled at frequency 80% in the population $0.5 \times 2N$ generations after selection began, you would use:

Ex. 11. `$ ./sfs_code 1 1 -TW 0 F 0.05 T R 0.75 0.85 A 1 10.0 1.0 0.0 -TE 0.5`

Note that the flag `-TE 0.5` (discussed on page 13) is necessary for selection on standing variation to function. If you want to simulate until fixation, specify the end of the simulation far off into the future and use the flag `A` to stop the simulation. If the end of the simulation occurs before fixation, then your selected allele will not be fixed!

For a more complicated scenario, in which you want a distribution of positive and negative selection, we have `<type>=2`, which implements a mixture of Gamma distributions ($\Gamma(\cdot)$), one that corresponds to positive values of γ and one that has been reflected across the y-axis to capture a distribution of negative values. For example, if you want to assume that 90% of new nonsynonymous mutations are deleterious with a selection coefficient drawn from $\Gamma(1, 1)$ (a simple exponential distribution) and the remaining 10% are advantageous and drawn from $\Gamma(50, 10)$ (having mean = 5 and variance = 0.5), then you could use the following example.

Ex. 12. `$ ./sfs_code 1 1 -W 2 0.1 50.0 10.0 1.0 1.0`

Note that for example 12, the distribution of deleterious effects reduces to an exponential distribution, while the distribution of advantageous effects has a mean and mode at 5. This mixture distribution is shown in figure 3. Of course if you simply want a Γ-distribution of negative selection (assuming no positive selection), then you can simply set `<p_pos>` = 0.

The fourth `<type>` (number 3), is a simple normal distribution. With a mean of zero, Cutler (2000) refers to the normal distribution of selective effects a model of positive selection. This is because on average, half of the new mutations will be advantageous, and a majority of the deleterious mutations will be eliminated.

The final `<type>` of model for the distribution of selective effects is an "advanced" option. For this option, you can create as complicated a distribution as you'd like in another statistical package (`R`, for example). This distribution can be discretized into an arbitrary number of bins of equal density (using the `quantile` function in `R`, for example). These

bins are then copied into the vector `fitQuant` that is stored in the file `gencontextrate.c`. After changing this vector, the program must be recompiled (this is the only reason that it is referred to as an "advanced" option... more realistically, it is a rudimentary option that requires more work, but provides the ultimate flexibility). This model is actually preferred to `<type>=2`, as it is much quicker to randomly sample from a discretized distribution than it is to draw from a mixture of Γ-distributions. However, population size changes cannot be accommodated with this option.

It is also possible to specify that one population remain a neutral population. This can be useful if you want to specify a common distribution of selective effects for all populations but one. This is done using

neutral population

`--neutPop (-w) <pop>`

When simulating multiple populations with selection, it is important to know that the selection parameters (excluding the type of selection) will be inherited from the ancestral population. This is to allow the effects of ancestral population size changes (described in the next section) to be propagated through to the daughter populations. If you want the parameters to be different, **you must use a *timed event*** to change their values (as described in section 4.9). An exception to this rule is the `--neutPop (-w) <pop>` option, which will force the indicated population to be neutral from the time it is created until the end of the simulation (barring any invocations of a subsequent timed event).

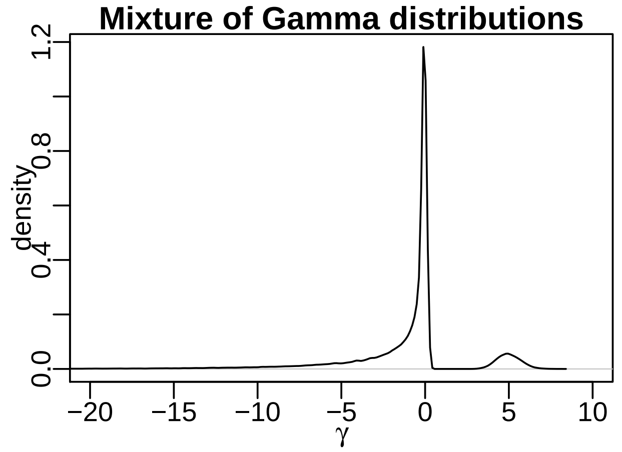

Figure 3: A distribution of selection coefficients, where 90% of new mutations would be deleterious with $\gamma \sim -\Gamma(0.231, 0.1279)$, and the remaining are drawn from $\gamma \sim \Gamma(50, 10)$.

Selective Effects with Demography

When population sizes change, the relative effect of selection changes (selection is stronger in a larger population). This effect is accommodated by altering the distribution of the population scaled selection coefficient. For constant (type 1), gamma (type 2), and normal (type 3) distributions this is easily accommodated since multiplying these distributions by a scalar has a known distribution. However, for the custom distribution of selection coefficients

(type 4), population demography cannot be accommodated. If a custom distribution of selection coefficients is used and the population sizes change, then the same distribution of γ will be used (thereby inflating/deflating s to maintain a constant value of γ).

Selective Constraint

In the way that Kimura outlined the *neutral model of evolution*, some proportion of nonsynonymous mutations are completely lethal, and never contribute to polymorphism. All other nonsynonymous mutations were completely neutral, and had no selective effect at all. As a result, it is often of interest to simulate data under such a neutral model (or allowing some proportion of nonsynonymous mutations to be lethal in general while the remaining nonsynonymous mutations follow the specified distribution of selective effects). This also generalizes to non-coding regions, where some proportion of mutations can be lethal. In Kimura's model, the parameter f_0 represents the proportion of neutral mutations. In SFS_CODE, you can adjust the **non-lethality parameter** using the following command.

--constraint (-c) [P <pop>] [L <locus>] <f$_0$>

non-lethal mutation rate

This option can even be used when simulating non-neutral models of evolution, as a way of signifying that only some mutations will contribute to polymorphism.

The way this option works, is that for each nonsynonymous or non-coding mutation, with probability $1 - f_0$, the fitness effect will be -1. This effectively sets the fitness of the individual to zero (as the fitness of the individual is defined as $1 + s$). This means that any mutations that are unique to this individual will also be lost in the next generation, as it will not pass on any of its gametes. All synonymous mutations are assumed to be neutral (i.e. none are considered lethal).

4.3 Multiple Populations

In the above examples, we have used exclusively a single population, with <Npops> = 1 as the first parameter into SFS_CODE. If we change this parameter, then we can simulate multiple populations. Note that populations are numbered from 0 through <Npops>-1.

There are two ways to create new populations. You can either have a speciation event or a domestication-style event. For a **speciation event**, one population will be split into two identical populations (equal size, etc.). To split population i into two populations (i and j) at time τ, you use the following template.

-TS <τ> <i> <j>

speciation events

For a **domestication event**, one population (i) will be split into two (i and j), but the second population will primarily be composed of individuals that carry a particular derived allele, chosen at random from all the alleles that have a specified frequency (within 5% of <allele_freq>). After choosing a particular allele from the founding population, SFS_CODE will randomly sample individuals that are homozygous for the allele. If there are not enough homozygous individuals, then it will choose from the heterozygous individuals. If there are

still insufficient individuals, then it will randomly choose non-carriers, until the specified population size, `<N>` is reached (note that `<N>` must be less than the size of the parent population `i`). The template for this option is as follows.

domestication events

-TD <τ> <i> <j> <allele_freq> <N> [locus]

If a `locus` is specified, then `SFS_CODE` will try to find an allele in that particular locus (not necessary if only simulating a single locus). If `locus` is not specified, then `SFS_CODE` will start at the center-most locus that is simulated. If there isn't an allele near the specified `allele_freq`, `SFS_CODE` will search adjacent loci until one is found. Failing to find any mutations at the specified frequency, `SFS_CODE` will select the allele that is closest in frequency.

Now that multiple populations have been initialized, it is essential to tell `SFS_CODE` when to **end the simulation**. This was mentioned above with regards to demographic effects, but is worth mentioning again. This is done using the familiar option -TE <τ> [pop]. As an example, say you wanted to simulate human polymorphism data with a chimpanzee outgroup (assuming a population scaled divergence time of $\tau = 10$ and an allopatric speciation event). You could use the following:

Ex. 13. `$ ./sfs_code 2 1 -TS 0 0 1 -TE 10`

This example would first generate a single population at stationarity during the burn-in. At the end of the burn-in ($\tau = 0$), the population would be split into two identical populations, which would evolve independently until the end of the simulation ($\tau = 10$).

As an example of a domestication event, consider a model for dog breed formation, where you also want to simulate the ancestral dog population. This model is characterized by a major bottleneck in the ancestral population followed by rapid growth. Then, after growing for some time, 2 new breeds (of size 100 and 10) are formed using alleles at frequency 0.1 and 0.01 (respectively) in the ancestral population. These new breeds are then simulated for $0.1 \times 2 \times 500 = 100$ generations.

Ex. 14.
```
$ sfs_code 3 1 -Td 0.0 P 0 .1 -Tg 0 P 0 2 -TD 2.5 0 1 0.1 100 \
  -TD 2.5 0 2 0.01 10 -Tg 2.5 P 1 10 -Tg 2.5 P 2 15 -TE 2.6
```

Let's walk through this example step by step. First, `sfs_code 3 1` indicates that we are going to simulate a total of 3 populations for 1 iteration. Next `-Td 0.0 P 0 .1` indicates that there is going to be a demographic event at the end of the burn-in period for population 0. This demographic event will shrink the population to 1/10th its size. After the major contraction, `-Tg 0 P 0 2` indicates that population 0 will start exponentially growing at a rate of 2 per generation (the backslash '\' indicates that the command stretches onto the next line and can be ignored). Then, after 2.5 units of time, two new breeds are formed from this ancestral breed. Population 1 is created by `-TD 2.5 0 1 0.1 100`, indicating that an allele at frequency 0.1 in the parental population was used to form a population of 100 individuals. Population 2 is created by `-TD 2.5 0 2 0.01 10`, indicating that an allele at frequency 0.01 is used to form a population of size 10. Both breeds then start growing at an exponential rate (population 1 at a rate of 10, while population 2 grows at a rate of 15). Then, after another 0.1 units of time (100 generations, or approximately 200-300 years), the simulation ends and we draw the default of 6 individuals from each population. This

simulation takes about 2.3 seconds on a mac Pro with dual quad-core 2.8GHz Intel Xeon processors and 6 GB RAM.

Sampling a Population at Several Time Points

If you are interested in looking at the dynamics of a process (say the frequency spectrum of a population just prior to an expansion, just after an expansion, and N generations after an expansion), you would want to sample a single population at several time points. There is no direct way of doing this in `SFS_CODE`, but there is a work around. You could use the `-TS` and `-TE` options to duplicate a population and immediately stop the simulation for one copy. For the example just mentioned, we could use the following command.

Ex. 15. `$ ./sfs_code 3 1 -TS 0 0 1 -TE 0 0 -Td 0 1 2 -TS 0.1 1 2 -TE 0.1 1 -TE 0.5`

In this example, we are effectively simulating 3 populations, but as soon as one population is created (as an exact duplicate of the ancestral population), the ancestral population is terminated, halting the simulation for that population and saving its state in memory until the end of the simulation, at which point it is printed.

Migration

Individuals are free to migrate to any extant populations. The migration rate matrix indicates the average number of individuals in each population that are composed of individuals from each of the other populations. For the migration matrix $\mathbb{M}$, the (i, j) entry $m_{i,j}$ represents the expected number of individuals in population i that came from population j (this is also referred to as the "backward migration rate matrix"). To set the migration rate, you would use the command `--migMat` (`-m`). There are three ways to set the values of the migration matrix, indicated by the first argument to the option being either '`A`', '`P`', or '`L`'. You can set **All entries** to be the same value `M`: *migration rates*

`--migMat (-m) A <M>` *all rates equal*

Note that this option specifies a symmetric island model, where the number of migrants into population i is M. So, for `<NPOP>`=3, there would be $M/2$ migrants from both of the other two populations. You can also set the migration rates explicitly **from one Population to another**:

`--migMat (-m) P` <$\mathtt{P_{to}}$> <$\mathtt{P_{from}}$> `<M>` *population specific*

which would specify that the average number of migrants into population $\mathtt{P_{to}}$ from $\mathtt{P_{from}}$ is `M`. Finally, you can **List the entire migration matrix**:

`--migMat (-m) L` <$\mathtt{M_{0,1}}$>...<$\mathtt{M_{NPOP,NPOP-1}}$> *list all matrix entries*

which would set each entry of the matrix. Note that the *diagonal entries are not specified.* For example, if you have 3 populations and want to use option 'L', you should specify all 6 entries: $M_{0,1}, M_{0,2}, M_{1,0}, M_{1,2}, M_{2,0}, M_{2,1}$ (unlike `ms`, you do not need the place holders for $M_{0,0}$, etc.).

In `SFS_CODE`, a Poisson number of individuals are chosen to migrate from population j to population i each generation with expected value $M_{i,j}$. Each migrant out of population j will be male with probability `pMaleMig`. You can set the **male migration rate** using

male migration rate

`--pMaleMig (-y) [P <pop>] <p_male>`

By default, $\texttt{p}_{\texttt{male}}$`=1-propFemale`, corresponding to the proportion of males in the originating population. By default, this is 0.5, but you can **change the proportion of females in a population** using

proportion of females

`--propFemale (-f) [P <pop>] <p_f>`

This can be set for all populations simultaneously, or for a given population explicitly.

One issue that is important to mention here is that while the population size of the daughter population will be set during the split, migration parameters that have been specified at the command line will not be updated. This only becomes an issue when the ancestral population changes size, and will only affect migration rates (other parameters are copied identically from the founding population). For example, if you specify a symmetric migration rate using the option `--migMat A` M_0, but you incorporate a 2-fold expansion in the ancestral population, then the migration rates will not be the same after the split. This is because the parameter $M_0 = 2N_0m$ doubles when the ancestral population expands. However, when the new population splits off, it will maintain the value of M_0. This would result in the migration rates being different between the two populations. One way to work around this is to set the migration rate at the time of the split using the *timed* option `-Tm`. The flags to this option are identical to above, except that the first flag is the time (scaled by PN_a generations, where P is the ploidy), see Table 6.

Admixture

Admixture is a demographic process that involves the mixing of populations. African Americans are an example of an admixed population, having genetic ancestry from both African ($\sim 80\%$) and Europe ($\sim 20\%$). Such an effect can be created in `SFS_CODE` using the following option:

Admixture

`--admix (-TJ)` <τ> <P> <N> <N_{pops}> <P_1>..<$P_{N_{\text{pops}}}$> <M_1>..<$M_{N_{\text{pops}}}$> [F <F_1>..<$F_{N_{\text{pops}}}$>]

Here, admixture occurs at a specified time (τ), which is why the short name begins with `-T`. In `SFS_CODE`, admixture creates a new population, allowing the ancestral populations to continue evolving. The new population is numbered P, will have N individuals (see discussion below regarding copying an ancestral population size) and is founded by N_{pop}

ancestral populations. The ancestral populations are indicated using P_1 through $P_{N_{\rm pop}}$. The proportion of ancestral population i contributing to the derived population is indicated by M_i ($1 \leq i \leq N_{\rm pop}$). If you wanted to have males and females to contribute different proportions to the derived population, let the M_i described before correspond to the MALE representation, and specify `F <`F_1`>..<`$F_{N_{\rm pop}}$`>` to indicate the female representation in the derived population.

Regarding the population size. If you want the derived population to have the same exact size as another population (population i, say), then you can set the population size (N) to $-i$. So if you want the derived population to have the same size as population 1, you would just enter `-1`. If 0 is entered for population size, the effective size will be made to equal population 0.

As an example, say you want to create an admixed population of 500 individuals from two ancestral populations that diverged $2 \times 2N$ generations ago. Suppose further that you wanted 80% of the males in the derived population to come from the first population, and only 20% of the females to come from the first population. You could use a command like this:

Ex. 16. `./sfs_code 3 1 -TS 0 0 1 -TE 2 -TJ 2 2 500 2 0 1 0.8 0.2 F 0.2 0.8`

This command would split population 0 at the end of the burn-in, then after $2 \times 2N$ generations, population 2 would be created.

4.4 Mutation Models

There are 6 mutation models built into `SFS_CODE`. The basic initiation of a mutation model is as follows.

```
--substMod (-M) <mod> [args]
```

substitution model

Equilibrium nucleotide frequencies can be specified using

`--baseFreq (-q) [P <pop>] <`π_C`> <`π_G`> <`π_T`> <`π_A`>`

equilibrium nucleotide frequencies

The default values are 0.25 for all nucleotides. The equilibrium nucleotide frequencies are incorporated into the mutation models described below, effectively allowing for much more flexible models. For example, by specifying equilibrium nucleotide frequencies, the JK69 model becomes the F81 model (Felsenstein, 1981), and the Kimura 2-parameter model becomes the HKY model (Hasegawa et al., 1985).

Table 2 outlines the models and arguments for this option. The most basic mutation model (`<mod>` = 0) was proposed by Jukes and Cantor (1969), and referred to as **JC69**. This model assumes that the rate of mutation is equal among all nucleotides. A simple modification of this model was proposed by Kimura (1980) to account for the observation that most mutations tend to be transitions (A↔G or C↔T). This model (`<mod>` = 2) adds another parameter (the transition/transversion bias, κ), and is referred to as the Kimura 2-parameter model (or just **K2P**). An extension of the K2P model would be to allow a

Table 2: Mutation models: arguments for option `--substMod`

<mod>	[args]	description
0	$\emptyset$	**JC69** model of equal mutation rates to and from all nucleotides.
1	<ψ>	**JC69+CpG** Simple model of hypermutable CpGs, where <ψ> is the non-CpG rejection rate.
2	<κ>	**Kimura 2-parameter** model, with <KAPPA> the transition-transversion bias.
3	<κ> <κ_{CG}> <ψ>	**K2P+CpG** combining model 1 and 2, allowing separate κ for C_pGs.
4	$\emptyset$	**ZG2003** the generalized K2P model, where each nucleotide has its own transition/transversion bias (all parameters inferred by Zhang and Gerstein (2003)).
5	$\emptyset$	**Context-Dependent** model, where the mutation rate at each nucleotide depends on both of its adjacent neighbors (all parameters inferred by Hwang and Green (2004)).
6	<a> <b> <c> <d> <e> <f>	**General Time Reversible (GTR)** model: $a-f$ are rate parameters, and base frequencies are set using `--baseFreq` (pg. 23) Tavaré (1986)).

transition/transversion bias for each nucleotide (i.e. the rate of A→G is not equal to the rate of C→T). Zhang and Gerstein (2003) fit the parameters of such a model to human data. This model has been implemented in SFS_CODE as <mod> = 4.

One feature of mammalian genomes is the presence of hypermutable CpGs (due to the deamination of methylated C's that are immediately 5' of a G). SFS_CODE implements a CpG extension to both the JC69 model and the K2P model (<mod> = 1 and 3, respectively). This is implemented by rejecting mutations at non-CpG sites with probability <PSI>. Given a non-CpG site is rejected, a new site will be picked to mutate until either finding a CpG or accepting a non-CpG site. Once accepting a site to mutate, it will either mutate to a new nucleotide randomly (in the case of <mod>=1) or to a transitional nucleotide at a rate equal to <κ> (in the case of <mod>=3). For substitution models 1, 2, and 3, the mutation parameters (ψ and κ) can also be set for a single population using the following option.

set the value of κ

`--KAPPA (-K) [P <pop>] <κ>`

set the value of ψ

`--PSI (-C) [P <pop>] <ψ>`

The most detailed model that is implemented in SFS_CODE is <mod>=5. This is a full context-dependent substitution model, where the site-specific rate of mutation depends on both of its adjacent nucleotides. This accounts for mutation rate variation due to CpGs as well as other context-effects found by Hwang and Green (2004). Conditional on picking a site to mutate, the replacement nucleotide will also depend on the flanking nucleotides.

Choosing a new site to mutate is done using an inverse-CDF method, where relative hit-probabilities are defined by the cumulative site-specific mutation rates. More generally, any trinucleotide substitution model can be used by updating the 64×4 rate matrix Q in the file `gencontextrate.h` and recompiling the program.

`SFS_CODE` also supports a general time reversible model (GTR: Tavaré (1986)) as `<mod>`=6. This model has 9 free parameters (3 frequency parameters and 6 rate parameters). When calling this mutation model, only the substitution rate parameters are entered after this flag, the frequency parameters must be entered using the `--baseFreq` option. The parameters of this model are not flexible, and cannot be changed for each population, or set at a specific time.

While `SFS_CODE` is based on simulating finitely many sites, it is also possible to simulate data under a pseudo-infinitely many sites model. It is pseudo because multiple hits can occur, but no more than one mutation will be segregating at a site at any given time. This is specified using the following option.

`--INF_SITES (-I)` *infinite sites model*

Mutation Rate Variation Across Sites and Loci

Context-dependent mutation models impose mutation rate variation along a sequence. However, not all species show evidence for such a mutation process (*e.g.Drosophila*), despite having mutation rate variation. For such species, mutation rate variation has in the past been modeled as a discretized Γ distribution across sites. `SFS_CODE` allows you to simulate under such a model, allowing both sites as well as loci to have a mutation rate scaled by a discretized Γ distribution (with mean 1). These are implemented in the following options.

`--rateClassSites (-V) [P <pop>] <`$\texttt{n}_{\texttt{classes}}$`> <`$\alpha$`>` *mutation rate variation across sites*

`--rateClassLoci (-v) [L <locus> <rate>] [ [P <pop>] <`$\texttt{n}_{\texttt{classes}}$`> <`$\alpha$`>]` *mutation rate variation across loci*

These options allow you to specify a certain number of mutation rate classes ($\texttt{n}_{\texttt{classes}}$), which will be drawn from a $\Gamma(\alpha, \alpha)$ distribution (having mean 1 and variance $1/\alpha$). Note that when modeling mutation rate variation across loci there are two routes to take. Instead of a Γ distribution, you can specify the rate at each locus directly using the `'L'` flag. Note that here as well as in other places, the letter `'A'` can be used for locus to give all loci the specified rate. When specifying the rate at each locus explicitly, the default for each locus is to have a rate proportional to the number of bases in that locus.

Important note: The mutation rate specified using `-t` (or the default rate if unspecified) and the total sequence length will still determine the total number of mutations that enter the population! The rates indicated using these options just determine the rate at which they are assigned to a given site/locus. For example, consider the following command:

Ex. 17. `./sfs_code 1 1 -t 0.001 -L 3 100000 1000 100000 -v L A 1 -v L 1 10`

The average number of mutations introduced each generation would be $\theta \sum_{i=0}^{2} L_i/2 = 100.5$. Without the `-v` option, the fraction of mutations that fall onto locus 0 would be

$L_0 / \sum_{i=0}^{2} L_i = 0.4975$. With the -v option, the fraction of mutations that fall onto locus 0 is $r_0 / \sum_{i=0}^{2} r_i = 0.083$. This option is useful when simulating recurrent hitchhiking models or background selection (see example section below).

Adding a Mutation at a Predefined Time

In some studies, it is useful to be able to add a mutation at a predefined time (for example, you may want to learn about the properties of a mutation that is a particular age). You can do this in SFS_CODE using the following option:

add a mutation ad a defined time

--mutation () <τ> [P <pop>] [L <locus>] [S <site>] [H <h>] [G <γ>] [F <file>]

Note that this option has no short name! By default, this option will introduce a mutation at a random location in a randomly chosen locus in a random population that is alive at the indicated time. However, if you have multiple populations, you can introduce the mutation in a certain population; if you have multiple loci, specify a particular locus, and also specify the exact site that will be mutated. Finally, the mutation will be assumed codominant ($h = 1.0$) and neutral ($\gamma = 0$) unless you specify a dominance coefficient (using H <h>) and selection coefficient (using G <γ>). This selection coefficient should be scaled by the population size at the time the mutation even is introduced.

It is also possible to specify a conditional trajectory for this mutation using a file (F <file>). The file should contain at least 2 columns. The first column should include a time, and the second column should include a frequency. Additional columns will be ignored. Note that the time column can either be discrete generation numbers (i.e., integers) or population-scaled values (i.e., floats). Population-scaled values are assumed to be scaled by $4N_e$, similar to existing trajectory simulators. You do not need to specify which type of file is included, as SFS_CODEcan easily distinguish the two. Note that if discrete generations are used, then it is assumed that the frequencies will be **monotonically increasing**, but if population-scaled units are used, then the frequencies will be **monotonic decreasing**.

This option can be used many times to introduce several mutations. Most options in SFS_CODE, if repeated, will void previous declarations, but this is an exception. You can introduce as many mutations as you want.

Important notes. 1) This option imposes a strict infinite sites assumption for the sites specified with this option. Any site set to receive a mutation will NOT be included with the usual mutation machinery that is used to introduce variation every generation. Of course, if you want multiple mutations to arise at the same site, you can specify that by repeating this option multiple times for the same site. 2) This option must be specified AFTER using the --length (-L) option if you are using anything but the default settings for the loci lengths.

This option works nicely with the --trackTrajectory option described on page 43, in the sense that if you want simulations where a mutation is a particular age and frequency at the time of sampling, tracking the trajectory can enable you to do this.

Keep in mind that while you are restricted to entering a specific time for a mutation event to occur, you can easily create a text file with random times you would like to consider (drawn from whatever distribution you desire), and write a shell script to repeat the simulations using each of the times listed in the file. For example, if you have a file `times.txt` that has 1 column and 5 rows with just the times you want mutations to be entered, you can use the following code at the command-line to generate the desired simulations:

```
for((i=1; $i<=5; i=\$i+1))
do
    T=$(head -n $i times.txt | tail -n 1);
    ./sfs_code 1 1 --mutation $T -o out_$i.txt;
done
```

4.5 Recombination: Crossing-over and Gene Conversion

Crossing-over and gene conversion are two important evolutionary forces that occur during meiosis. For the discussion that follows, I will focus explicitly on diploids, and assume that the process is analogous for tetraploids conditional on choosing the two parental chromosomes that will contribute to the inherited chromosome.

SFS_CODE has been written to handle cross-overs and gene conversion in way that approximates the biological mechanism to the best of my ability (while maintaining a simple model). Crossing over was introduced above on page 7. Using `-r` <ρ> alone results in a simple crossing-over model of recombination. One can add gene conversion using the following template:

`--geneConversion (-H) [P` <pop>`] [B` <B_{GC}>`]` <f> <λ> *gene conversion*

Here <f> denotes the relative rate of crossing-over (if r is the cross-over rate per generation, and g is the gene conversion rate per generation, then $f = r/(g + r)$). For example, $f = 0.5$ implies that crossing-over will occur during half of the recombination events. As outlined in figure 4, a section of DNA is excised from each strand in opposite directions from the double stranded break. The lengths of DNA excised are modeled as independent draws from a Geometric distribution with mean <λ> (*i.e.*, the total tract length has mean 2λ, which is not geometrically distributed). By default, gene conversion does not assume any base composition biases. However, it has been hypothesized that the biological process may be biased toward the repair of G and C nucleotides when present in a heterozygous state with an A or T nucleotide. To allow for such a bias, you can enter `B` <B_{GC}> before the f parameter to specify the degree of GC bias from 0 to 1, with 1 implying that GC alleles are always favored over AT alleles, 0 implying that AT alleles are always favored over GC alleles (not biologically realistic), and 0.5 (the default) implying that there is no bias. The parameters can be specified for a specific population using the now familiar `P` <pop> flag (which if used should be the first argument).

Note that one can simulate gene conversion without recombination by simply not specifying a recombination rate, or setting the recombination rate to 0 (*e.g.*, `-r 0`, which is the default, hence, not necessary). In this case, the value of f is interpreted as the population

scaled gene conversion rate per site $f = 4Ng$, which is completely analogous to the population scaled cross-over and mutation rates. Each generation, $fL/2$ gene conversion events would take place, where L is the total sequence length simulated.

In the case of crossing-over alone (non-zero recombination rate, ρ, with no gene conversion), a Poisson number of recombination events will be distributed with mean $\rho L/2$ (as is usually the case, described previously). In the case of gene conversion with crossing-over, the value of ρ entered in the `-r` option specifies the total recombination rate (gene conversion plus crossing-over), so each generation the number of recombination events will be Poisson distributed with mean $\rho L/2$. ρ can be thought of as the rate at which chromosomes that receive double stranded breaks are passed on. This model follows the diagram shown in figure 4, where 3 of the 4 chromosomes produced during meiosis do not involve any gene conversion at all (except in the case of BGC). Note that while the biologically sensible parameter space for f is between 0 and 1, you can specify $f > 1$ to allow crossing-over to occur more frequently than gene conversion. While this is not biologically realistic, other programs (such as `ms`) allow it. In such cases, $f = r/g$.

After drawing the total number of recombination events (both crossovers and gene conversion), they are distributed amongst the N offspring individuals uniformly. However, whether the recombinant chromosomes are inherited from the maternal or paternal line can be specified using the `--pMaleRec (-Y)` option described on page 8. Each offspring chromosome receiving at least one event will randomly choose a site for the event to occur (either uniformly or according to the recombination map supplied using the `-r F <file>` <ρ> flag). Gene conversion always takes place 5' of the site selected, and crossovers always occur immediately 3' of the selected site.

For simulations with biased gene conversion, heterozygous AT→GC mutations in the conversion tract (purple in figure 4) will be copied with probability B_{GC} (this represents a biased repair in favor of the GC allele, which is the derived state). Heterozygous GC→AT mutations in the conversion tract will NOT be copied with probability B_{GC} (again, representing a biased repair in favor of the GC allele, which happens to be the ancestral state). For other types of mutations that are heterozygous in the conversion tract, only those carried by the donor chromosome (blue in the figure) will be copied.

An important note regarding gene conversion: The locations of breakpoints and the conversion tract lengths are chosen independently. If the conversion tract extends beyond the beginning of the simulated locus, it will continue onto the previous locus (if the distance to the previous locus is specified in physical units and the tract is long enough). If there are no loci before the chosen break point or the distance is specified in genetic distance, conversion will stop at the beginning of the locus. Gene conversion will not extend beyond a single adjacent locus. For example, if you simulate 10 loci each 50 bases long, then the maximal tract length will be 100 bases. (If there is a situation in which this is not sufficient, please notify me at the email address above.) Together, these assumptions effectively reduce the mean length of gene conversion tracts in small simulated regions. It is easy to approximate the effective reduction by calculating the mean length of the tract that falls outside the locus. For this calculation, assume that the gene conversion tract length is a single draw from a geometric distribution with mean λ. Letting L be the length of the locus, $G(i|\lambda)$ be the geometric probability of drawing a tract length i given a mean length λ, the mean tract

length outside the locus is approximately

$$\sum_{i=1}^{L} iP(i) \tag{1}$$

where $P(i)$ is the probability of i bases falling outside the locus, which is approximately

$$P(i) \approx \frac{G(i+1|\lambda) + \cdots + G(i+L|\lambda)}{L}.$$

Expanding the sum in equation 1 and collecting terms results in the following simpler equation

$$\sum_{i=1}^{L} \binom{i}{2} G(i|\lambda). \tag{2}$$

For the case of a 5000 base locus with mean tract length 250 bases, the effective reduction is 12.4 bases. This was confirmed by 1.5 million simulations, which yielded a mean resulting tract length of 237.05, the distribution of which is geometric (more specifically, a censored geometric; MWU p-value = 0.8836). This information is only useful for comparing `SFS_CODE` to other simulation programs which first draw the length of the gene conversion tract then draw a starting position conditional on fitting that length (or in the case of an infinite sites simulator, where the tract length always fits within the simulated locus). For a proper comparison, one would need to reduce the tract length in the other programs by the amount in equation 2. Because it is necessary to allow gene conversion events to overlap multiple loci, such conditioning is impractical in `SFS_CODE`.

Note that because gene conversion and cross-overs are generated simultaneously (as compared to the coalescent simulator `ms` (Hudson, 2002) and other programs that simulate them independently), the parameters used in `SFS_CODE` will be different. For example, to compare `SFS_CODE` to `ms` with crossing-over and gene conversion, one needs to use the following key. For $f_{\text{sfscode}} \leq 1$:

$$\begin{aligned} \rho_{\text{ms}} &= \rho_{\text{sfscode}} f_{\text{sfscode}} \\ f_{\text{ms}} &= \frac{1 - f_{\text{sfscode}}}{f_{\text{sfscode}}} \end{aligned}$$

For $f_{\text{sfscode}} > 1$:

$$\begin{aligned} \rho_{\text{ms}} &= \rho_{\text{sfscode}} f_{\text{sfscode}} \\ f_{\text{ms}} &= \frac{1}{f_{\text{sfscode}}} \end{aligned}$$

Of course since the gene conversion tract lengths in `ms` and `SFS_CODE` are not drawn the same way, the resulting distributions will not be the same, but see the Comparisons and Expectations tab on the project website (`http://sfscode.sourceforge.net`) for a direct comparison of the two programs.

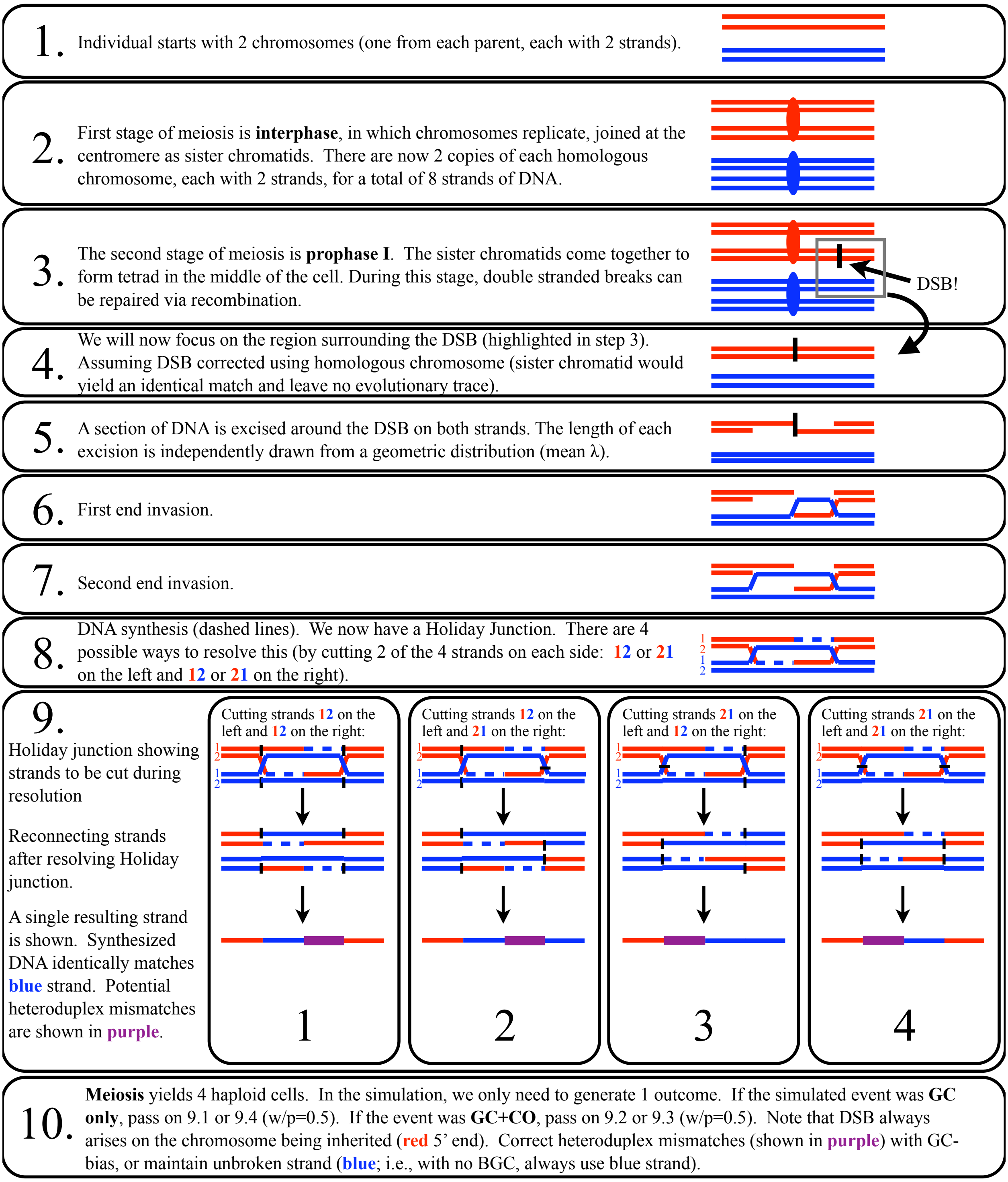

Figure 4: The simulated process of recombination (crossovers and gene conversion). Purple segments are the "conversion tracts" where heteroduplex mismatches may occur, which are repaired toward the blue chromosome (i.e., the unbroken strand) if there is no GC-bias. If there is GC-biased gene conversion, then AT↔GC mutations are repaired toward the GC allele with probability B_{GC}. Note that $f = 0.5$ would result in choosing among 9.1-9.4 equally likely, while $f = 1$ exclusively chooses either 9.2 or 9.3, and $f = 0$ exclusively chooses either 9.1 or 9.4.

4.5.1 Recombination Hotspots

In the current version, recombination hotspots are allowed through the use of the `-r F <file> <ρ>` option described on page 7 (but this option is not available on the CBSU web cluster). This option allows you to enter the name of a file that contains the explicit parameters of your hotspot model. A basic model might correspond to a simulation of a 5kb sequence with a single 200bp hotspot in the middle that has a relative intensity 10-fold higher than the background rate. The file would include the following lines.

```
3
2400 0.353
2600 0.647
5000 1.0
```

There are 3 sequence intervals: 0-2399, 2400-2599, and 2600-4999, having recombination probabilities 0.353, 0.294, and 0.353 (respectively). These probabilities correspond to a 10-fold larger rate for the middle 200 bases (*i.e.*, $0.294/200 = 10 \times 0.353/2400$). Using this model, the recombination map can be arbitrarily complex. It is important to note that the default operation of `SFS_CODE` is to simulate coding regions, thereby rounding the input number of bases in a locus to the nearest whole codon. If you want to disable this, you must use the flag `-a N` as described on page 10. Note that you can alter the recombination rate later in the command-line using another invocation of the `-r [P <pop>] <ρ>` option, such that different populations can have different rates of recombination. One also needs to specify the overall recombination rate as normal, since the only thing that this recombination map affects is the place where recombination events occur, not the distribution of the number of events.

4.6 Insertions and Deletions

`SFS_CODE` also allows you to simulate insertions and deletions (indels) from two different length distributions (*i.e.*, allowing shorter and longer indels). The model for such events is rather crude. They are treated just like mutation events, but can only occur in regions that have been annotated as non-coding (this requires the use of the –annotate (-a) option described on page 10), and will be given the same distribution of selective effects as mutations within the locus that they arose in. Insertions are inserted and deletions are deleted independently of one another, with each event affecting a number of nucleotides that are drawn from either a geometric distribution or poisson distribution (specified by the user). The command for implementing indels using a geometric length distribution is as follows:

`--indel (-u) [P <pop>] <INS`$_{\texttt{rate}}$`> <DEL`$_{\texttt{rate}}$`> <mean_length>` *indels*

The command for implementing indels using a poisson length distribution is as follows:

`--longIndel (-U) [P <pop>] <INS`$_{\texttt{rate}}$`> <DEL`$_{\texttt{rate}}$`> <mean_length>` *longIndels*

Where in both cases $\texttt{INS}_{\texttt{rate}}$ and $\texttt{DEL}_{\texttt{rate}}$ specify the insertion and deletion rate (respectively), and `mean_length` is the mean length of an event (assumed to the the same for both insertions

and deletions). The rates for insertions and deletions should be thought of as analogous as θ for mutations. Each generation a Poisson number of insertions and deletions will be drawn with mean 1/2 the specified rate times the total sequence length.

Because a geometric distribution has a mode at 1, it is best used for simulating short indels (such as a mean of length 3). The poisson distribution, on the other hand, is roughly symmetric about the mean (for large values, a normal approximation suffices). This poisson distribution is more useful for modeling indels that arise due to ALUs and LINEs. See section 8.2 for a description of how the output file reports indels.

4.7 Inversions

`SFS_CODE` allows you to simulate inversions with a length distribution given by a poisson model. Like indels, the model for inversions is rather crude. They are treated just like mutation events, but can only occur in regions that have been annotated as non-coding (this requires the use of the `--annotate (-a)` option described on page 10), and will be given the same distribution of selective effects as mutations within the locus that they arose in. Inversions swap the order of a stretch of nucleotides with a length drawn from poisson distribution. The command for implementing inversions is as follows:

inversions

`--inversions (-z) [P <pop>] <INV_rate> <mean_length>`

Where $\texttt{INV}_{\texttt{rate}}$ specifies the inversion rate and `mean_length` specifies the mean length of an event (respectively). The rate of inversions should be thought of as analogous to θ for mutations. Each generation a Poisson number of inversions will be drawn with mean 1/2 the specified rate times the total sequence length. See section 8.2 for a description of how the output file reports indels.

4.8 Selfing and Generation-Effects

`SFS_CODE` generally assumes that all populations are randomly mating (subject to their relative fitnesses). However, in plant species in particular, mating is not random, such that an individual may be more likely to self-fertilize than to mate with another (an ultimate form of inbreeding). To accommodate this, `SFS_CODE` allows the user to specify a selfing rate, `s`, for each population using the following option.

seljing rate

`--self (-i) [P <pop>] <s>`

For a population with selfing rate `s`, $\texttt{s}N$ individuals in the each generation will be produced by selfing, while the remainder will be produced by randomly selecting two parents (who may end up being the same by chance).

Moreover, when simulating multiple species, it will not always be the case that they will have the same generation time. For example, today, humans have a longer generation time than most other primates (especially the non-apes). To account for this, `SFS_CODE` provides a generation effect option.

`--GenEffect (-G) <pop> <G>` *generation effects*

For the generation effect, `G` must be an integer (≥ 1 or ≤ -2). If it is positive, then the indicated population will experience `G` rounds of mating each generation. If `G` is negative, then the indicated population will only have a round of mating every `|G|` generations. For example, setting `G`=2 would shrink the generation time by half (leading to 2 rounds of random mating every generation), while setting `G`=-2 would double the generation time (leading to one round of random mating every second generation). At least one population must have `G`=1.

4.9 Changing Parameters Over Time

In `SFS_CODE`, many of the parameters can be changed at any time during the course of the simulation. Table 6 outlines all the options that have been implemented in `SFS_CODE`, and any option that has an asterisk in the short name (third column) can be changed (or initiated) at any time using the following option.

`-T<short_name> <τ> [args]` *timed events*

This means that if you are, for example, studying domesticated rice, and want to model the transformation to a primarily selfing organism, you might consider a population which starts with a low selfing rate, but $2N$ generations ago became 99% selfing. This could be achieved as follows.

Ex. 18. `./sfs_code 1 1 -TE 1 -i 0.2 -Ti 0 0.99`

Example 18 would simulate data assuming the selfing rate was 0.2 until the burn-in time ended, at which point the selfing rate would change to 99%. The simulation would then end after another $2N$ generations.

When using `-T*`, the option retains all the functionality as described above and in Table 6. For example, consider simulating 2 populations, that diverged $10{\times}2N$ generations ago (i.e. human-chimp divergence). Suppose you wanted to model recent positive selection (*e.g.*within the last $2N$ generations) in the human genome while ancestral populations and chimpanzee are completely neutral. You might try the following example.

Ex. 19. `./sfs_code 2 1 -TS 0 0 1 -TE 10 -TW 9 P 0 1 5 1 0`

Example 19 would generate 2 populations, which split at time $\tau = 0$, and evolve independently for $10{\times}2N$ generations (`-TE 10`). However after having diverged for $9{\times}2N$ generations, all new nonsynonymous mutations in population 0 would be advantageous with $\gamma = 5$. In this case, the command "`-TW 9 P 0 1 5 1 0`" literally means change the distribution of selective effects at time $\tau = 9$ for population 0 to `type`=1, γ=5, `p_pos`=1, and `p_neg`=0.

Keep in mind that timed events only work with the short names, so you could not use `-TselDistType`, for example.

5 The Simulated Population Size *Sometimes* Does Not Matter

One challenge you will face when running forward simulations, is to pick an effective population size. In coalescent theory, this is a non-issue, as the results have been derived for limiting cases when the population size tends to infinity while parameters tend to zero, such that the product stays constant (isn't it nice that all parameters in population genetics are scaled by the population size?). However, in forward simulations, the actual population size used can become an issue, depending on what you are trying to model. Specifically, recurrent hitchhiking (with strong selection) and background selection (with a large deleterious mutation rate) can be dependent on the simulated population size you choose.

However, in most situations, the actual population size doesn't matter. In general, you should do a few simulations with larger and smaller population sizes to show that the population size used does not affect your simulations. Failing to do so could lead to a false interpretation of the results (particularly when there is rampant selection, see Uricchio and Hernandez (2014)). However, it is always helpful to use the smallest population size possible, as this will make the simulations run quicker. If you are simulating recurrent hitchhiking models, you can use the associated `sfs_coder` (from the package page at `https://sourceforge.net/projects/sfscode/files/sfs_coder/`) that implements Uricchio and Hernandez (2014) to optimize the population size given your parameters under a desired error tolerance. While we have not formally shown that the simulated population size matters for background selection simulations, it will probably matter when the burden of selection is strong.

We will consider **varying the effective population size**. This is done using the following option.

```
--popSize (-N) [P <pop>] <size>
```

We will consider populations of size $N \in \{250, 500, 1000, 5000, 10000\}$. This should give us an idea of what is going on. We will consider just a couple of statistics: the distribution of the total number of polymorphisms and the average site-frequency spectrum (SFS). We will evaluate populations of constant size, as well as populations that have recently either grown or shrunk 10-fold (magnitude of change $\nu = 10$ or 0.1, approximately $0.1 \times 2N$ generations ago). We will also consider neutral models as well as selection under both `selDistType` 1 and 2 (`-W 1 5 0.1 0.8` and `-W 2 0.1 50 10 0.23 0.1279`, respectively). Considering just the case of no recombination yields 45 combinations (5 population sizes $\times$ 3 demographic models $\times$ 3 selective effects). The general command line looks like the following example (note that we are sampling 20 diploid individuals, or 40 chromosomes).

Ex. 20. $ ./sfs_code 1 2000 -n 20 -N <N> -Td 0 <ν> -TE <τ> -W <type> [args]

Figure 5 summarizes the results. As you can see, for these demographic and selective effects, **the effective population size used has no impact on the distribution of SNPs or the SFS**. For the cases shown here, the only reason the curves do not perfectly overlap is because 2000 simulations is insufficient to capture the true distribution. A very similar figure was generated (but not shown) with recombination ($\rho = 0.01$/site) with identical results.

In addition to recurrent hitchhiking and background selection simulations, exponential growth in a small population size may not work well. In a small population, the time over which exponential growth takes place may not allow enough generations to have an accurate relative population size at the end of the simulation. Imagine a smooth exponential curve. With a small simulated population size, you are approximating that smooth curve with potentially very few points, thereby altering the dynamics.

6 Sampling From an Extinct Lineage

Recent advances in the extraction of ancient DNA has lead to the partial sequencing of the Neanderthal genome. This allows us to gain further insight into homo relatives, and actually learn more about our own species. One of the many questions that gains a lot of interest is whether or not humans mated with Neanderthals, and whether there is any evidence for or against it in our genomes. Understanding whether or not current statistical methods will have the power to detect evidence of such mating (or how much migration there would have had to have been to detect it) lies crucially in the hands of population genetic simulations.

Assuming that you've been able to simulate more than one population, sampling from an extinct lineage is actually dead easy (pun completely intended). You must simply "kill" one of the populations using the option `-TE <tau> [pop]`. Take the following example.

Ex. 21. `$ ./sfs_code 2 1 -TS 0 0 1 -TE 0.1 0 -TE 0.5`

During the burn-in, a single population would reach stationarity. At the end of the burn-in, the populations would allopatrically split (`-TS 0 0 1`). After $0.1 \times 2N$ generations, population 0 would effectively die (simulations for this population would stop). All the individuals from this population are still in memory, but evolution in this population ceases (all individuals would, quite literally, remain frozen in their prehistoric state). After an additional $0.4 \times 2N$ generations, the simulation completes. At completion, the default of 6 individuals will be sampled from the extinct lineage (population 0) and 6 individuals will be sampled from the other population (you could consider this the human population).

For a slightly more detailed simulation, consider the human-Neanderthal-chimpanzee alignment, where only a single individual is chosen from each species. This could be implemented as follows.

Ex. 22. `$ ./sfs_code 3 1 -TS 0 0 1 -TS 8 1 2 -TE 9 2 -TE 10 -n 1`

Step-by-step, this would perform a single simulation of 3 populations (`./sfs_code 3 1`). At the end of the burn-in, the ancestral population splits into two populations (*e.g.*the chimp and human-Neanderthal ancestor: `-TS 0 0 1`). After $8 \times 2N$ generations, humans and Neanderthals split (`-TS 8 1 2`). After another $2N$ generations, the Neanderthal population suddenly goes extinct (`-TE 9 2`). Finally, after a total of $10 \times 2N$ generations, the simulation ends, so we sample 1 individual from each species (including the extinct one).

Rather than have the Neanderthal population suddenly go extinct, it might be more useful to have them die out at an exponential rate, or something. Just as in section 4.1, we could add a growth (or death) rate of population 2 to -2 at time $\tau = 8.5$ using `-Tg 8.5 P 2 -2`.

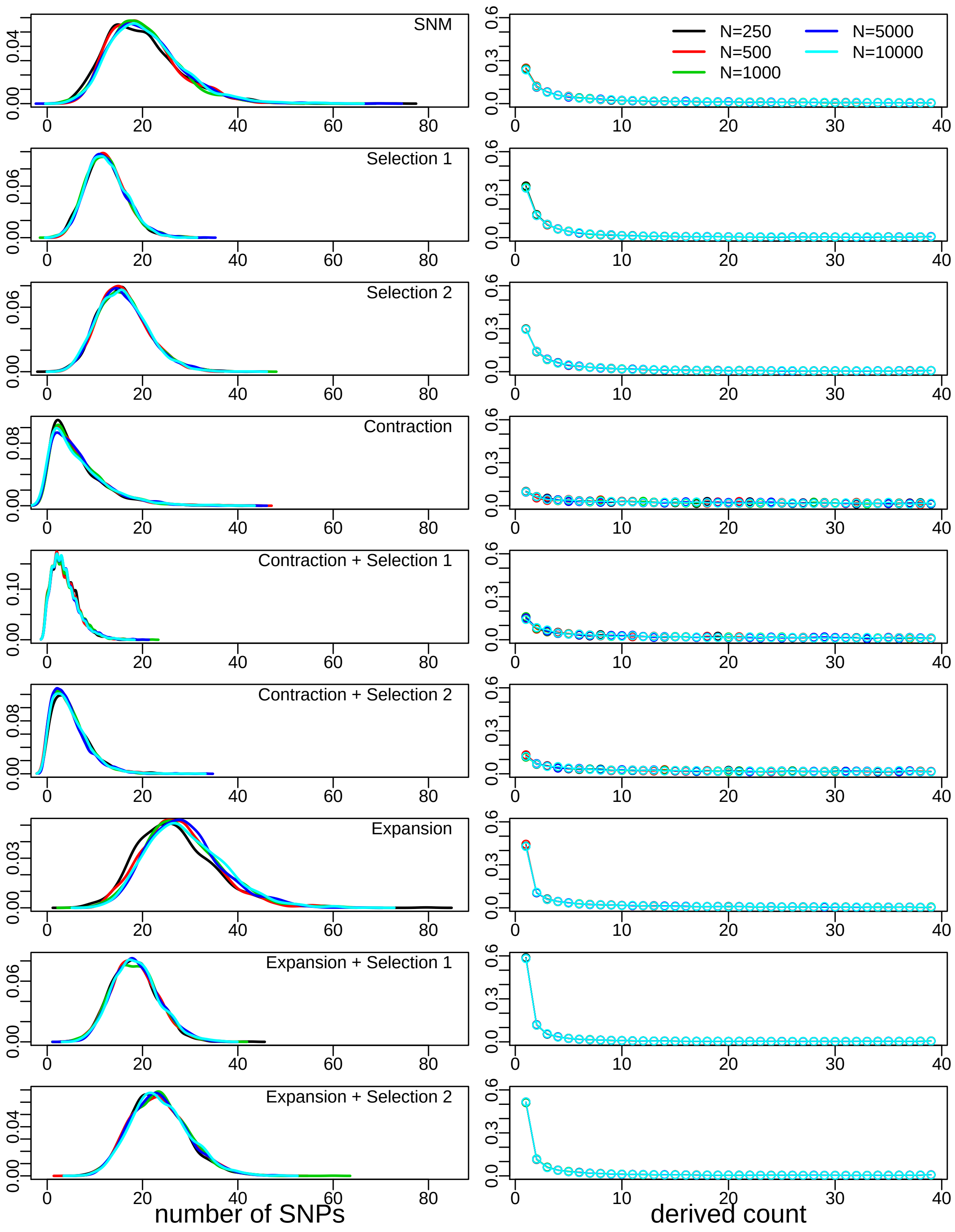

Figure 5: The non-effect of the effective population size in SFS_CODE. Each panel has 5 curves, corresponding to different values of N_e (shown in legend in the upper-right plot). Each row corresponds to a different set of assumptions (demography/selection). Left is the distribution of the number of segregating sites, and the right is the average SFS across 2000 simulations.

After simulating data, one could use convertSFS_CODE to analyze patterns of shared haplotypes, patterns of ancestral and derived alleles, etc. One could also consider humans and Neanderthals to be two populations with very high rates of migration, but negative selection acting strongly on the Neanderthal population until they are "bred out".

7 Using SFS_CODE on a Cluster

7.1 Your own Cluster

Any large scale simulation study cannot be performed without a large number of processors. Even coalescent simulations require a cluster in some situations, such as when using approximate likelihood techniques [*e.g.*Hernandez et al. (2007a); Caicedo et al. (2007)]. However, if you do not have access to a cluster, or are unfamiliar with how to run jobs on a cluster, then this section will be insufficient for you (try the next section, which deals with using the CBSU web cluster at Cornell University).

Being a forward simulation, SFS_CODE works well when identical jobs are sent to multiple processors, as they are all run independently. However, because the burn-in period takes a considerable amount of time, it is often beneficial to run several iterations on each processor (to take advantage of the short successive draws after the burn-in, as shown in figure 1). It therefore becomes a balance between the number of iterations to run on each processor versus the number of processors at your disposal to provide the most efficient results.

Let's consider the case where you want to run 2000 iterations of a single simulation, and you have access to 10 processors. The simplest way to complete the task is to split it up into 10 different jobs, each of which runs 200 iterations. Each of the 10 jobs can then be submitted to a different processor and run simultaneously. Of course the output from each processor has to be written to a different file (using `-o` option), since different processors cannot write to the same file simultaneously. When all the processors are done and have written their output, you would then need to concatenate all 10 output files into a single file for analysis.

Included in the distribution are two files: `makeTask.pl` and `runTask.sh` which will be found in the `Examples/` directory. `makeTask.pl` is a perl script that can be used to partition your simulations into a set of independent tasks. There are comments in the file itself that should help you to get this file configured for your particular simulation. Basically, you need to add parameters that are constant across all the simulations you want to run in a way that is similar to "`$THETA`", and put the parameters you want to vary in an array like `@RHO`. When you run the script at the command line using `perl makeTask.pl`, it will create a numeric folder for each set of parameters that you entered. `runTask.sh` is a shell script that can be used on a cluster running the Sun Grid Engine. This file must be submitted from the directory that you want your output to be printed to. It will produce a number files that equals the number of tasks in the `tasklist` file created by `makeTask.pl`.

Setting the Seed

When running simulations on several processors simultaneously, there is a very nontrivial probability that some jobs will start at the same time. These jobs would then have the same seed for the random number generator, and would therefore produce *exactly* the same results. This is very bad, every simulation must have a different seed. For any given simulation, you can **change the seed** using the following option.

random number seed

`--seed (-s) <value>`

Depending on the configuration of your cluster, you can generate a `TASKFILE` with several lines, each line containing the entire `SFS_CODE` command, but with a unique seed (as described in the previous section). In the latter situation, you could use another program (written using `MPI`) to dynamically distribute each of the tasks across the available processors (not provided, but these so called "master-slave" algorithms are commonly used and can be downloaded from other sources), or use the simple array job shell script `runTask.sh` described in the previous section.

Setting the seed is also useful if you ever want to reproduce a set of simulation results exactly.

7.2 Using `SFS_CODE` on the CBSU Web Cluster

*** Unfortunately, this option is only available to current employees at Cornell. ***

`SFS_CODE` has a dedicated webpage at `http://cbsuapps.tc.cornell.edu/sfscode.aspx`. From here, it is possible to submit your simulation jobs to the cluster hosted by the Cornell University Computation Biology Service Unit (CBSU). The interface is simple. Enter your email address (to have a link with simulation results emailed to you), and a single set of `SFS_CODE` arguments. All you have to do is indicate the total number of populations you want to simulate (`Npop`), the total number of successive simulations that you would like to run on each process (`Niter`), the total number of repetitions you want to run (`Nrep`), and the total number of nodes you want to use. Then, copy-paste your command-line arguments into the text box. A total of `Nrep`×`Niter` simulations will be run automatically, each with a different seed. You can only paste a single line into the text box. Running several different sets of simulations requires submitting several jobs.

The command will then be distributed across the requested number of processors. Upon completion, output will be concatenated into a single file, zipped, and stored on a server until downloaded (a link to the location will be emailed to you). Depending on the number of jobs in the queue, your job may take up to a few days to begin.

The CBSU web clusters have a 24 hour time limit. This means that any job that you submit to the cluster must be completed within 24 hours. It is good practice to become familiar with the length of time that your job will take by running a few iterations locally (also helpful to ensure that your command-line works). You could then multiply the average amount of time per job by the total number of tasks you wish to perform and divide by the total number of processors. If this is greater than 24 hours, consider splitting the job into two or more sets.

8 Understanding the Output

A lot of information is stored during the course of an SFS_CODE simulation that will be useful in many different situations (yet useless in others). Unfortunately, this makes for a lot of output. In order to make the output file as concise as possible, a fairly complicated format is needed. However, I've also produced convertSFS_CODE, a program that will convert the SFS_CODE output to a more useful format (see next section). However, it is important to know all of the information that is contained in the output in the event you want to perform a type of analysis that has not yet been implemented in convertSFS_CODE.

8.1 No indels or inversions

An example of the output when not simulating indels or inversions looks something like the following:

```
./sfs_code 1 2 -L 2 66
SEED = -382034166
//iteration:1/2
>locus_0
GTTCCAGGAAGCTGGACAGTCTCTTATGGCGACATGGTAAATAAATTTGCGGTCCTGAAATCGGGT
>locus_1
AATGGGTTAGATTATGATTATGTGCATCGTCTTTACACGAGTGGAGTCATTCGACTTTTCGTACTA
Nc:500;
MALES:3;
0,A,24,-237,0,TTA,A,1,Y,N,0.0,2,0.1,0.8;
//iteration:2/2
>locus_0
TTTTCAGTCTGTTTTGTCAAAGATTATTCTTTTGGGCTCCTCACGCACCTTAAGAAGTGTATATAC
>locus_1
TACGCTATCAACTACAATATACATAGTGTGGTTTTCGATGGCCTTAGGTCAGTTGACCTACGTAAC
Nc:500;
MALES:3;
1,A,28,-523,0,GTG,A,1,V,E,0.0,2,0.1,0.3;
```

The first line has the command line used to call SFS_CODE. In this case I've asked SFS_CODE to generate two iterations of a single population, with two loci, each of length 66 basepairs. The second line includes the value of the seed for the random number generator (this can be used to reproduce the results, though the version used to generate this output may be different from the one you are using so you may not be able to reproduce this output exactly). The third line starts the results of the simulations. Each iteration that is simulated starts with "//iteration:", followed by the iteration number and the total number of iterations being performed. The fourth line starts a fasta-style representation of the nucleotide sequences at each locus. The next line reports the final population size (N_c) of each population in a comma delimited list terminated with a semi-colon. The next line ("MALES:") provides the *index* for the first male that was sampled (*not the number of males*

in the sample), in this case indicating that individuals 3, 4, and 5 are male while individuals 0, 1, and 2 are female (note that these are diploid individuals, so chromosomes 0-5 belong to females and chromosomes 6-11 belong to males). The next line starts the information regarding every mutation that contributes information to the sampled sequences in a comma delimited list terminated with a semi-colon. There are at most 20 mutations per line, so the list of mutations can span several lines. The information provided for each mutation are as follows:

1. locus that the mutation arose on (zero-based)
2. 'A', 'X', 'Y', indicating Autosomal, X-, or Y-linked mutation, respectively
3. position of mutation in locus (zero based)
4. generation mutation arose (negative for mutations arising during burn-in)
5. generation mutation fixed in population (or time of sampling if segregating)
6. ancestral trinucleotide (middle nucleotide mutated, NOT CODON)
7. derived nucleotide
8. 0 or 1 for synonymous or nonsynonymous (respectively; 0 for non-coding)
9. ancestral amino acid (single character representation; '`X`' for non-coding)
10. derived amino acid (single character; '`X`' for non-coding)
11. fitness effect (this is s, NOT $\gamma = PNs$)
12. number of chromosomes (n) that carry the mutation

13-... comma delimited list of the n chromosomes carrying mutation

Each mutation event is terminated with a semi-colon. The list of chromosomes carrying each mutation is reported as a decimal: $p.c$, where p is the population number (zero based) and c represents the chromosome number in that population (also zero based). Take the mutation event reported in the first iteration: "`0,A,24,-237,0,TTA,A,1,Y,N,0.0,2,0.1,0.8;`". This indicates that it occurred at the first locus (zero), which is autosomal, at position 24. This mutation arose 237 generations before sampling (i.e. 237 generations before the burn-in period ended), and was segregating at time of sampling (sampled at time 0). This mutation was a nonsynonymous mutation having no fitness effect, and was carried by two chromosomes (1 and 8) in population zero (the only population simulated).

Mutations that fix in the population during the burn-in period are not recorded by default. To report mutations that went to fixation during the burn-in, use the following option

report mutations fixed in burn-in

`--ReportBurnFixed (-R)`

If you are running a simulation, and you aren't sure whether the program is totally stuck or just taking a long time, you can print a note every generation to watch the status. This is like a ghetto status bar. There is no short name for this option.

`--printGen`

print generations

Generally speaking, if you want to track fixations while simulating a single population, use the `-TE` option (those that fix during the burn-in are generally unreliable, but sometimes necessary if you enter a reference sequence using `-a F <file>` and want to track all changes). Mutations that are fixed in the sample from population p will be reported as p.-1. It is possible to distinguish mutations that are fixed in the sample but segregating in the population using the 4th entry (the generation it supposedly fixed). If it "fixed" at the end of the simulation, then the fixation time will be the same as segregating polymorphisms. This can only happen if it was still segregating in the population (as random mating would not yet have occurred).

It is generally encouraged to retain the ancestral sequence (just in case you want to go back and re-analyze some previous simulations and need the actual sequences; *e.g.*to setup the observed [using parsimony as compared to the true] McDonald-Kreitman tables). However, they can take up a lot of space in the output, so you can exclude ancestral sequences using the following option.

`--noSeq (-A)`

removing ancestral sequence from output

By default, output will be printed to the screen. If you would rather it be written to a file, you can use the following option.

`--outfile (-o) [a] <file>`

sending output to a file

The optional character '`a`' would allow you to append to a file rather than overwriting it. When multiple iterations are run, and output is being directed to a file, the progress of the simulation will be printed to the error file. By default, the error file is the screen, but this can be changed using the following option

`--errfile (-e) [a] <file>`

sending error messages to a file

This is also useful for keeping track of any error messages that arise (such as when figuring out what might have gone wrong with a set of command line arguments).

It is important to note that when simulating multiple populations, if a single mutation fixed independently in multiple populations at different times (due to the mutation arising in the ancestral population or migrating into another population), it will be listed multiple times in the output. This allows you to analyze the dynamics of ancestral polymorphisms in the face of differential demographic forces in daughter populations, but also adds a complication to the output file. As a result, some of the functions implemented in `convertSFS_CODE`(described below) may not function properly.

If many loci are being simulated, but you only care about a subset of loci, you can restrict the output to those loci using:

`--printLocus <locus>`

print individual loci

Note that this option can be repeated an arbitrary number of times to print out any number of loci.

8.2 With Indels and/or Inversions

When simulating indels and/or inversions, more information is required. Namely, we must specify the type of event, the number of bases involved, and the new DNA (if inserted). This is accommodated by allowing entry 8 to take on the value 'i' if the event was an insertion, 'd' if the event was a deletion, and 'v' if the event was an inversion. Entry 9 now has the number of bases involved in the event (no longer the ancestral amino acid, since there was no such thing). If the event was an insertion, entry 10 now has the new nucleotides, OTHERWISE THERE IS NO ENTRY 10. In the case of a deletion or inversion, entry 10 corresponds to the fitness effect, entry 11 corresponds to the number of chromosomes in the sample that carry the event, and entries 12 and on represent the comma delimited list of chromosomes carrying the event.

For an insertion, the new DNA is placed immediately after the base indicated in the third entry. Likewise, in the case of a deletion, the DNA deleted starts after the base indicated in entry 3. For example, for a deletion with the third entry having a value of '0', an eighth entry of 'd' and a ninth entry of '2', then the second and third bases in the sequence would be deleted.

The other minor change to the output is the ancestral and derived nucleotides. They are pretty much useless for any of the three events. For insertions, the ancestral allele is a '-' while the derived nucleotide is a '+'. The opposite for deletions. For inversions, the ancestral nucleotide is 'f' and the derived allele is a 'r' (indicating forward and reverse). The flanking nucleotides represent the flanking nucleotides at the time of the event at the site indicated. Take the following as an example:

```
./sfs_code 1 1 -L 1 50 -t 0 -n 1 -z .01 10 -a N -u .01 .01 5
SEED = -636193008
//iteration:1/1
>locus_0
CGTCATTGCTCACTACAGTCGCCTGCAAAGCTCGCGGTATGAACCTCTGA
Nc:500;
MALES:0;
0,A,2,-1687,0,G+C,-,d,1,0.0,1,0.0;
0,A,5,-714,0,CfC,r,v,5,0.0,1,0.0;
0,A,20,-1541,0,C-T,+,i,2,AA,0.0,1,0.0;
```

There are 3 events, one deletion, one inversion and one insertion. All events are carried by chromosome 0.0, and none are carried by chromosome 0.1. Using convertSFS_CODE (described in section 9) to generate the alignment of the two sampled chromosomes would produce the following sequences:

```
>it0pop0ind0locus0
CGTATCTCGTACTACAGTCGAACCTGCAAAGCTCGCGGTATGAACCTCTGA
>it0pop0ind1locus0
CGTCATTGCTCACTACAGTCGCCTGCAAAGCTCGCGGTATGAACCTCTGA
```

Looking at the events in order, the first event is a deletion indicating that chromosome 0.0 should be missing the fourth nucleotide (remember, positions start counting at 0, and the base following the indicated position is deleted). This corresponds to the nucleotide 'C', which is missing in chromosome 0.0. The second event was an inversion starting after position 5 and incorporating 5 nucleotides. Counting the characters in the second haplotype, we see that the nucleotides `TGCTC` should be involved in the inversion, and we therefore find the nucleotides `CTCGT` in positions 5-9 in the first haplotype. The third event is an insertion at position 20. Note that this position refers to the position in the ancestral sequence. As such, the second haplotype (which carries no events) has the nucleotides `CGCC` while the first haplotype has the nucleotides `CGAACC` (notice the addition of 2 '`A`' nucleotides, as was reported in the tenth entry of the original output.

8.3 Population-wide Allele Frequencies

In `SFS_CODE`, one often obtains a small sample of individuals from a larger pool of chromosomes that form the underlying population. Sometimes it is of interest to know the actual allele frequencies of each mutation not only within the sample, but also within the population as a whole. This can be done using the following option.

`--popFreq (-F) [a] <file>` *population frequencies*

This will print (or `[a]`ppend) to the `file` each mutation on a separate line. Each line will contain: the locus carrying the mutation, the site of the mutation within the locus, the generation the mutation arose, and the type of mutation ('`s`'ubstitution, '`i`'nsertion, '`d`'eletion, or in'`v`'ersion) followed by the ancestral and derived alleles (respectively). This will be followed by a tab delimited list containing the frequency of the mutation in the sample from population 0, the frequency in all of population 0, the frequency in the sample from population 1, the frequency in all of population 1, etc. This information can be combined with the information in the general output file from `SFS_CODE` to access most necessary information.

This file will also include mutations that are segregating in the population but were not contained in the sample. This is useful for understanding the effect of sample size on SNP discovery.

It is important to note that this option is not implemented on the version that is running on the CBSU web cluster (see section 7.2).

8.4 Tracking the Trajectory of an Allele

`SFS_CODE` allows the user to output a file with the trajectory of the frequency of all (or some) mutations each generation of their existence. You can initiate this option using the following command:

`--trackTrajectory [T <τ>] [P <pop>] [L <locus>] [S <site>] [R <min_freq> <max_freq>] [A [G <gens>]] [M <max_reps>] [F [a] <file>]` *track frequency trajectory*

This option is extremely flexible, and the details are important to explain, because this is among the most complex options available in `SFS_CODE`. First, all options are optional, and therefore all of them require a special flag to signify which option you are entering. ORDER IS IMPORTANT. You cannot swap flags, otherwise the command will not be processed properly. The first flag, `T`, indicates whether you want to start tracking the trajectory at a specific time (as always, scaled by $2N$ generations). By default, all mutations are tracked from the beginning of the simulation (*i.e.*, during the burn-in). Second is a population flag, `P`. By default, all populations are tracked, but you can track just a single population. Next is a locus flag, `L`. By default, all loci are tracked, but you can track just the mutations in a single locus. Next is a site flag, `S`. By default all sites are tracked, but you can specify a single site. Note that if there are multiple loci, and you do not use the `L` flag, then the indicated site will be tracked in all loci. Next, you can specify a desired final population-wide allele frequency range with `R`. If you want to generate simulations where at least one site ends up in a narrow frequency range, you can use this option. For example, if you want an allele to be at 1% frequency (±0.1%), you would use `R 0.009 0.011`. Next is a flag, `A` will automatically restart the simulation if all mutations being tracked go to fixation or loss. This is useful if you want many simulations with a particular site to be near frequency X, since a lot of time can be wasted if you continue simulating after the allele is no longer segregating. Moreover, if you are interested in simulating a fixed number of generations after the last population to fix the allele, use the next flag, `G` followed by the number of generations (NOTE: not population-scaled!). Note that if you want a very improbable scenario, many simulations will be rejected. By default, `SFS_CODE` will give up after 10,000 tries. You can change this default using flag `N`. Since it is often important to know how many tried it took to obtain a sample with your desired sampling frequency, the output file is adjusted slightly, with the total number of tries printed at the end of the "//iteration:1/1;X". In an unlikely scenario, X may be large, and a sort of probability for the scenario can be obtained from the number of successes and failures. Finally, the trajectories are not printed by default. This is because you may want to condition on the final frequency of an allele, but don't want to actually keep the trajectory. To print the trajectory to a file, use the flag `F`, and include the desired file name (include the lowercase `a` before the file name if you want to append to a file).

Note that the output format has changed to include the iteration as of version 07/25/2013. If an output file is generated, it will have at least 10 columns. Each line represents a different mutation event or generation. The entries of each column are as follows:

1. Iteration
2. Generation
3. Locus
4. Site
5. Generation the mutation arose
6. Ancestral nucleotide

7. Derived nucleotide
8. Fitness of mutant
9. Number of derived copies in population 0
10. Total number of chromosomes in population 0

...

If there are multiple populations, then the last two columns will be represented for each population (*i.e.*, if there are 2 populations, there will be 11 total columns: 9-10 for population 0, 10-11 for population 1).

Important note. Nothing is printed if a site is fixed or lost in all populations. This means that if a mutation arises in generation 10 and is lost or fixed in generation 20, then nothing will be printed for this site for generations 0-9 or 20-[end of simulation]. If there are multiple populations, and the site is polymorphic in at least one population, then the site will be printed.

8.5 Report the True Genetic Ancestry of Each Site

When simulating data with migration and/or admixture, it is sometimes of interest to know the true ancestry of a base being simulated for all individuals. This information can be output using the following option:

```
--trackAncestry [a] <file>
```

true genetic ancestry

The output file will be created (or appended to if you include the lowercase letter `a`), and have 1 line for each chromosome sampled. Each line begins with a chromosome identifier (*e.g.*, `pop0ind0chr0`, `pop0ind0chr1`, etc.), and is followed by a space-delimited list of integers indicating the population ancestry of each site simulated. Loci are concatenated, so it is your responsibility to parse the lines correctly.

Mechanistically, this option starts with all sites having population 0 as their ancestral state. When the population splits (say, `-TS 0 0 1`), all individuals in the newly formed population will now have ancestry pointing to population 1. If an individual in population 0 is replaced by a migrant fro population 1, then this individual in population 0 will have ancestry pointing to population 1. If there is admixture, then the individuals in the derived population will have ancestry pointing to the population from which they came. If there is a recombination event between two chromosomes that have different ancestry, then the ancestry after the breakpoint will swap. This creates a mosaic pattern of genetic ancestry that is commonly discussed in the admixture mapping literature.

8.6 Output in VCF format

As of the August 2015 release, `SFS_CODE` now allows data to be exported directly in VCF format. This can be accomplished using the following command:

Table 3: KEY=value pairs for INFO column in `--VCF`

KEY	value
NS	Number of sampled individuals.
AF	Allele frequency across all samples.
AXY	Indicates Autosomal, X-chromosome, or Y-chromosome
GA	Generation variant arose.
GF	Generation variant fixed (or sampled).
5P	5' nucleotide in sequence.
3P	3' nucleotide in sequence.
NSYN	1 if nonsynonymous, 0 if synonymous.
AA	Ancestral amino acid (if coding sequence).
DA	Derived amino acid (if coding sequence).
H	Dominance coefficient.
S	Selection coefficient.

Output VCF

`--VCF`

There are no parameters to this command, and it doesn't have a short name. The VCF file will be printed to the screen by default, or output to a file using the `-o` command (p41). So that there is no loss of information in creation of the VCF output, all the relevant details of the normal `SFS_CODE` output are stored in the "INFO" column (see Table 3). The header lines include the targeted VCF format (`##fileformat=VCFv4.2`), the date the simulations were run (`##fileDate=20150815`), the command (`##command=./sfs_code 1 1 --VCF`), the seed (`##SEED=-279262962`), the iteration (`##iteration=1/1`; though it is not clear that multiple iterations can easily be supported in VCF format), the locus (`##locus_0=ACGT...`; unless `-A` is used), the current population sizes (`##Nc=500;`), the position male samples start (`##MALES=3;`), and finally the variants, preceded by the usual header. Note that individuals are indicated by their population (p) and index (i) as Np.i. Note that `--VCF` only works for diploid individuals, and chromosomes are always perfectly phased.

9 Using `convertSFS_CODE` to Generate Useful Data

The output produced by the program `SFS_CODE` is fairly concise, but is not the easiest file to parse. I have therefore provided the additional program `convertSFS_CODE`, which takes the output from `SFS_CODE`, and converts it to a format that might be easier for you to use. These include various summary statistics, as well as the format required for the program STRUCTURE [*e.g.*Falush et al. (2003)], and an output analogous to format used by the coalescent simulation program `ms` (Hudson, 2002), among others. The basic usage of `convertSFS_CODE` is as follows:

```
./convertSFS_CODE <input_file> <option [args]>
```

The <input_file> to `convertSFS_CODE` is the output file from `SFS_CODE`. The options available are outlined in Table 4. As an example, consider generating a human-neandertal-

chimpanzee alignment using a single chromosome from each. You might consider the following slightly modified version of Example 22.

Ex. 23. `./sfs_code 3 1 -L 1 66 -TS 0 0 1 -TS 8 1 2 -TE 9 2 -TE 10 -n 1 -o out.txt`

This would generate a single simulation of 3 individuals for a locus of length 66 basepairs. You could then use `convertSFS_CODE` to generate a **fasta-style alignment** of one chromosome using the following example.

Ex. 24. `./convertSFS_CODE out.txt -a I 1 0`

This would print the alignment of three chromosomes to the screen. It might look like the following:

```
>it0pop0ind0locus0
GTATGTATAGGGCTTGGTATTGAAAATAGGTCCCAGGAAATCTTGACCGGCACCCAAGAGGTCATG
>it0pop1ind0locus0
GTATGTATAGGGCTTTATATTGAAAATAGGTCCCAGGAAATCTTGACCGGCACCCAAGAGGTCATG
>it0pop2ind0locus0
GTATGTATAGGGCTTTATATTGAAAATAGGTCCCAGGAAATCTTGACCGGCACCAAAGAGGTCATG
```

The name of each sequence indicates the iteration ("`it0`"), the population ("`pop0`"), the individual chromosome ("`ind0`"), and the locus ("`locus0`"), followed by the actual sequence on the next line. If you just wanted to extract a single sequence from human and chimpanzee, then you would use the "`P.I`" option, which would allow you to select certain chromosomes from certain populations. In this case, you would use "`P.I 2 0.0 1.0`" to indicate that you want two chromosomes to be printed: chromosome 0 from population 0 and chromosome 0 from population 1.

Ex. 25. `./convertSFS_CODE out.txt -a P.I 2 0.0 1.0`

If you also want to include the true ancestral sequence of each population to be printed, include the character '`A`'. Note that this is the ancestral sequence of a population, and not the ancestral sequence of a pair or group of populations. It will be printed just as any other sequence from a population, but will have "`indA`", such that the chromosome identifier is '`A`'.

To generate the **McDonald-Kreitman table** for the human-chimp simulation, you would use the following example.

Ex. 26. `./convertSFS_CODE out.txt --MK 1 0`

This indicates that you want to use population 1 for polymorphism (the human population) and population 0 as an outgroup to call fixed differences (chimp polymorphism is not included). The output for this particular case is as follows: `0 0 0 2 1.0000`. Not very interesting. There were (in order of appearance) zero synonymous and nonsynonymous polymorphisms, zero synonymous fixed differences, and two nonsynonymous fixed differences. The last value given is the Fisher exact test p-value for the table. You can print the table to a file using the flag "`F <filename>`". If you ran several iterations, you can print

each one independently using "`ITS -1`". More generally, if you just want to print the first n iterations, replace "-1" with "n", the -1 just allows you to not worry about the actual number of simulations that were done. By default, the outgroup sample size is 1 (*e.g.*using just the reference sequence to call fixed differences), but this can be changed to n using the option "`OGSS` n". This is helpful if you want to test the effect of using multiple outgroup sequences on calling fixed differences (many sites that are apparently fixed between populations are actually the result of sampling a common mutation segregating in one of the populations). Finally, if you want to use parsimony to call synonymous and nonsynonymous mutations (instead of using their true classification stored during the mutation), use "`OBS`". As an example, suppose that the file `out.txt` contained several simulations from two populations across 3 loci, but we wanted the observed (i.e. using parsimony) MK table for the first locus using a sample size of 2 for the outgroup. We could use the following example:

Ex. 27. `./convertSFS_CODE out.txt --MK 1 0 F mk.txt ITS -1 L 1 0 OGSS 2 OBS`

For printing the **site-frequency spectrum** (**SFS**), use `--SFS`. There are many similar options for this output. However, you can also specify the SFS of just autosomal (`A`), X or Y linked SNPs (`X` or `Y`, respectively). You can also specify the type of mutations to include in the SFS (*e.g.*synonymous, nonsynonymous, or both). In this case, you would use "`T 0`", "`T 1`", or "`T 2`" (respectively). Non-coding mutations are considered synonymous, and will not be reported for "`T 2`". Sometimes using an outgroup to identify the ancestral state of a polymorphism under parsimony will be wrong (Hernandez et al., 2007b). By default, the true SFS will be generated, but you can also use parsimony (if you've simulated at least two populations) by including "`OBS <ing> <og> [og`$_{\texttt{size}}$`]`", where `ing` is the in-group used for polymorphism, `og` is the outgroup used for identifying the ancestral states of polymorphisms, and the optional argument `og`$_{\texttt{size}}$ indicates the number of sequences to use from the outgroup (default is 1).

To produce output in a similar format as the coalescent simulator **ms** (Hudson, 2002), you can use the option `--ms`. You can print the output to a file using `F <file>`, and specify the type of mutations to output (either synonymous/non-coding, `T 0`; nonsynonymous, `T 1`; or both `T 2`).

You can produce the input file for the Bayesian clustering algorithm **STRUCTURE** (Falush et al., 2003) using the option `--structure`. For this option, you can specify the centimorgans per megabase (using `CMMB <c>`), and restrict the output to either specific individuals (`I <n> ...`), specific populations (`P <n> ...`) or specific individuals from specific populations (`P.I. <n> ...`).

When simulating many populations, you may want a table showing the number of private and shared polymorphisms. This can be generated using the `--privateShared` option, which will sum the number of private and shared polymorphisms within and between two populations. This option has common flags such as `F <filename>` for printing the output to a file, `ITS <n>` for printing just the first `n` iterations (or all iterations independently if `n`=1; default is to sum cell entries over all simulations in the file), and `T <t>` for setting the type of mutations (*e.g.*`t`=0 for synonymous/non-coding, `t`=1 for nonsynonymous, and `t`=2 for all mutations). If you simulated more than 2 populations, you can specify which pairwise comparison you'd like to evaluate using `P <`p_1`> <`p_2`>`. This will output 3 entries: the number of polymorphisms private to the first population, the number of polymorphisms private to

the second population, and then the number of polymorphisms shared between populations.

Since `SFS_CODE` keeps track of the time each mutation arose and fixed (if did), it is possible to analyze the distribution of ages of mutations in terms of population scaled times using the option `--AGE`. This option will print a table with 5 columns for each variant: the iteration, the locus, the site, the fitness effect, and the population scaled age. This command allows the following options: `F [a] <filename>` to print the output to a file, `ITS <n>` to print a subset of `n` iterations, `P <pop>` to indicate a specific population, and `T <type>` to specify a mutant type (0 for synonymous/non-coding, 1 for non-synonymous, and 2 for all mutations).

Each individuals carries some number of derived alleles. This can be extracted and analyzed using the command `--derivedAlleles`. This command will print out a table with each iteration on a different line, each population tab delimited, and within each population each individual (corresponding to 2 chromosomes in a diploid population) space delimited. Each entry corresponds to the number of derived alleles carried by the given individual (*e.g.*in a diploid population, homozygous derived genotypes carry 2 alleles while heterozygous genotypes carry a single allele). You can print the output to a file using `F [a] <filename>`, extract only the first `n` iterations using `ITS <n>`, or specify a specific type of mutation using `T <t>`. You can also count the number of heterozygous/homozygous genotypes carried by each individual using the option `H <h>`, where `h`=0 indicates only heterozygous sites, `h`=1 indicates only homozygous sites, and `h`=2 (the default) counts both heterozygous and homozygous sites.

You can also analyze the fitness effects of mutations subject to a given criteria. In particular, using the command `--fitness` you can print out a table showing the fitness effect of mutations that are fixed `f`, private to a given population (or set thereof) `p`, or shared between populations `S <npops>` <p_1>...<p_{npops}>. The output can be sent to a file `F [a] <filename>`, restricted to a set of iteration `ITS <n>`, or restricted to a single population `P <pop>`.

It is important to note that you can use several options at once. For example, if you want to generate both MK tables and the SFS, you can put them both in the command line. Additionally, if you simulated several species, and want to generate the observed SFS using species with increasing divergence, then you can just concatenate all your `--SFS` commands as follows:

Ex. 28.
```
./convertSFS_CODE out.txt --SFS OBS 0 1 F sfs1.txt \
  --SFS OBS 0 2 F sfs2.txt --SFS OBS 0 3 F sfs3.txt
```

Finally, to reiterate a word of caution mentioned at the end of the last section, when simulating multiple populations, mutations that have fixed independently in two daughter lineages will be printed multiple times in the output file. Not all of the commands implemented in `convertSFS_CODE`are robust to this at this time.

Table 4: `convertSFS_CODE` Options.

long	short	<arguments>	description
--help	-h	∅	**Display help menu**
--alignment	-a	**Print sequence alignment in fasta format**	
		[A]	Print ancestral population sequence
		[F <file>]	Print sequences to a file
		[L <n> <L_1>...<L_n>]	Only print n loci.
		[I <n> <I_1>...<I_n>]	Only print n chromosomes (but from all populations).
		[P <n> <P_1>...<P_n>]	Only print specific populations.
		[P.I <n> <p_1.c_1>...]	Only print specific chromosomes from specific populations
		[ITS <n>]	Only print first n iterations
--ms	-m	**Produce ms-style output**	
		[F <file>]	Print MK tables to a file
		[T <type>]	Extract mutations of <type> = 0, 1, or 2 (synon., nonsynon., or both)
--MK	-M	**Print McDonald-Kreitman tables**	
		<ing> <og>	Ingroup and outgroup (**required**)
		[F <file>]	Print MK tables to a file
		[ITS [n]]	Print each iteration [or just first n]
		[L <n> <L_1>...<L_n>]	Only print n loci.
		[OGSS <size>]	Set the outgroup sample size
		[N <n>]	Randomly sample n chromosomes from each population
		[OBS]	Use parsimony to call synonymous and nonsynonymous mutations
--structure	-s	**Print structure input**	
		[F <file>]	Print to a file
		[CMMB <c>]	set centiMorgans/Megabase to c
		[I <n> <I_1>...<I_n>]	Only print n chromosomes (but from all populations).
		[P <n> <P_1>...<P_n>]	Only output specific populations.
		[P.I <n> <p_1.c_1>...]	Only print specific chromosomes from specific populations
--SFS	-S	**Print site-frequency spectra (SFS)**	
		[A]	Extract only autosomal loci
		[F <file>]	Print SFS to a file
		[ITS [n]]	Print each iteration [or just first n]
		[L <n> <L_1>...<L_n>]	Only print n loci.
		[N <n>]	Randomly sample n chromosomes from each population
		[OBS <ing> <og> [og_{size}]]	Use parsimony to identify ancestral alleles from an outgroup [using og_{size} chromosomes]
		[I <n> <I_1>...<I_n>]	Only print n chromosomes (but from all populations).
		[P <n> <P_1>...<P_n>]	Only output specific populations.
		[T <type>]	Extract mutations of <type> = 0, 1, or 2 (synon., nonsynon., or both)
		[X]	Extract only X-linked mutations
		[Y]	Extract only Y-linked mutations

10 A Complicated Example for Converting Between ms and SFS_CODE Command-lines

This section is really for the ms aficionados out there. If you are just learning how to use ms, then previous examples may be sufficient to convert basic commands between the two programs (*e.g.*, Figure 2 on page 15).

The coalescent simulator ms operates backward in time, with the time of sampling considered time 0, and increasing as you go back in time to the common ancestor. SFS_CODE operates forward in time, with the time 0 representing the end of the burn-in, and increasing until the time of sampling. Another complication is that ms scales time by $4 \times N_0$ generations (where N_0 refers to a population of your choice, depending on how you code it), and SFS_CODE scales times by $P \times N_A$ (where P is the ploidy, and N_A is always the ancestral population).

Here is an extremely complicated example of simulating a Mexican-American population from Gutenkunst et al. (2009). In order to follow this description, you will need to consult Figure 3b from that paper.

Description of the model. There are a total of 4 populations in this model: 0=Africa (YRI), 1=Europe (CEU), 2=Chinese (CHB), and 3=Mexican American (MXL). There is a historical expansion, followed by an out-of-Africa bottleneck with migration between Africa and the Eurasian population. Then there are European and Asian specific bottlenecks after they split, followed by exponential growth with migration. The Native American population split off from the Asian population, and was isolated until the last generation at which point the Mexican American population is formed by admixture between Native Americans and Europeans.

To sample 120 chromosomes from the MXL population requires a LONG command-line. In ms, you will need to note that the way admixture works is that a dummy population is created, which must be merged back with the true ancestral population. The command-line looks like the following:

```
./ms 120 1 -t 1 -I 4 0 0 0 120 -n 1 1.682020 -n 2 2.424020 -n 3 4.185850 \
-n 4 7.942130 -es 0 4 0.522451 -ej 0 5 2 -eg 0 2 67.978337 -eg 0 3 109.406463
\
-eg 0 4 147.474095 -ema 0 5 x 0 0 0 x 0 x 3.960400 0 x 0 3.960400 x 0 x 0 0 0
\
x x x x x x x -ej 0.029475 4 3 -ema 0.029475 5 x 0.881098 0.561966 x x 0.881098
x \
3.960400 x x 0.561966 3.960400 x x x x x x x x x x x x x x -ej 0.036114 3 2 \
-en 0.036114 2 0.287184 -ema 0.036114 5 x 7.293140 x x x 7.293140 \
x x x x x x x x x x x x x x x x x x x x x -ej 0.197963 2 1 -en 0.303500 1 1
```

Unfortunately, in SFS_CODE, the command is no simpler... It has the following form:

```
./sfs_code 5 1 -n 0 0 0 0 60 -Td 0 P 0 1.68202 -TS 0.211074 0 1 \
-Td 0.211074 P 1 0.170737565 -TS 0.534772 1 2 -Td 0.534772 P 1 0.724763218 \
-Td 0.534772 P 2 0.162342748 -Tg 0.534772 P 1 33.98917 -Tg 0.534772 P 2 62.26654
\
-TS 0.54805 2 3 -Td 0.54805 P 3 0.294502108 -Tg 0.54805 P 3 93.86859 -TE 0.607
```

```
\
-TJ 0.607 4 -3 2 3 1 0.522451 0.477549 -Tm 0.211074 P 0 1 6.133603671 \
-Tm 0.211074 P 1 0 1.047236559 -Tm 0.534772 P 0 1 0.741012229 \
-Tm 0.534772 P 0 2 0.472619025 -Tm 0.534772 P 1 0 0.091696045 \
-Tm 0.534772 P 1 2 0.41215962 -Tm 0.534772 P 2 0 0.013100056 \
-Tm 0.534772 P 2 1 0.092321359 -Tm 0.54805 P 0 1 0 -Tm 0.54805 P 0 2 0 \
-Tm 0.54805 P 1 0 0 -Tm 0.54805 P 2 0 0
```

Now lets work through this step-by-step. Because this command-line is so complicated, converting these two commands should address most issues that will arise. First, note that the way this `ms` command-line is structured, N_0 refers to the ancestral population. That helps us a lot. If it were not the case, then all the timed events would require an additional factor. Now let's talk about the timed events (expansions, bottlenecks, splitting).

Let's look at this forward in time. The ancestral expansion in `ms` occurs at time 0.3035 (`-en 0.303500 1 1`). This will be the first event to occur for `SFS_CODE`, and will represent time zero. Given that `ms` scales time by $4N$ generations and `SFS_CODE` scales time by $2N$ generations, scaling is off by a factor of 2, so we immediately we know that we are going to end the `SFS_CODE` simulation at twice the `ms` time: 0.607 (`-TE 0.607`). The size of the ancestral expansion can be determined from the current size of the African population: `-Td 0 P o 1.68202`. In `ms`, the out-of-Africa event occurs at time 0.197963 (`-ej 0.197963 2 1`). Looking at this forward in time, we have $2 \times (0.305 - 0.197963) = 0.211074$ as the time parameter for `SFS_CODE`: `-TS 0.211074 0 1`.

European and Chinese populations split at time 0.036114 in `ms`, so in `SFS_CODE`, the time parameter is $2 \times (0.3035 - 0.036114) = 0.534772$, which gives: `-TS 0.534772 1 2`. The Native Americans split from Chinese at time 0.029475 in the `ms` command, so the time parameter in `SFS_CODE` is $2 \times (0.3035 - 0.029475) = 0.54805$ which gives: `-TS 0.54805 2 3`.

For the demographic effects, `ms` inputs the relative some compared to N_0, while `SFS_CODE` uses the relative size change at the time of the event. These are slightly more tricky to keep track of, since you have to determine the relative size at each time point. The Eurasian bottleneck in `ms` is coded as `-en 0.036114 2 0.287184`. Note that this represents the end of the bottleneck going forward in time, and that it actually starts at the time of the split (join in `ms`) from Africa at `SFS_CODE` time 0.211074. The population becomes $0.287 \times N_A$, but it occurs after the expansion, so in `SFS_CODE` the magnitude of the change is actually 0.287184/1.68202=0.170737565, which gives `-Td 0.211074 P 1 0.170737565`.

The European and Chinese bottlenecks are a bit trickier yet. These population sizes are not explicitly stated in the `ms` command-line, since there is exponential growth. However, given that the final size of the European population (population 2 in `ms`, population 1 in `SFS_CODE`) in `ms` is $2.42402N_A$ and the growth rate was 67.978337, we can back-calculate what the population size was at the time of the bottleneck using the following equation: $N_B = N_F \times \exp(-\alpha t) = 2.4202 \times \exp(-67.978337 \times 0.036114) = 0.2081404 \times N_A$. Analogously, the size of the Chinese bottleneck was $0.04662224 \times N_A$. The magnitude of the European bottleneck was then 0.2081404/0.287184 = 0.724763218, and the size of the Chinese bottleneck was 0.04662224/0.287184 = 0.162342748. The options for the events were then `-Td 0.534772 P 1 0.724763218 -TD 0.534772 P 2 0.162342748`.

The exponential growth rates in `ms` are different from what they should be in `SFS_CODE`

because of the scaling issue, and growth being determined by the current size in SFS_CODE rather than the ancestral size in ms. you can calculate the growth parameter using the relative effective sizes at the beginning and end of growth: $\alpha_{\texttt{SFS\_CODE}} = \log(N_F/N_B)/t$. For the European population, this is then $\alpha_E = \log(2.42/0.2081404)/0.072228 = 33.98917$. This is set using the flag -Tg 0.534772 P 1 33.9817. The other grown rates are analogous.

The migration rates are yet even trickier. Both SFS_CODE and ms use a population scaled migration rate. However, SFS_CODE uses 2 times the current effective population size (holding the underling rate constant), whereas ms uses $4N_0$, and updates the rate as the population size changes. Again, we are off by a factor of 2 and the relative effective population sizes. So, $m_{\text{SFS\_CODE}} = m_{\text{ms}} \times N_B/2$. For the migration rate corresponding to CHB into CEU, we have $3.9604 \times 0.2081404/2 = 0.41215962$. The other migration rates are analogous and relatively straight forward once you know the population size at each transition.

The last remaining component is admixture. In SFS_CODE, admixture results in creating a new population, so MXL is actually population 3 in the SFS_CODE command above. Since the admixture event occurs in the last generation, the command is fairly straightforward to put together if you've made it this far. The time is just the time of sampling (0.607), the population size is just going to be the size of population 3 (the Native Americans), and the proportions of european and Native American contributions are exactly the same as in the ms code, 0.522451 from Europe and the rest from Native Americans.

11 Examples

This section will include additional examples to demonstrate usage of SFS_CODE. Over time, this section will grow to include relevant demographic models and distributions of selection coefficients from the literature.

11.1 Recurrent Hitchhiking

Simulating recurrent hitchhiking (RHH) in a forward population genetic framework is a bit involved. Say we are interested in a population of, $N = 5 \times 10^3$ with substitution rate $\lambda = 8 \times 10^{-8}$ per base pair per generation, $\alpha = 2Ns = 10^3$, $\rho = 8 \times 10^{-3}$, and a neutral mutation rate of $\theta_n = 2 \times 10^{-3}$. We include a neutral locus of length$L_n = 10^3$ bp that is flanked by loci on either side that are subject to selection and are each of length $L_f = 10^5$ bp.

In a forward simulation, you can only specify the mutation rate, not the substitution rate. So, for a given substitution rate λ, we must divide by the fixation probability to obtain the corresponding mutation rate of selected mutations. Let ν be the per base pair mutation rate in the flanking loci:

$$\nu = \frac{\lambda}{P_{fix}} = \frac{\lambda(1 - e^{-2\alpha})}{(1 - e^{-2s})} = 4.413 \times 10^{-9} \tag{3}$$

In SFS_CODE, we can only specify a single value of θ. The mean number of mutations that

enter the population each generation would then be $\theta \sum L_i/2$. However, the mean number of mutations we want entering the population in the neutral locus is $L_n\theta_n/2$, and the mean number of mutations entering each flanking locus is $L_f\nu$. Setting the sum of these two terms equal to $\theta \sum L_i/2$ and solving for θ gives us our overall θ to use in the simulation:

$$\theta = \frac{L_n\theta_n + 4L_f\nu}{L_n + 2L_f} = 9.959 \times 10^{-6} \tag{4}$$

Lastly, we specify the rates of mutation that enter the flanking and neutral loci using the option `-v` described on page 25: `-v L 0` $L_f\nu$ `-v L 1` $L_n\theta_n$ `-v L 2` $L_f\nu$. The command line supplied to `SFS_CODE` is then:

Ex. 29. `sfs_code 1 1 -t 9.959e-06 -Z -r 0.008 -N 5000 -n 10 -L 3 1e5 1e3 1e5 \`
`-a N R -v L 0 4.41e-4 -v L 1 2.0 -v L 2 4.41e-4 -W L 0 1 1e3 1 0 -W L 2 1 1e3 1 0`

12 Default Parameter Values

Running `SFS_CODE` with the default parameters will generate a sample of six diploid individuals (12 chromosomes) under the standard neutral model assumptions of a constant population size, absence of selection, etc. Every parameter discussed below can be changed using the command line options described in Table 6. The full list of default parameter values are given in Table 5.

13 Summary of Options and Arguments

In `SFS_CODE`, an **option** is a feature that allows you to change the characteristics of the simulation. Every option implemented in `SFS_CODE` is summarized in table 6. There are basically five types of options: (1) those that control the output, (2) those those that affect all populations or set foundation for the simulation ("Global Options"), (3) those that may be population specific ("Population Options"), (4) those that describe the effect of natural selection, and (5) those that govern the demographic events ("Evolutionary Events"). All options (except evolutionary events) have both a **long name** (preceded by two dashes) and a **short name** (a single character preceded by a single dash), which can be used interchangeably (*e.g.*you could either use "`--theta 0.01`" or "`-t 0.01`" to set the population scaled mutation rate to 0.01 for all populations). Most options have **arguments**. Arguments that are **required** are enclosed in `<angled brackets>`. Arguments that are **optional** are enclosed in `[square brackets]`.

Some options apply to all populations (*e.g.*the length of the simulated sequence `-L`), and some apply only to a specific population (such as the generation effect `-G`). Others default to all populations, but allow you to specify a specific population. These are denoted by `[P <pop>]` in the argument list. This means that you can add the character '`P`' followed by the number of a specific population to apply the option to that population exclusively.

Table 5: Default parameter values used in SFS_CODE.

**Parameter**	**value**
Effective population size	500
Ploidy	2
Allo- (0) or Auto-ploidy (1)	0
Relative size of female population	0.5
Sample size for each population	6
Migration between populations	0.0
Probability male migrates (if migration)	0.5
θ/site	0.001
ρ/site	0
Substitution model	0
Number of mutation rate classes (sites)	1
Number of mutation rate classes (loci)	1
Infinite sites?	no
ρ/site	0.0
Number of loci	1
Length of locus (bp)	5000
Linkage within loci	0.0
Annotation	`C`
Sex chromosome?	no
Self-fertilization rate	0.0
Selection distribution	0
Multiplicative fitness	yes
Proportion of loci subject to selection	1.0
Non-lethal mutation rate	1.0
Initial burn-in period ($\times$PN)	5
Burn-in period of subsequent iterations	2
Print ancestral sequence?	yes

For example, if you are simulating two populations, one of which is twice the size of the other, you might add "`-N P 1 1000`" to your command line.

Generally speaking, options can be used in any order. The exceptions are `--linkage`, `--annotate`, and `--sex`, which must come after `-L` if `-L` is used (if `-L` is not used, then order really doesn't matter). However, arguments for each option are in a specified order, and must always be used in the proper order.

Table 6: SFS_CODE Options

	long	short[a]	<arguments>	description
**Output**	--help	-h	$\emptyset$	Display help menu. (p6)
	--noSeq	-A	$\emptyset$	DO NOT print ancestral sequences. (p41)
	--outfile	-o	[a] <file>	Write [or <a>ppend] output to a <file> instead of the screen. (p41)
	--errfile	-e	[a] <file>	Write [or <a>ppend] error messages to a <file>. (p41)
	--popFreq	-F	[a] <file>	Write [or <a>ppend] sample/population frequencies to a <file>. (p43)
	--ReportBurnFixed	-R	$\emptyset$	Report mutations that fixed during the burn-in period with the rest of the output. (p40)
**Selection**	--additive	-Z	$\emptyset$	Use an additive model of selection. (p15)
	--selDistType	-W*	[P/L/F...] <type> [arg]	Distribution of selective effects. (p16, Table 1)
	--neutPop	-w*	<pop>	No selection on population <pop>. (p18)
	--constraint	-c*	[P/L] <f$_0$>	Set the proportion of non-lethal mutations to <f$_0$>. (p19)
**Global Options**	--BURN	-B	<burn>	Burn-in time for initial population. (p8)
	--BURN2	-b	<burn>	Burn-in time for subsequent simulations. (p8)
	--length	-L	<R> <L$_1$> [<L$_2$>..] [R]	Simulate <R> loci (regions) with lengths <L$_1$> [<L$_2$>... opt.; add 'R' after a subset of lengths to repeat]. (p8)
	--linkage	-l	<p/g> <d$_1$> [<d$_2$>..] [R]	Set linkage between adjacent loci (either <p>hysical, or <g>enetic distance) to <d$_1$> [<d$_2$> ..<d$_{R-1}$> opt.; add 'R' to repeat pattern]. MUST USE AFTER -L. (p9)
	--annotate	-a	<a$_1$> [<a$_2$>..<a$_R$>] [R]	Annotate each locus as C (coding) or N (non-coding) [<a$_2$>..<a$_R$> opt; add 'R' to repeat pattern]. Note: If coding (default), length will be rounded to nearest codon. (p10)
	--sex	-x	<x$_1$> [<x$_2$>...] [R]	Annotate sex loci, either 0 or 1 (autosomal or sex, resp.). Use only <x$_1$>, or specify each locus, or put 'R' at end to repeat a subset. Only implemented for the diploid case (P=2). Males do not recombine at sex loci. (p10)
	--ploidy	-P	<ploidy>	Set the ploidy of all populations to <ploidy> (1,2,4 only; i.e. haploid, diploid, or tetraploid). (p12)
	--tetraType	-p	<0/1>	If P=4 (tetraploid) assume auto- or allotetraploid (0 or 1, resp.). (p13)
	--substMod	-M	<mod> [args]	Set the mutation model. (p23, Table 2.)
	--INF_SITES	-I	$\emptyset$	Avoid multiple mutations segregating at the same site concurrently. Multiple hits can still occur for long divergence times. (p25)
	--seed	-s	<int>	Set random number seed to <int>. This should always be set manually if using this program on a cluster!! (p38)

Continued on next page...

Table 6 – continued from previous page

	long	short[a]	<arguments>	description
**Population Options**	--theta	-t*	[P <p>] <θ>	Set the PER SITE population scaled mutation rate to <θ> for ALL populations [or just population <p>]. (p7)
	--rho	-r*	[P <p>] <ρ>	Set the PER SITE population scaled recombination rate to <ρ> for ALL populations [or just population <p>]. (p7)
	--pMaleRec	-Y*	[P <pop>] <p>	Set the proportion of recombinants originating in the paternal lineage. (p8)
	--geneConversion	-H*	[P <pop>] [B <B_{GC}>] <f> <λ>	Set the parameters for gene conversion. (p27)
	--indel	-u*	[P <p>] <INS> <DEL> <LEN>	Set the PER SITE population scaled indel rates and lengths for ALL populations [or just population <p>]. (p31)
	--longIndel	-U*	[P <p>] <INS> <DEL> <LEN>	Set the PER SITE population scaled indel rates and lengths for ALL populations [or just population <p>]. (p31)
	--inversion	-z*	[P <p>] <INV> <LEN>	Set the PER SITE population scaled inversion rate and lengths for ALL populations [or just population <p>]. (p32)
	--popSize	-N*	[P <p>] <size>	Set all population to size <size> 'P'-ploid individuals [or just population <p>]. (p11)
	--sampSize	-n	P/R/S/<SS$_1$>[..<SS$_{NPOP}$>]	Sample <SS$_1$> individuals from population 1, ..., <SS$_{NPOP}$> individuals from population NPOP. Use the value -1 to sample an entire population. (p??)
	--admixture	-TJ	<τ> <P> <N> <N_{anc}> <P_1>..<P_N> <M_1>..<M_N> [F <F_1>..<F_N>]	Create admixed population P of size N from N_{anc} populations with proportions M_i from population P_i, and optionally different female proportions. (p22)
	--migMat	-m*	A <M>	Set the migration rate to/from all pops to <M>/(NPOP-1). (p21)
			P <P$_{to}$> <P$_{from}$> <M>	Set the migration rate entry $m_{to,from}$ = <M>
			L <M$_{0,1}$>...<M$_{NPOP,NPOP-1}$>	Set all entries of the migration matrix.
	--pMaleMig	-y*	[P <p>] <p$_{male}$>	Set the proportion of migrants out of each population [or just population <p>] that are male to <p$_{male}$>. (p22)
	--propFemale	-f	[P <p>] <pf>	Set the proportion of females in each population [or just <pop>] to <pf>, default 0.5. (p22)
	--self	-i*	[P <p>] <s>	Set the selfing rate <s> for ALL populations [or just population <p>]. (p32)
	--KAPPA	-K*	[P <p>] <κ>	Set transition/ transversion rate ratio <κ> (only valid for --substMod 2 or 3). (p24)
	--PSI	-C*	[P <p>] <ψ>	Set CpG bias parameter (non-CpG rejection rate; only valid for --substMod 1 or 3). (p24)
	--baseFreq	-q*	[P <p>] <π_C> <π_G> <π_T> <π_A>	Set equilibrium nucleotide frequencies. (p23)
	--rateClassSites	-V	[P <p>] <n$_{classes}$> <α>	Mutation rate variation among sites in loci (discrete Gamma model of <n$_{classes}$> classes with rate <α> and mean 1). (p25)
	--rateClassLoci	-v	[L <l/r>] [[P <p>] <n_c> <α>]	Mutation rate variation among loci (discrete Gamma model of <n$_{classes}$> classes with rate <α> and mean 1). (p25)

Continued on next page...

Table 6 – continued from previous page

	long	short[a]	<arguments>	description
	--GenEffect	-G*	<pop> <G>	Generation time effect. <G> > 1 makes <pop> experience <G> rounds of mating each generation while if <G> < -1 mating occurs only every |<G>| generations. <G> must be an integer, with <G> > 0 or <G> < -1. (p33)
**Evolutionary Events**		-Td	<τ> [P <p>] <ν>	Discrete population size change at time <τ> with magnitude <ν> $= \mathtt{N_{new}}/\mathtt{N_{old}}$, where $\mathtt{N_{old}}$ is the size of <pop> prior to the event, NOT ANCESTRAL! (p11)
		-Tg	<τ> [P <p>] <α>	Set the exponential growth rate of a population to <α> at time <τ>. (p12)
		-Tk	<τ> [P <p>] <K> <r>	Logistic growth at rate <r> beginning at time <τ> until final population size <K> is reached. (p12)
		-TE	<τ> [p]	Terminate simulation [or just a population] at time <τ> $\times PN0$. (p13)
		-TS	<τ> <i> <j>	Split population <i> at time <τ>$\times PN$ generations to found population <j>. (p19)
		-TD	<τ> <i> <j> <f> <N> [l]	Domesticate population <j> with <N> individuals from <i> at time <τ> using a derived allele at frequency <f>$\pm 5\%$. (p20)
**Other**	--mutation		<τ> [P/L] [S <s>] [G <γ>]	Introduce a mutation at a specified time, place and fitness. (p26)
	--printGen			Print each generation throughout the simulation. (p41)
	--trackAncestry		[a] <file>	Print true ancestry of each position and chromosome. (p45)
	--trackTrajectory		[T/P/L/S/R.../F]	Track frequency trajectory. (p43)

[a] Asterisk in short name indicates that the parameters can be changed (or option initiated) at any time using -T<short_name> <τ> <args>. See Section 4.9 on page 33.

References

Boyko, A. R., Williamson, S. H., Indap, A. R., Degenhardt, J. D., Hernandez, R. D., Lohmueller, K. E., Adams, M. D., Schmidt, S., Sninsky, J. J., Sunyaev, S. R., White, T. J., Nielsen, R., Clark, A. G., and Bustamante, C. D. (2008). Assessing the evolutionary impact of amino acid mutations in the human genome. *PLoS Genet*, 4(5):e1000083.

Caicedo, A. L., Williamson, S. H., Hernandez, R. D., Boyko, A., Fledel-Alon, A., York, T. L., Polato, N. R., Olsen, K. M., Nielsen, R., McCouch, S. R., Bustamante, C. D., and Purugganan, M. D. (2007). Genome-wide patterns of nucleotide polymorphism in domesticated rice. *PLoS Genetics*, 3(9):1745–1756.

Cutler, D. J. (2000). Understanding the overdispersed molecular clock. *Genetics*, 154(3):1403–1417.

Falush, D., Stephens, M., and Pritchard, J. K. (2003). Inference of population structure using multilocus genotype data: linked loci and correlated allele frequencies. *Genetics*, 164(4):1567–1587.

Felsenstein, J. (1981). Evolutionary trees from DNA sequences: a maximum likelihood approach. *J Mol Evol*, 17(6):368–376.

Gutenkunst, R. N., Hernandez, R. D., Williamson, S. H., and Bustamante, C. D. (2009). Inferring the joint demographic history of multiple populations from multidimensional snp frequency data. *PLoS genetics*, 5(10):e1000695.

Hasegawa, M., Kishino, H., and Yano, T. (1985). Dating of the human-ape splitting by a molecular clock of mitochondrial dna. *J Mol Evol*, 22(2):160–174.

Hernandez, R. D., Hubisz, M. J., Wheeler, D. A., Smith, D. G., Ferguson, B., Rogers, J., Nazareth, L., Indap, A., Bourquin, T., McPherson, J., Muzny, D., R., Nielsen, R., and Bustamante, C. D. (2007a). Demographic histories and patterns of linkage disequilibrium in Chinese and Indian rhesus macaques. *Science*, 316(5822):240–243.

Hernandez, R. D., Williamson, S. H., and Bustamante, C. D. (2007b). Context dependence, ancestral misidentification, and spurious signatures of natural selection. *Mol Biol Evol*, 24(8):1792–1800.

Hudson, R. R. (2002). Generating samples under a Wright-Fisher neutral model of genetic variation. *Bioinformatics*, 18(2):337–338.

Hwang, D. G. and Green, P. (2004). Bayesian Markov chain Monte Carlo sequence analysis reveals varying neutral substitution patterns in mammalian evolution. *Proc Natl Acad Sci U S A*, 101(39):13994–14001.

Jukes, T. H. and Cantor, C. R. (1969). Evolution of protein molecules. In Munro, H. N., editor, *Mammalian protein metabolism*, pages 21–132. Academic Press, New York.

Kimura, M. (1980). A simple method for estimating evolutionary rates of base substitutions through comparative studies of nucleotide sequences. *J Mol Evol*, 16(2):111–120.

Tavaré, S. (1986). Some probabilistic and statistical problems in the analysis of dna sequences. *American Mathematical Society: Lectures on Mathematics in the Life Sciences*, 17:57–86.

Uricchio, L. H. and Hernandez, R. D. (2014). Robust forward simulations of recurrent hitchhiking. *Genetics*, 197(1):221–236.

Zhang, Z. and Gerstein, M. (2003). Patterns of nucleotide substitution, insertion and deletion in the human genome inferred from pseudogenes. *Nucleic Acids Res*, 31(18):5338–5348.
