# Supplementary material for: Human demographic history has amplified the effects of background selection across the genome
Source: PLoS Genet. 2018 Jun 18;14(6):e1007387. doi: 10.1371/journal.pgen.1007387 (PMC6056204; doi:10.1371/journal.pgen.1007387)
Supplement: S1 Appendix — (PDF) [file pgen.1007387.s002.pdf]

S1 Appendix. Soft sweep detection and implementation in selscan v1.2.0.

Detecting soft sweeps

Under the model of a soft sweep, there is an increased chance of multiple distinct haplotypes sweeping to high frequency in a population. Garud et al. [1] developed a window-based statistic ($H12$) with good power to detect this process, and here we adapt $H12$ into an integrated haplotype homozygosity framework [2–4]. We call this new statistic $iHH12$. The general principle of these statistics is to combine the top two most frequent haplotypes into a single haplotype class to avoid the reduced power that iHS has when the adaptive allele segregates on more than one haplotype background. We calculate $iHH12$ as follows.

Following the notation of Szpiech and Hernandez [5] in a sample of n chromosomes we let $\mathcal{C}$ be the set of all possible distinct haplotypes at the locus x_0. $\mathcal{C}(x_i)$ is then the set of all possible distinct haplotypes extending from locus x_0 to locus x_i. Let h_i in $\mathcal{C}(x)$ be the ith most frequent haplotype. We then calculate $EHH12$ of the entire sample of haplotypes from x_0 to x_i as

$$EHH12(x_i) = \frac{\binom{n_{h_1}+n_{h_2}}{2}}{\binom{n}{2}} + \sum_{j>2}^{|\mathcal{C}(x_i)|} \frac{\binom{n_{h_j}}{2}}{\binom{n}{2}}$$

where n_{h_j} is the number of h_j haplotypes in the sample.

If $EHH12(x_i)$ is calculated repeatedly for several $x_i$moving farther away from x_0, we expect to observe more haplotypes and therefore we expect to observe lower haplotype homozygosity. However, the decay of homozygosity is slower in a region under selection [2–4]. Therefore, we integrate the decay of $EHH12$ as a function of genetic distance in order to summarize the pattern and make genome-wide comparisons. This integrated score is calculated as

$$iHH12 = \sum_{i=1}^{|\mathcal{D}|} \frac{1}{2}(EHH12(x_{i-1}) - EHH(x_i))g(x_{i-1}, x_i) + \sum_{i=1}^{|\mathcal{U}|} \frac{1}{2}(EHH12(x_{i-1}) - EHH(x_i))g(x_{i-1}, x_i)$$

where $g(x_{i-1}, x_i)$ is the genetic distance between markers x_{i-1} and x_i . $\mathcal{D}$ and $\mathcal{U}$ represent sets of markers downstream and upstream from x_0, respectively. In practice, the curve is integrated until $EHH12 < 0.05$ on both sides of the focal locus. Finally, $iHH12$ is normalized genome-wide in order to account for the effects of demographic history on the distribution of haplotype homozygosity. We integrated this new statistical framework to detect soft-sweeps into `selscan` version 1.2.0 (https://github.com/szpiech/selscan) [5].

We evaluated the power of our $iHH12$ statistic implementation in `selscan` to detect hard and soft sweeps relative to iHS across a range of parameters. We simulated neutrally evolving sequences with `ms` [6] and non-neutrally evolving sequences with `mssel`, a modified version of `ms` also developed by R.R. Hudson that conditions on an allele frequency trajectory. We simulated trajectories backwards in

time under a selection on standing variation model with $s = 0.01$. Once an adaptive variant reached a set frequency backwards in time, the selection coefficient was set to $s = 0$ and was allowed drift neutrally until loss. We simulated 200 replicates across several sampling frequencies (0.7, 0.8, 0.9), several frequencies at which the variant become adaptive (0, 0.01, 0.02, 0.05, 0.10), and several demographic histories (Constant, African, European; [7]).

For both iHS and $iHH12$ scans, we normalized scores with respect to the neutral simulations and calculated the critical threshold for the most extreme 1% of scores. Using non-overlapping 100 kb windows across the genome, we calculated the fraction of scores in each window above this threshold. The top 1% of windows are identified as putatively under positive selection. This scheme controls the false positive rate to be no greater than 1%.

$iHH12$ has good power to detect hard and soft sweeps (Fig 1A, 1C, and 1E in S1 Appendix) and has improved power to identify both types of sweeps over iHS (Fig 1B, 1D, and 1F in S1 Appendix), particularly under realistic models of human demography.

Computing iHS and iHH12 scores in the Thousand Genomes Project (TGP)

We used `selscan` to compute both iHS and $iHH12$ scores for phase 3 TGP [8] phased whole genome sequences with a genetic map from HapMap3 [9]. Genetic map locations for sites not present in HapMap3 were linearly interpolated. The statistics were calculated for each population separately, and variants of frequency < 0.05 were filtered by `selscan`. All `selscan` runs used default parameters.

Using `selscan`'s companion program `norm`, for each population we normalized $iHH12$ scores genome-wide and normalized iHS scores in 1% frequency bins genome-wide. We identified the critical threshold representing the most extreme 1% of scores for each statistic. Then, to identify putative regions under selection, we partitioned the genome into non-overlapping 100 kb windows, and then we calculated the fraction of scores in each window above this threshold. The top 1% of windows were identified as putatively under positive selection. This scheme controlled the false positive rate to be no greater than 1%.

Figure 1

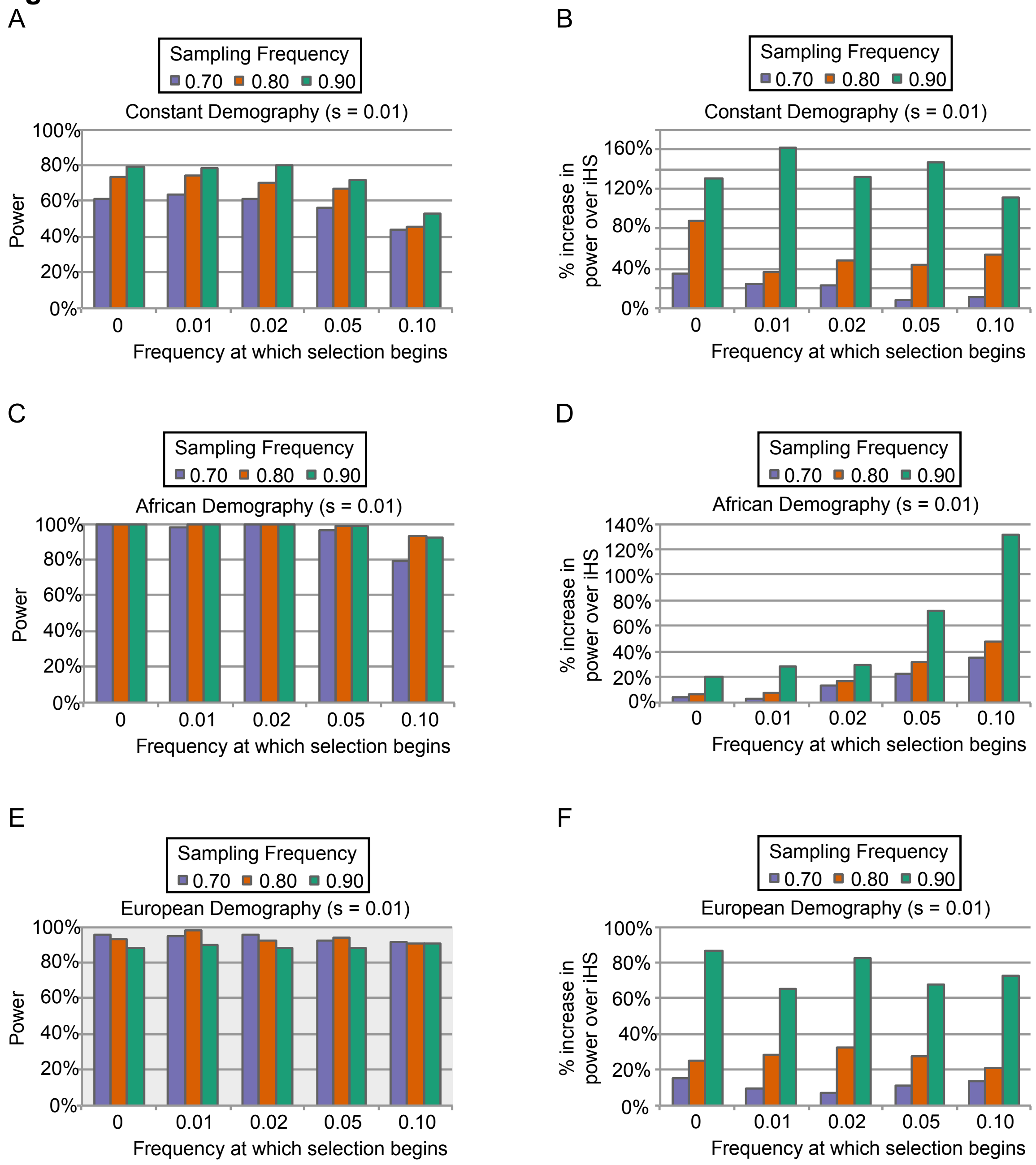

Fig 1. Power of $iHH12$ and comparison with iHS. Simulated power of $iHH12$ (A), (C), and (E) under varying parameters and comparison with iHS power (B), (D), and (F) in the same scenario. Panels (A) and (B) show results for a constant demography; panels (C) and (D) show results for an African demography; and panels (E) and (F) show results for a European demography. Non-constant demographies are from Gutenkunst et al. [10]. When the frequency at which selection begins is > 0, the sweep is considered soft. All simulations assume a selection coefficient of $s = 0.01$.

References

1. Garud NR, Messer PW, Buzbas EO, Petrov DA. Recent selective sweeps in North American Drosophila melanogaster show signatures of soft sweeps. PLoS Genet. 2015;11: e1005004. doi:10.1371/journal.pgen.1005004
2. Sabeti PC, Reich DE, Higgins JM, Levine HZP, Richter DJ, Schaffner SF, et al. Detecting recent positive selection in the human genome from haplotype structure. Nature. 2002;419: 832–837. doi:10.1038/nature01027.1.
3. Voight BF, Kudaravalli S, Wen X, Pritchard JK. A map of recent positive selection in the human genome. PLoS Biol. 2006;4: 0446–0458. doi:10.1371/journal.pbio.0040072
4. Sabeti PC, Varilly P, Fry B, Lohmueller J, Hostetter E, Cotsapas C, et al. Genome-wide detection and characterization of positive selection in human populations. Nature. 2007;449: 913–8. doi:10.1038/nature06250
5. Szpiech ZA, Hernandez RD. selscan: An efficient multithreaded program to perform EHH-based scans for positive selection. Mol Biol Evol. 2014;31: 2824–2827. doi:10.1093/molbev/msu211
6. Hudson RR. Generating samples under a Wright-Fisher neutral model of genetic variation. Bioinformatics. 2002;18: 337–338. doi:10.1093/bioinformatics/18.2.337
7. Gutenkunst RN, Hernandez RD, Williamson SH, Bustamante CD. Inferring the joint demographic history of multiple populations from multidimensional SNP frequency data. PLoS Genet. 2009;5: e1000695. doi:10.1371/journal.pgen.1000695
8. Auton A, Abecasis GR, Altshuler DM, Durbin RM, Abecasis GR, Bentley DR, et al. A global reference for human genetic variation. Nature. 2015;526: 68–74. doi:10.1038/nature15393
9. The International HapMap 3 Consortium, Altshuler DM, Gibbs RA, Peltonen L, Dermitzakis E, Schaffner SF, et al. Integrating common and rare genetic variation in diverse human populations. Nature. 2010;467: 52–8. doi:10.1038/nature09298
10. Gutenkunst RN, Hernandez RD, Williamson SH, Bustamante CD. Inferring the joint demographic history of multiple populations from multidimensional SNP frequency data. PLoS Genet. 2009;5: e1000695. doi:10.1371/journal.pgen.1000695
