# Supplementary material for: Human demographic history has amplified the effects of background selection across the genome
Source: PLoS Genet. 2018 Jun 18;14(6):e1007387. doi: 10.1371/journal.pgen.1007387 (PMC6056204; doi:10.1371/journal.pgen.1007387)
Supplement: S1 Text — (PDF) [file pgen.1007387.s003.pdf]

S1 Text

Recent admixture has not altered the impact of selection at linked sites

We investigated whether the effects of selection at linked sites have remained consistent across human populations that have experienced recent admixture. To do so, we measured normalized and relative diversity (more precisely, heterozygosity) as a function of *B* in the 6 admixed TGP populations (ASW, ACB, CLM, MXL, PEL, and PUR). We first used local ancestry to divide up admixed samples into genomic segments that are homozygous for a specific local ancestry (i.e., African, European, or Native American). These homozygous ancestral segments are simply regions of the genome in which both maternal and paternal copies of an individual's chromosomes were inferred to have the same ancestral label. To do this, we used the ancestry deconvolution results generated by the 1000 Genomes Project Consortium (see ftp://ftp.1000genomes.ebi.ac.uk/vol1/ftp/technical/working/20140818_ancestry_deconvolution/README_20140721_phase3_ancestry_deconvolution). Briefly, the local-ancesty inference tool, RFMix [1], was run across the ACB, ASW, CLM, MXL, PEL, and PUR phase 3 TGP samples. For the reference panel, 50 unrelated shapeit2 [2] trio-phased YRI and CEU samples each (from phase 3 TGP) and 43 shapeit2 population-phased Native American samples (from Ref. [3]) were used. We utilized local ancestry tracks that were inferred by RFMix using "trio-phased" mode.

Admixed samples were then parsed for all genomic segments homozygous for each particular ancestry (i.e., African, European, or Native American). These homozygous segments were also filtered according to the 13-filter set described in the "Filtering and ascertainment scheme" section of Materials and Methods in the main text. Heterozygosity was then calculated across admixed samples for each set of homozygous ancestries and *B* quantile bins. Samples were included in this analysis only if the total length of their genome that passed all filters for the particular ancestry and *B* quantile bin was greater than 1 Mb. Additionally, per-site

heterozygosity estimates for each ancestry and *B* quantile set were averaged across all admixed samples, regardless of their TGP population of origin. Heterozygosity was also normalized by divergence with Rhesus macaque (see Materials and Methods in the main text). See Table E in S1 Text for total number of Mb used in these analyses. For comparison, heterozygosity was also calculated across the 4 continental groups using the same 13-filter set and as a function of the same *B* quantile bins.

Across all *B* quantile bins, normalized diversity (heterozygosity/divergence) in African and European ancestry segments closely matched the values observed in their non-admixed counterparts (S11 Fig A in S1 Text). However, normalized diversity was significantly lower in the Native American ancestry segments of admixed individuals than in the East Asian continental group (S11 Fig A in S1 Text). This was expected given the more recent divergence of Native American populations and the strong population bottleneck they experienced migrating into the Americas [4-6].

Overall, patterns of relative diversity across local ancestries were similar to the broader analyses of the 20 non-admixed populations, with a consistent rank order of decreasing relative diversity observed for African, European, and Native American ancestral segments. However, for relative diversity calculated using the lowest 1% *B* quantile bin (i.e., where selection at linked sites is expected to be strongest), relative diversity in Native American ancestry segments was observed to be greater than for the European continental group or European local ancestry segments, which was inconsistent with the other *B* quantile bins.

Linear regression of F_{ST} on recombination rate and multiple linear regression of F_{ST} on recombination rate and *B*.

F_{ST} calculations were performed as a function of 2% recombination rate quantile bins between every pair of non-admixed phase 3 TGP populations in an identical fashion as was done for *B* (see Materials and Methods in main text). To do so, we annotated sites based on the recombination rate estimates from the HapMap II GRCh37 genetic map. To annotate sites in

phase 3 that were not in HapMap II, recombination rates were interpolated to the midway point between the preceding and following positions in HapMap II. If the difference between successive HapMap II positions was greater than 18,848 base pairs (the first standard deviation for the distribution of distances between positions in HapMap II), then the recombination rate was only extended out 9,424 base pairs beyond the focal position. Positions beyond this distance were then ignored during analysis in which the recombination rate was used. Recombination rate quantiles were calculated using the genome-wide distribution of recombination rates (i.e., the distribution of recombination rates across all sites, including those that are not polymorphic in the data set) resulting from the procedure described above.

Simple linear regression was then conducted using the linear model $F_{ST} = \beta_0 + \beta_1\rho + \varepsilon$ (where ρ is recombination rate). Recombination rate was scaled to be between 0 and 1 (the minimum and maximum observed recombination rate was 0.0 cM/Mb and 126.88 cM/Mb, respectively) to aid in the comparison of the regression coefficient with *B*. Earlier studies using SNP array data have shown that F_{ST} and recombination rate are correlated in humans [7]. We could only partially replicate these findings when we conducted linear regression with the model $F_{ST} = \beta_0 + \beta_1\rho + \varepsilon$. We observed that recombination rate only significantly predicts a change in F_{ST} across the genome for comparisons between South Asian and East Asian populations (S9 Fig, Table K in S1 Text). This result remained unchanged when performing robust linear regression for the model (Table G in S1 Text).

Since the correlation between F_{ST} and recombination rate was previously documented as being strongest in coding regions [7], where the effects of selection at linked sites are also expected to be strongest, we investigated whether recombination rate provides added value, in addition to *B*, as an explanatory variable for predicting F_{ST} by using multiple linear regression. To do so, we first split the genome into 2% recombination rate quantile bins and further subdivided each of these bins into 4% *B* quantile bins (50×25 = 1,250 bins total). We then

measured F_{ST} within each bin. We also partitioned sites in the reverse order (2% B bins followed by 4% recombination rate bins) and repeated all analyses. Our choice of total number of bins resulted in a minimum of 320 SNPs per bin for estimating F_{ST} between any two populations, which should be sufficient to avoid errors when estimating F_{ST} across multiple loci [8]. As with the simple linear regression step, recombination rate was scaled to be between 0 and 1 and the mean of the bounds defining each quantile bin was used when defining the explanatory variables. After performing multiple linear regression of F_{ST} on B, recombination rate (ρ), and an interaction term between the two ($B\rho$) with the linear model $F_{ST} = \beta_0 + \beta_1 B + \beta_2\rho + \beta_3 B\rho + \varepsilon$, we observed that B was a statistically significant predictor ($p < 1e\text{-}04$) for F_{ST} across all population comparisons regardless of how we partitioned sites (Table H in S1 Text). This result remained unchanged when performing robust regression. In contrast, recombination rate exhibited sporadic significance as an explanatory variable for F_{ST} across population comparisons and was dependent upon how sites were partitioned (i.e., whether we first partitioned by B or by recombination rate) (Table H in S1 Text). Furthermore, strong differences between the two binning schemes were observed for the magnitude of the recombination rate regression coefficient for certain population comparisons (e.g., African vs. East Asian and South Asian vs. East Asian), while the coefficients for B were consistently similar across binning schemes. The direction in which recombination rate explained F_{ST} was also inconsistent across different population comparisons, with European vs. South Asian and European vs. East Asian comparisons showing a significant positive change in F_{ST} as a function of increasing recombination rate. This result was contrary to an expectation of decreasing F_{ST} as a function of increasing recombination rate [7]. We also failed to observe consistent effects from the interaction term for B and recombination rate on F_{ST} across population comparisons or binning schemes (Table H in S1 Text). Performing robust regression on the model did not change these results. However, in contrast to recombination rate, when the model was performed utilizing all

TGP populations (i.e., the "Global" estimate), the interaction term was significant in explaining F_{ST} across both types of binning schemes.

To aid in visualizing the results of our multidimensional linear model, we plotted F_{ST} for each population comparison as a function of recombination rate (across 4% quantile bins) while conditioning on B (S10 Fig A in S1 Text). We also plotted points in the reciprocal direction, with F_{ST} being plotted as a function of B while conditioning on recombination rate (S10 Fig B in S1 Text). These data points were derived from the same points used as input for the multiple linear regression model described above. These specific results for F_{ST} between African and South Asian populations showed that B separated different levels of F_{ST} across most recombination rate bins (S10 Fig A, Table I in S1 Text). Furthermore, regardless of how B was conditioned on recombination rate, it still exhibited a strong trend of increasing F_{ST} as it decreased (i.e., in the direction of stronger BGS) (S10 Fig B, Table J in S1 Text). These patterns were imperfect though, and statistical significance was not always attained, especially for comparisons between non-African populations (S6 Fig, Table J in S1 Text). However, greater separation in F_{ST} was generally achieved when conditioning recombination rate on B and the slope was always negative when plotting F_{ST} against B, regardless of which recombination rate percentile bin B was conditioned on.

SFS_CODE command line example

Below is a representative SFS_CODE command for running a simulation of BGS and human demography with 20.46% of sites experiencing deleterious mutation in two 1Mb flanking regions surrounding a neutral 30kb central region. Note that we simulate two distributions of fitness effects for purifying selection here (see Materials and Methods in the main text for details). More specifically, this is given by the command `-W 2 -0.3394 0.184 0.00040244 0.0415 0.00515625` (see below) where the '`-`' in front of `0.3394` allows us to draw from a negative gamma distribution with parameters (`0.184, 0.00040244`) for 33.94% of selected sites and from a negative gamma distribution with parameters (`0.0415, 0.00515625`) for

66.06% of selected sites. This ability to draw from two negative gamma distributions of fitness effects is a special option not available in the general distribution of SFS_CODE. It is available in the tar file S1_File_SFS_CODE_torres_et_al_2018.tar.gz in supplemental files.

```
sfs_code 3 1 -A -r 6.0443e-05 -N 18449 -s 1100 -n 100 -TS 0.437017 0 1
-TS 0.546498 1 2 -TE 0.5994242 -Td 0 P 0 2.10709 -Td 0.437017 P 1
0.152957396219 -Td 0.546498 P 1 0.573964845871 -Tg 0.546498 P 1
60.0453856768 -Td 0.546498 P 2 0.221523138739 -Tg 0.546498 P 2
95.5344964867 -Tm 0.437017 P 0 1 6.0846016512 -Tm 0.437017 P 1 0
0.9306848256 -Tm 0.546498 L 0.39374558703 0.104413684315
0.034567778862 0.067228907022 0.0035379117753 0.0259471614066 -t
0.0002650345 -L 11 200000 200000 200000 200000 200000 30000 200000
200000 200000 200000 200000 -v L A 40920 -v L 5 30000 -W 2 -0.3394
0.184 0.00040244 0.0415 0.00515625 -W L 5 0 --printLocus 5 -a N -Tn
0.437017 100 -Tn 0.546498 100 -Tn 0 R 0.00271017399317 100
```

References

1. Maples BK, Gravel S, Kenny EE, Bustamante CD. RFMix: A discriminative modeling approach for rapid and robust local-ancestry inference. Am J Hum Genet. 2013;93: 278–288. doi:10.1016/j.ajhg.2013.06.020
2. O'Connell J, Gurdasani D, Delaneau O, Pirastu N, Ulivi S, Cocca M, et al. A general approach for haplotype phasing across the full spectrum of relatedness. PLoS Genet. 2014;10: e1004234. doi:10.1371/journal.pgen.1004234
3. Mao X, Bigham AW, Mei R, Gutierrez G, Weiss KM, Brutsaert TD, et al. A genomewide admixture mapping panel for Hispanic/Latino populations. Am J Hum Genet. 2007;80: 1171–1178. doi:10.1086/518564
4. Ramachandran S, Deshpande O, Roseman CC, Rosenberg NA, Feldman MW, Cavalli-Sforza LL. Support from the relationship of genetic and geographic distance in human populations for a serial founder effect originating in Africa. Proc Natl Acad Sci. 2005;102: 15942–15947. doi:10.1073/pnas.0507611102
5. Moreno-Estrada A, Gignoux CR, Fernández-López JC, Zakharia F, Sikora M, Contreras AV, et al. The genetics of Mexico recapitulates Native American substructure and affects biomedical traits. Science. 2014;344: 1280–1285. doi:10.1126/science.1251688
6. Hey J. On the number of new world founders: A population genetic portrait of the peopling of the Americas. PLoS Biol. 2005;3: 0965–0975. doi:10.1371/journal.pbio.0030193
7. Keinan A, Reich D. Human population differentiation is strongly correlated with local recombination rate. PLoS Genet. 2010;6: e1000886. doi:10.1371/journal.pgen.1000886
8. Willing EM, Dreyer C, van Oosterhout C. Estimates of genetic differentiation measured by F_{ST} do not necessarily require large sample sizes when using many SNP markers. PLoS One. 2012;7: e42649. doi:10.1371/journal.pone.0042649

Table A

Parameters	B ≥ 0.994	four-fold degenerate
$N_{Ancestral}$	18,449	17,118
N_{AFR}	38,874	47,537
N_{Bott}	5,946	6,408
N_{EUR0}	3,413	4,331
N_{EUR}	81,901	100,614
N_{EASN0}	1,317	1,678
N_{EASN}	206,804	266,616
$T_{AFR}+T_{Bott}+T_{EUR\_EASN}$ (kya)	552,939	413,337
$T_{Bott}+T_{EUR\_EASN}$ (kya)	149,813	198,603
T_{EUR_EASN} (kya)	48,822	69,584
r_{EUR} (%)	0.163	0.113
r_{EASN} (%)	0.259	0.182
$m_{AFR-Bott}$ ($x10^{-5}$)	7.83	7.02
$m_{AFR-EUR}$ ($x10^{-5}$)	0.51	0.47
$m_{AFR-EASN}$ ($x10^{-5}$)	0.13	0.18
$m_{EUR-EASN}$ ($x10^{-5}$)	0.98	1.14

Table A. Inferred parameters from running dadi on TGP CG data across neutral regions in the highest 1% B value bin (B ≥ 0.994) and across four-fold degenerate sites. The demographic model inferred is the Out-of-Africa demographic model of Gutenkunst et al. 2009 (Ref. [7] in main text). Time parameters, T, assume a generation time of 25 years per generation. Growth rates, r, and migration rates, m, are per generation. Parameters with subscript, "*Bott*", represent parameters inferred for the ancestral European and East Asian out-of-Africa bottleneck population. Time parameters with subscript "*EUR_EASN*" represent the European-East Asian population split.

Table B. TGP Complete Genomics dataset unrelated YRI, CHS, and CEU samples.

Population	Sample ID
YRI	NA18498
YRI	NA18499
YRI	NA18501
YRI	NA18502
YRI	NA18504
YRI	NA18505
YRI	NA19107
YRI	NA18507
YRI	NA18508
YRI	NA18522
YRI	NA18870
YRI	NA18871
YRI	NA18909
YRI	NA18916
YRI	NA18917
YRI	NA18923
YRI	NA18924
YRI	NA18933
YRI	NA18934
YRI	NA19186
YRI	NA19093
YRI	NA19097
YRI	NA19137
YRI	NA19138
YRI	NA19200
YRI	NA19201
YRI	NA19171
YRI	NA19172
YRI	NA19210
YRI	NA19159
YRI	NA19160
YRI	NA19222
YRI	NA19116
YRI	NA19152
YRI	NA19153
YRI	NA19143
YRI	NA19144
YRI	NA19146
YRI	NA19147

Population	Sample ID
CHS	HG00608
CHS	HG00610
CHS	HG00611
CHS	HG00613
CHS	HG00614
CHS	HG00619
CHS	HG00620
CHS	HG00625
CHS	HG00626
CHS	HG00628
CHS	HG00629
CHS	HG00650
CHS	HG00651
CHS	HG00653
CHS	HG00654
CHS	HG00662
CHS	HG00663
CHS	HG00671
CHS	HG00672
CHS	HG00683
CHS	HG00684
CHS	HG00689
CHS	HG00690
CHS	HG00692
CHS	HG00693
CEU	NA06984
CEU	NA06989
CEU	NA12347
CEU	NA12348
CEU	NA12340
CEU	NA12341
CEU	NA06994
CEU	NA07000
CEU	NA07346
CEU	NA07347
CEU	NA12045
CEU	NA12046
CEU	NA11829
CEU	NA11830

YRI	NA19113
YRI	NA19114
YRI	NA19256
YRI	NA19257
YRI	NA19117
YRI	NA19118
YRI	NA19130
YRI	NA19098
YRI	NA19189
YRI	NA19190
YRI	NA19236
YRI	NA19238
YRI	NA19239
YRI	NA19247
CHS	HG00403
CHS	HG00404
CHS	HG00406
CHS	HG00407
CHS	HG00421
CHS	HG00422
CHS	HG00436
CHS	HG00437
CHS	HG00442
CHS	HG00443
CHS	HG00448
CHS	HG00449
CHS	HG00463
CHS	HG00464
CHS	HG00472
CHS	HG00473
CHS	HG00475
CHS	HG00476
CHS	HG00478
CHS	HG00479
CHS	HG00530
CHS	HG00531
CHS	HG00533
CHS	HG00534
CHS	HG00536
CHS	HG00537
CHS	HG00542

CEU	NA11831
CEU	NA11832
CEU	NA12489
CEU	NA12546
CEU	NA12399
CEU	NA12400
CEU	NA12413
CEU	NA12414
CEU	NA12716
CEU	NA12717
CEU	NA11994
CEU	NA11995
CEU	NA11893
CEU	NA11894
CEU	NA12155
CEU	NA12156
CEU	NA12272
CEU	NA12273
CEU	NA12003
CEU	NA12004
CEU	NA12005
CEU	NA12006
CEU	NA12286
CEU	NA12287
CEU	NA11919
CEU	NA11920
CEU	NA11930
CEU	NA11931
CEU	NA12750
CEU	NA12751
CEU	NA12760
CEU	NA12761
CEU	NA12762
CEU	NA12763
CEU	NA12775
CEU	NA12776
CEU	NA12777
CEU	NA12778
CEU	NA12812
CEU	NA12813
CEU	NA12814

CHS	HG00543		CEU	NA12815
CHS	HG00556		CEU	NA12827
CHS	HG00557		CEU	NA12828
CHS	HG00559		CEU	NA12829
CHS	HG00560		CEU	NA12830
CHS	HG00589		CEU	NA12842
CHS	HG00590		CEU	NA12843
CHS	HG00592		CEU	NA12872
CHS	HG00593		CEU	NA12873
CHS	HG00607			

Table C

**Henn et al. 2016 samples**		
**SampleID**	**Number of Sites**	**Mean Depth**
HGDP00991	2,207,845	6.96118
HGDP00987	2,229,426	7.19132
HGDP01036	2,373,023	11.6072
HGDP00992	2,452,509	12.1913
HGDP01029	2,415,792	12.3526
HGDP01032	2,407,400	12.8113
**Kidd et al. 2014 samples**		
**SampleID**	**Number of Sites**	**Mean Depth**
SA1000A	547,527	2.56481
SA1025A	2,136,905	9.1239
**Kim et al. 2014 samples**		
**SampleID**	**Number of Sites**	**Mean Depth**
KB2	2,756,225	27.5951
NB1	2,599,220	28.0148
MD8	2,777,871	38.4532
NB8	2,778,198	40.1789
KB1	2,757,336	50.5629

Table C. Number of polymorphic sites and mean depth coverage of 13 KhoeSan samples used for SNP ascertainment in calculations of F_{ST} from studies of Henn et al. 2016, Kidd et al. 2014, and Kim et al. 2014 (Refs. [95-97] in main text).

Table D

	lowest 1% *B*	lowest 5% *B*	lowest 10% *B*	lowest 25% *B*	highest 1% *B*
**filters**	7.59	40.42	87.86	246.59	13.1
**filters + gBGC and hotspots removal**	7.26	38.68	83.75	231.71	7.94

Table D. Total number of Mb in the human genome passing the set of 13 filters described in Materials and Methods that were used for calculating pairwise genetic diversity (π) for each quantile of *B*. The bottom row is the total number Mb when including the set of filters to remove regions sensitive to GC-biased gene conversion (gBGC) or sites in recombination hotspots. Additionally, these totals only include those 100 kb regions that had a minimum of 10 kb of divergence information for Rhesus macaque (see Materials and Methods).

Table E

**Ancestry**	**lowest 1% *B***	**lowest 5% *B***	**lowest 10% *B***	**lowest 25% *B***	**highest 1% *B***
African	841.97	4471.54	9720.15	27333.95	1447.04
European	815.74	4296.69	9293.04	26034.57	1366.26
Native American	497.29	2603.12	5640.13	15776.71	834.46

Table E. Total number of Mb of homozygous ancestry that passed all filters and were used in the analyses of admixed samples in the 6 admixed TGP populations (ACB, ASW, CLM, MXL, PEL, PUR) for each quantile of *B*. Additionally, these totals only include those 100 kb regions that had a minimum of 10 kb of divergence information for Rhesus macaque (see Materials and Methods).

Table F

	AFR vs. EASN	AFR vs. EUR	AFR vs. SASN	EUR vs. SASN	EUR vs. EASN	SASN vs. EASN	Global
β_0 **± SEM (p-value)**	0.2043 ± 0.0036 (< 1e-04)	0.1724 ± 0.0031 (< 1e-04)	0.1591 ± 0.0028 (< 1e-04)	0.0459 ± 0.0011 (< 1e-04)	0.1214 ± 0.0029 (< 1e-04)	0.0880 ± 0.0021 (< 1e-04)	0.1337 ± 0.0020 (< 1e-04)
β_1 **± SEM (p-value)**	-0.0428 ± 0.0042 (< 1e-04)	-0.0363 ± 0.0036 (< 1e-04)	-0.0344 ± 0.0033 (< 1e-04)	-0.0099 ± 0.0013 (< 1e-04)	-0.0168 ± 0.0034 (< 1e-04)	-0.0223 ± 0.0024 (< 1e-04)	-0.0295 ± 0.0023 (< 1e-04)

Table F. Regression coefficient estimates for robust linear regression of F_{ST} on B. To apply an additional test for the relationship between background selection and F_{ST} that is more robust to outlier points or points with high influence, we performed robust linear regression using M-estimation with Huber weighting. Robust linear regression was run on the same data as was used for the linear regression described for Table 1 in the main text. Each column gives the regression coefficients for the linear model $F_{ST} = \beta_0 + \beta_1 B$, where B represents the mean background selection coefficient for the bin being tested and F_{ST} is the estimated F_{ST} for all population comparisons within a particular pair of continental groups. The final column, "Global", gives the regression coefficients for the linear model applied to all pairwise population comparisons (150 total). Standard errors of the mean (SEM) for β_0 and β_1 were calculated from 1,000 bootstrap iterations (see Materials and Methods). P-values are derived from a Wald (F-distribution) test on the F-statistic for the corresponding regression coefficient.

Table G

	AFR vs. EASN	AFR vs. EUR	AFR vs. SASN	EUR vs. SASN	EUR vs. EASN	SASN vs. EASN	Global
β_0 ± SEM (p-value)	0.1688 ± 0.0006 (< 1e-04)	0.1425 ± 0.0006 (< 1e-04)	0.1308 ± 0.0005 (< 1e-04)	0.0376 ± 0.0002 (< 1e-04)	0.1073 ± 0.0006 (< 1e-04)	0.0699 ± 0.0004 (< 1e-04)	0.1093 ± 0.0003 (< 1e-04)
β_1 ± SEM (p-value)	-0.0001 ± 0.0026 (0.9755)	0.0007 ± 0.0022 (0.7591)	0.0008 ± 0.0021 (0.7281)	-0.0015 ± 0.0008 (0.4317)	0.0004 ± 0.0020 (0.7869)	-0.0046 ± 0.0014 (0.0225)	-0.0009 ± 0.0013 (0.9022)

Table G. Regression coefficient estimates for robust linear regression of F_{ST} on recombination rate. To apply an additional test for the relationship between recombination rate and F_{ST} that is more robust to outlier points or points with high influence, we performed robust linear regression using M-estimation with Huber weighting. Robust linear regression was run on the same data as was used for the linear regression described for Table K in S1 Text. Each column gives the regression coefficients for the linear model $F_{ST} = \beta_0 + \beta_1\rho$, where ρ represents the mean recombination rate for the bin being tested and F_{ST} is the estimated F_{ST} for all population comparisons within a particular pair of continental groups. The final column, "Global", gives the regression coefficients for the linear model applied to all pairwise population comparisons (150 total). When performing the regression, ρ was first scaled to between 0 and 1, such that 1 represents the maximum observed recombination rate (126.88 cM/Mb) and 0 represents the minimum observed recombination rate (0.0 cM/Mb). Standard errors of the mean (SEM) for β_0 and β_1 were calculated from 1,000 bootstrap iterations (see Materials and Methods). P-values are derived from a Wald (F-distribution) test on the F-statistic for the corresponding regression coefficient.

Table H

	AFR vs. EASN		AFR vs. EUR		AFR vs. SASN		EUR vs. SASN		EUR vs. EASN		SASN vs. EASN		Global	
	**ρ by *B***	***B* by ρ**	**ρ by *B***	***B* by ρ**	**ρ by *B***	***B* by ρ**	**ρ by *B***	***B* by ρ**	**ρ by *B***	***B* by ρ**	**ρ by *B***	***B* by ρ**	**ρ by *B***	***B* by ρ**
β_0 **(p-value)**	0.2037 (< 1e-04)	0.2054 (< 1e-04)	0.1713 (< 1e-04)	0.1725 (< 1e-04)	0.1594 (< 1e-04)	0.1609 (< 1e-04)	0.0450 (< 1e-04)	0.0451 (< 1e-04)	0.1200 (< 1e-04)	0.1209 (< 1e-04)	0.0888 (< 1e-04)	0.0903 (< 1e-04)	0.1314 (< 1e-04)	0.1325 (< 1e-04)
β_0 **(p-value) - robust**	0.1995 (< 1e-04)	0.2019 (< 1e-04)	0.1696 (< 1e-04)	0.1709 (< 1e-04)	0.1567 (< 1e-04)	0.1589 (< 1e-04)	0.0450 (< 1e-04)	0.0449 (< 1e-04)	0.1185 (< 1e-04)	0.1199 (< 1e-04)	0.0853 (< 1e-04)	0.0868 (< 1e-04)	0.1314 (< 1e-04)	0.1329 (< 1e-04)
β_1 **(p-value)**	-0.0427 (< 1e-04)	-0.0448 (< 1e-04)	-0.0355 (< 1e-04)	-0.0368 (< 1e-04)	-0.0353 (< 1e-04)	-0.0371 (< 1e-04)	-0.0093 (< 1e-04)	-0.0094 (< 1e-04)	-0.0157 (< 1e-04)	-0.0168 (< 1e-04)	-0.0245 (< 1e-04)	-0.0262 (< 1e-04)	-0.0272 (< 1e-04)	-0.0285 (< 1e-04)
β_1 **(p-value) - robust**	-0.0380 (< 1e-04)	-0.0408 (< 1e-04)	-0.0335 (< 1e-04)	-0.0350 (< 1e-04)	-0.0323 (< 1e-04)	-0.0350 (< 1e-04)	-0.0092 (< 1e-04)	-0.0091 (< 1e-04)	-0.0140 (< 1e-04)	-0.0157 (< 1e-04)	-0.0200 (< 1e-04)	-0.0218 (< 1e-04)	-0.0271 (< 1e-04)	-0.0288 (< 1e-04)
β_2 **(p-value)**	-0.0448 (< 1e-04)	-0.0166 (0.0018)	-0.0300 (< 1e-04)	-0.0285 (< 1e-04)	-0.0332 (< 1e-04)	-0.0324 (< 1e-04)	-0.0039 (0.2212)	0.0095 (0.0002)	0.0146 (0.0046)	0.0142 (0.0008)	-0.0252 (< 1e-04)	-0.0081 (0.0481)	-0.0204 (0.0389)	-0.0103 (0.1963)
β_2 **(p-value) - robust**	-0.0306 (< 1e-04)	-0.0039 (0.4085)	-0.0225 (< 1e-04)	-0.0230 (< 1e-04)	-0.0226 (< 1e-04)	-0.0243 (< 1e-04)	-0.0035 (0.2843)	0.0104 (< 1e-04)	0.0178 (< 1e-04)	0.0180 (< 1e-04)	-0.0125 (0.0066)	0.0049 (0.2047)	-0.0176 (0.0932)	-0.0096 (0.2576)
β_3 **(p-value)**	0.0588 (< 1e-04)	0.0345 (< 1e-04)	0.0419 (< 1e-04)	0.0419 (< 1e-04)	0.0458 (< 1e-04)	0.0488 (< 1e-04)	0.0052 (0.1387)	-0.0094 (0.0018)	-0.0107 (0.0585)	-0.0084 (0.0886)	0.0286 (< 1e-04)	0.0127 (0.0083)	0.0283 (0.0092)	0.0200 (0.0322)
β_3 **(p-value) - robust**	0.0436 (< 1e-04)	0.0201 (0.0002)	0.0338 (< 1e-04)	0.0358 (< 1e-04)	0.0343 (< 1e-04)	0.0400 (< 1e-04)	0.0047 (0.1889)	-0.0103 (0.0009)	-0.0146 (0.0032)	-0.0127 (0.0047)	0.0141 (0.0053)	-0.0023 (0.6046)	0.0254 (0.0269)	0.0196 (0.0484)

Table H. Multiple Linear Regression and Robust Regression of F_{ST} on *B* and recombination rate. We performed multiple linear regression of *B* and recombination rate on F_{ST} estimated for 1,250 bins of either 1) 50 2% quantile recombination rate bins by 25 4% quantile *B* bins ("ρ by *B*" in the table) or 2) 50 2% quantile *B* bins by 25 4% quantile recombination rate bins ("*B* by ρ" in the table). Each column gives the regression coefficients for the linear model $F_{ST} = \beta_0 + \beta_1 B + \beta_2 \rho + \beta_3 B\rho$, where *B* represents the mean background selection coefficient for the bin being tested, ρ represents the mean recombination rate for the bin being tested, and $B\rho$ is an interaction term for the background selection coefficient and recombination rate. F_{ST} is the estimated F_{ST} for all population comparisons within a particular pair of continental groups except for the final pair of columns, for which the linear model was applied to all pairwise population estimates of F_{ST} (150 total). When performing the regression, ρ was first scaled to between 0 and 1, such that 1 represents the maximum observed recombination rate (126.88 cM/Mb) and 0 represents the minimum observed recombination rate (0.0 cM/Mb). We also performed robust linear regression using M-estimation with Huber weighting on the same linear model, $F_{ST} = \beta_0 + \beta_1 B + \beta_2 \rho + \beta_3 B\rho$. The regression coefficients from performing robust linear regression are indicated by the rows labeled "robust" in the table. P-values for normal linear regression are derived from a two-sided t-test of the t-value for the corresponding regression coefficients. P-values for robust linear regression are derived from a Wald (F-distribution) test on the F-statistic for the corresponding regression coefficients.

Table I

	AFR vs. EASN			AFR vs. EUR			AFR vs. SASN			EUR vs. SASN			EUR vs. EASN			SASN vs. EASN		
**quantile *B* bin**	**0-2% *B***	**8-10% *B***	**98-100% *B***	**0-2% *B***	**8-10% *B***	**98-100% *B***	**0-2% *B***	**8-10% *B***	**98-100% *B***	**0-2% *B***	**8-10% *B***	**98-100% *B***	**0-2% *B***	**8-10% *B***	**98-100% *B***	**0-2% *B***	**8-10% *B***	**98-100% *B***
**β_0 (p-value)**	0.2058<br>(< 1e-04)	0.1838<br>(< 1e-04)	0.1561<br>(< 1e-04)	0.1689<br>(< 1e-04)	0.1463<br>(< 1e-04)	0.132<br>(< 1e-04)	0.1643<br>(< 1e-04)	0.1425<br>(< 1e-04)	0.1238<br>(< 1e-04)	0.041<br>(< 1e-04)	0.039<br>(< 1e-04)	0.0336<br>(< 1e-04)	0.1229<br>(< 1e-04)	0.1067<br>(< 1e-04)	0.0993<br>(< 1e-04)	0.1017<br>(< 1e-04)	0.0666<br>(< 1e-04)	0.0594<br>(< 1e-04)
**β_1 (p-value)**	-0.0464<br>(0.8263)	0.0489<br>(0.4957)	0.0157<br>(0.1663)	0.0529<br>(0.6589)	0.0279<br>(0.5937)	0.016<br>(0.167)	-0.0273<br>(0.7961)	0.002<br>(0.9735)	0.0106<br>(0.3121)	0.0506<br>(0.1034)	0.0083<br>(0.6497)	0.0028<br>(0.4086)	0.0658<br>(0.5656)	0.0461<br>(0.4479)	-0.0046<br>(0.7096)	-0.0434<br>(0.6934)	0.0136<br>(0.7103)	-7e-04<br>(0.9216)
r	-0.0462	0.1429	0.2856	0.0928	0.1121	0.2852	-0.0544	0.007	0.2107	0.3334	0.0955	0.1729	0.1207	0.159	-0.0784	-0.083	0.0782	-0.0207

Table I. Linear Regression of F_{ST} on recombination rate but conditioning on *B* quantiles. This table gives the results of running simple linear regression of F_{ST} on 25 4% quantile bins of recombination rate (ρ), while conditioning on 3 specific quantile bins of *B*. The specific quantile bins of *B* that were conditioned on were 0-2%, 8-10%, and 98-100%. Each column gives the regression coefficients for the linear model $F_{ST} = \beta_0 + \beta_1\rho + \varepsilon$ for each population comparison of F_{ST}. The correlation coefficient, *r*, between ρ and F_{ST} for each population comparison is shown in the bottom row. P-values are derived from a two-sided t-test of the t-value for the corresponding regression coefficient. The values in this table correspond directly to S10 Fig A and S6 Fig.

Table J

	AFR vs. EASN			AFR vs. EUR			AFR vs. SASN			EUR vs. SASN			EUR vs. EASN			SASN vs. EASN		
**quantile ρ bin**	**0-2% ρ**	**8-10% ρ**	**98-100% ρ**	**0-2% ρ**	**8-10% ρ**	**98-100% ρ**	**0-2% ρ**	**8-10% ρ**	**98-100% ρ**	**0-2% ρ**	**8-10% ρ**	**98-100% ρ**	**0-2% ρ**	**8-10% ρ**	**98-100% ρ**	**0-2% ρ**	**8-10% ρ**	**98-100% ρ**
**β_0 (p-value)**	0.2222<br>(< 1e-04)	0.2296<br>(< 1e-04)	0.1907<br>(< 1e-04)	0.1749<br>(< 1e-04)	0.1852<br>(< 1e-04)	0.1625<br>(< 1e-04)	0.1663<br>(< 1e-04)	0.1798<br>(< 1e-04)	0.1534<br>(< 1e-04)	0.0408<br>(< 1e-04)	0.0446<br>(< 1e-04)	0.0413<br>(< 1e-04)	0.1062<br>(< 1e-04)	0.1116<br>(< 1e-04)	0.1234<br>(< 1e-04)	0.0854<br>(< 1e-04)	0.0829<br>(< 1e-04)	0.0746<br>(< 1e-04)
**β_1 (p-value)**	-0.0545<br>(0.052)	-0.0822<br>(< 1e-04)	-0.0229<br>(0.016)	-0.0434<br>(0.0258)	-0.0601<br>(< 1e-04)	-0.0222<br>(4e-04)	-0.0397<br>(0.0506)	-0.0639<br>(< 1e-04)	-0.0218<br>(0.0015)	-0.0079<br>(0.1767)	-0.0084<br>(0.0025)	-0.0054<br>(0.1041)	-0.0036<br>(0.8313)	-0.0039<br>(0.6132)	-0.0182<br>(0.0429)	-0.0236<br>(0.0551)	-0.0221<br>(0.0024)	-0.0122<br>(0.0139)
r	-0.3929	-0.7865	-0.4767	-0.4451	-0.7599	-0.6562	-0.3951	-0.78	-0.6001	-0.2791	-0.578	-0.3328	-0.0449	-0.1062	-0.408	-0.3883	-0.5788	-0.4852

Table J. Linear Regression of F_{ST} on *B* but conditioning on recombination rate quantiles. This table gives the results of running simple linear regression of F_{ST} on 25 4% quantile bins of *B*, while conditioning on 3 specific quantile bins of recombination rate (ρ). The specific quantile bins of ρ that were conditioned on were 0-2%, 8-10%, and 98-100%. Each column gives the regression coefficients for the linear model $F_{ST} = \beta_0 + \beta_1 B + \varepsilon$ for each population comparison of F_{ST}. The correlation coefficient, *r*, between *B* and F_{ST} for each population comparison is shown in the bottom row. P-values are derived from a two-sided t-test of the t-value for the corresponding regression coefficient. The values in this table correspond directly to S10 Fig B and S6 Fig.

Table K

	AFR vs. EASN	AFR vs. EUR	AFR vs. SASN	EUR vs. SASN	EUR vs. EASN	SASN vs. EASN	Global
β_0<br>**± SEM**<br>**(p-value)**	0.1688<br>± 0.0007<br>(< 1e-04)	0.1422<br>± 0.0006<br>(< 1e-04)	0.1305<br>± 0.0006<br>(< 1e-04)	0.0373<br>± 0.0002<br>(< 1e-04)	0.1070<br>± 0.0006<br>(< 1e-04)	0.0688<br>± 0.0004<br>(< 1e-04)	0.1091<br>± 0.0003<br>(< 1e-04)
β_1<br>**± SEM**<br>**(p-value)**	-0.0009<br>± 0.0026<br>(0.7073)	0.0005<br>± 0.0022<br>(0.8454)	0.0005<br>± 0.0021<br>(0.8196)	-0.0015<br>± 0.0007<br>(0.3906)	0.0005<br>± 0.0021<br>(0.7002)	-0.0050<br>± 0.0014<br>(0.0363)	-0.0010<br>± 0.0012<br>(0.8842)
r<br>**± SEM**	-0.0106<br>± 0.0287	0.0055<br>± 0.0257	0.0065<br>± 0.0253	-0.0243<br>± 0.0119	0.0109<br>± 0.0379	-0.0592<br>± 0.0159	-0.0017<br>± 0.0021

Table K. Regression coefficient estimates for linear regression of F_{ST} on 2% quantile bins of recombination rate. The first two rows give the regression coefficients for the linear model $F_{ST} = \beta_0 + \beta_1\rho + \varepsilon$, where ρ represents the mean recombination rate for the bin being tested and F_{ST} is the estimated F_{ST} for all population comparisons within a particular pair of continental groups (given in the column header). The final column, "Global", gives the regression coefficients for the linear model applied to all pairwise population comparisons (150 total). When performing the regression, ρ was first scaled to between 0 and 1, such that 1 represents the maximum observed recombination rate (126.88 cM/Mb) and 0 represents the minimum observed recombination rate (0.0 cM/Mb). The correlation coefficient, r, between ρ and F_{ST} for each comparison is shown in the bottom row. Standard errors of the mean (SEM) for β_0, β_1, and r were calculated from 1,000 bootstrap iterations (see Materials and Methods). P-values are derived from a two-sided t-test of the t-value for the corresponding regression coefficient.

Table L. Phase 3 TGP population information and classification by continental group (or classified as admixed).

Population	Ethnic Group/Population	TGP Population Label	Continental Group (or Admixed)	Sample Size
Esan in Nigeria	Esan	ESN	AFR	99
Gambian in Western Division, Mandinka	Gambian	GWD	AFR	113
Luhya in Webuye, Kenya	Luhya	LWK	AFR	99
Mende in Sierra Leone	Mende	MSL	AFR	85
Yoruba in Ibadan, Nigeria	Yoruba	YRI	AFR	108
Utah residents (CEPH) with Northern and Western European ancestry	CEPH	CEU	EUR	99
British in England and Scotland	British	GBR	EUR	91
Finnish in Finland	Finnish	FIN	EUR	99
Iberian Populations in Spain	Spanish	IBS	EUR	107
Toscani in Italia	Tuscan	TSI	EUR	107
Bengali in Bangladesh	Bengali	BEB	SASN	86
Gujarati Indians in Houston, TX, USA	Gujarati	GIH	SASN	103
Indian Telugu in the UK	Telugu	ITU	SASN	102
Punjabi in Lahore, Pakistan	Punjabi	PJL	SASN	96
Sri Lankan Tamil in the UK	Tamil	STU	SASN	102
Chinese Dai in Xishuangbanna, China	Dai Chinese	CDX	EASN	93
Han Chinese in Beijing, China	Han Chinese	CHB	EASN	103
Southern Han Chinese	Southern Han Chinese	CHS	EASN	105
Japanese in Tokyo, Japan	Japanese	JPT	EASN	104
Kinh in Ho Chi Minh City, Vietnam	Kinh Vietnamese	KHV	EASN	99
African Caribbean in Barbados	Barbadian	ACB	Admixed	96
People with African Ancestry in Southwest USA	African-American SW	ASW	Admixed	61
Colombians in Medellin, Colombia	Colombian	CLM	Admixed	94
People with Mexican Ancestry in Los Angeles, CA, USA	Mexican-American	MXL	Admixed	64
Peruvians in Lima, Peru	Peruvian	PEL	Admixed	85
Puerto Ricans in Puerto Rico	Puerto Rican	PUR	Admixed	104

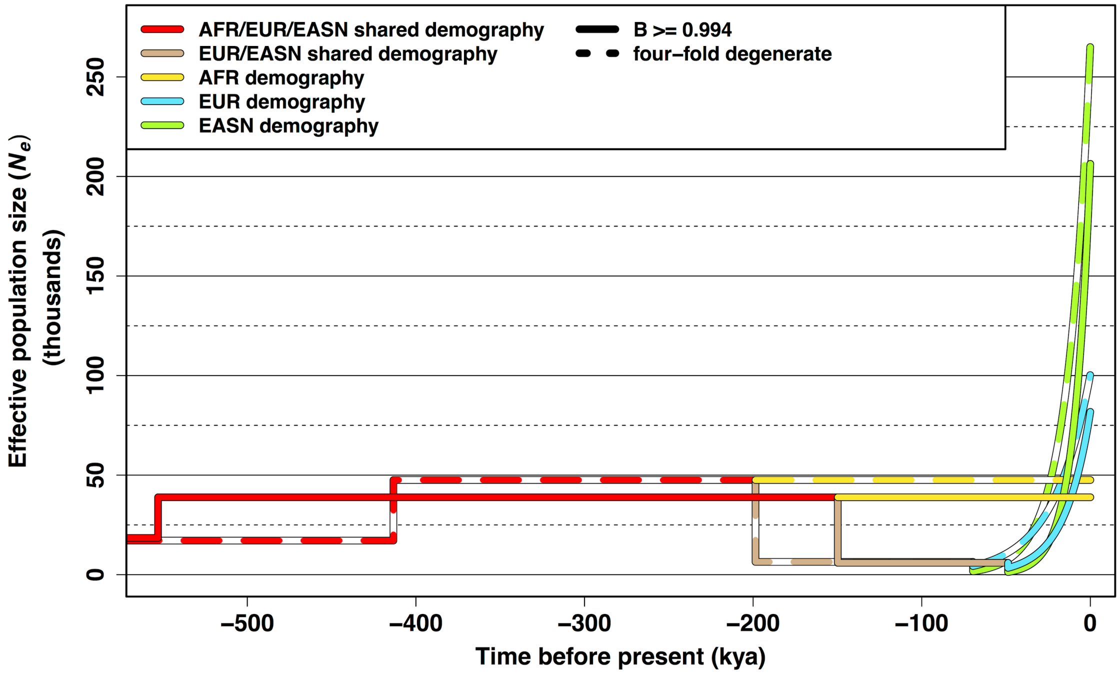

S1 Fig. Inference models inferred from TGP Complete Genomics (CG) high *B* neutral regions and coding four-fold degenerate sites.
Solid lines are the inference results from running dadi on 53 YRI (African), 64 CEU (European), and 62 CHS (East Asian) TGP CG samples (projected down to 106 chromosomes during inference procedure) across neutral regions in the highest 1% *B* bin ($B \geq 0.994$). Broken lines represent the inference results using the same CG samples but with sequence data only from coding four-fold degenerate synonymous sites. See Table A in S1 Text for parameter values.

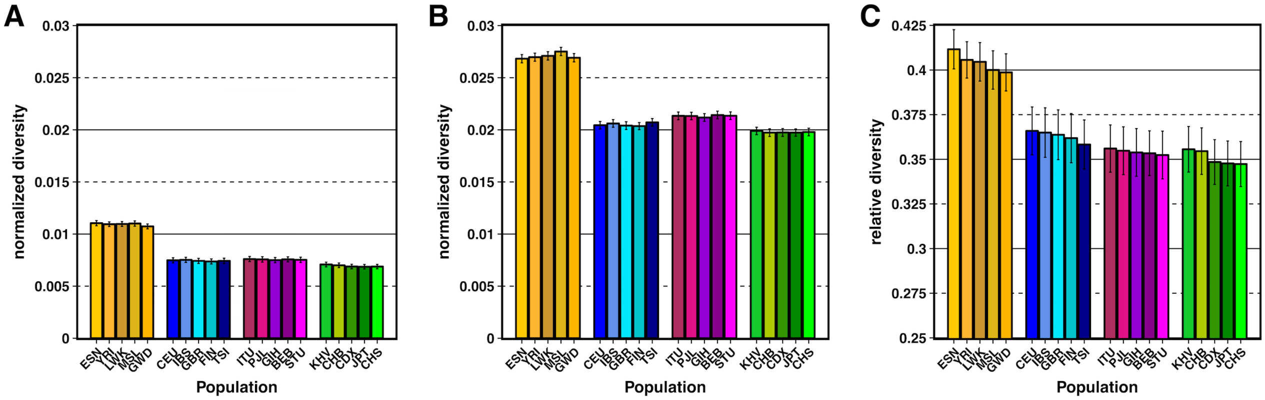

S2 Fig. Diversity for TGP non-admixed populations while controlling for GC-biased gene conversion and recombination hotspots.

(A) Normalized diversity (π/divergence) measured across the lowest 1% *B* quantile bin (strong BGS). (B) Normalized diversity measured across the highest 1% *B* quantile bin (weak BGS). (C) Relative diversity: the ratio of normalized diversity for the lowest 1% *B* bin to normalized diversity for the highest 1% *B* bin (π/π_{min}). Error bars represent ±1 SEM calculated from 1,000 bootstrapped datasets. See S2 Table for underlying data.

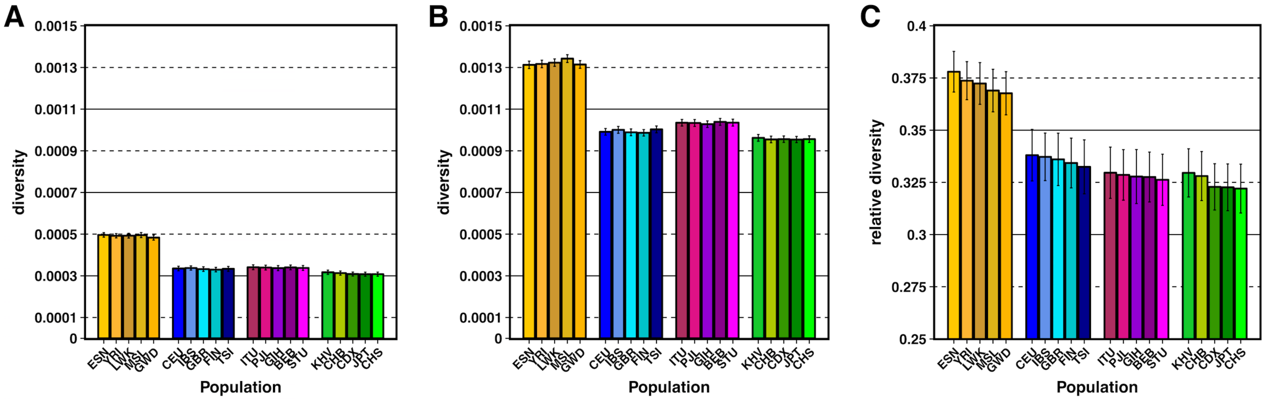

S3 Fig. Diversity for TGP non-admixed populations without normalizing by divergence with Rhesus macaque.

(A) Diversity (π) measured across the lowest 1% *B* quantile bin (strong BGS). (B) Diversity measured across the highest 1% *B* quantile bin (weak BGS). (C) Relative diversity: the ratio of diversity for the lowest 1% *B* bin to diversity for the highest 1% *B* bin (π/π_{min}). Error bars represent ±1 SEM calculated from 1,000 bootstrapped datasets. See S1 Table for underlying data.

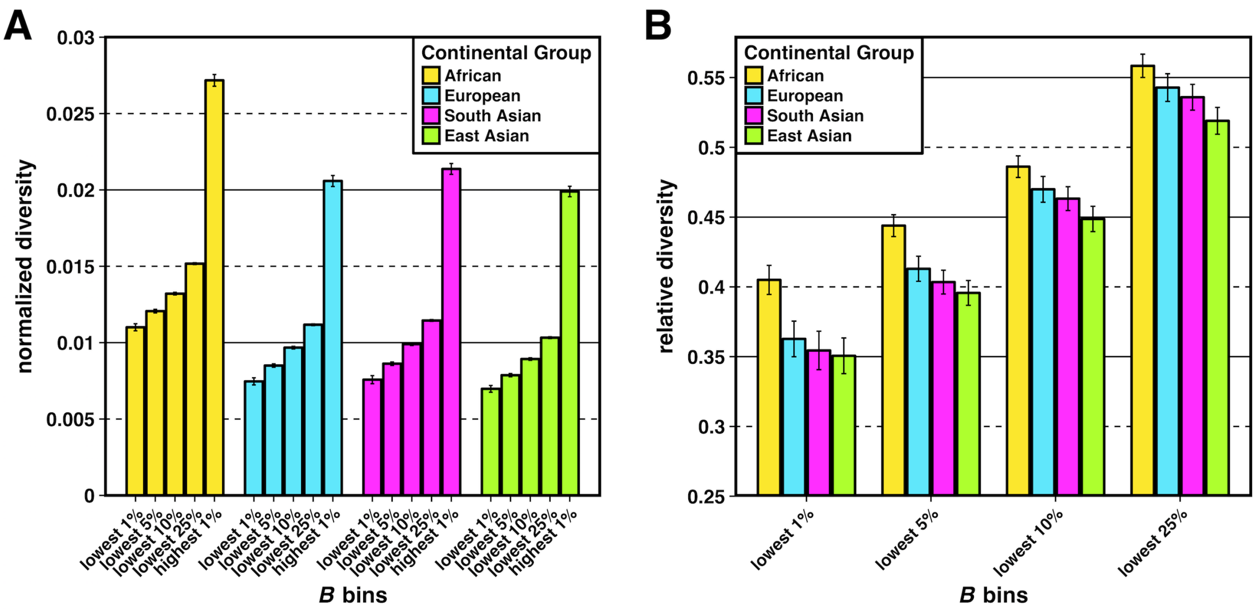

S4 Fig. Diversity for TGP continental groups while controlling for GC-biased gene conversion and recombination hotspots.

(A) Normalized diversity (π/divergence) measured across the lowest 1%, 5%, 10% and 25% *B* quantile bins (strong BGS) and the highest 1% *B* quantile bin (weak BGS). (B) Relative diversity (π/π_{min}) for the lowest 1%, 5%, 10%, and 25% *B* bins. Error bars represent ±1 SEM calculated from 1,000 bootstrapped datasets. See S2 Table for underlying data.

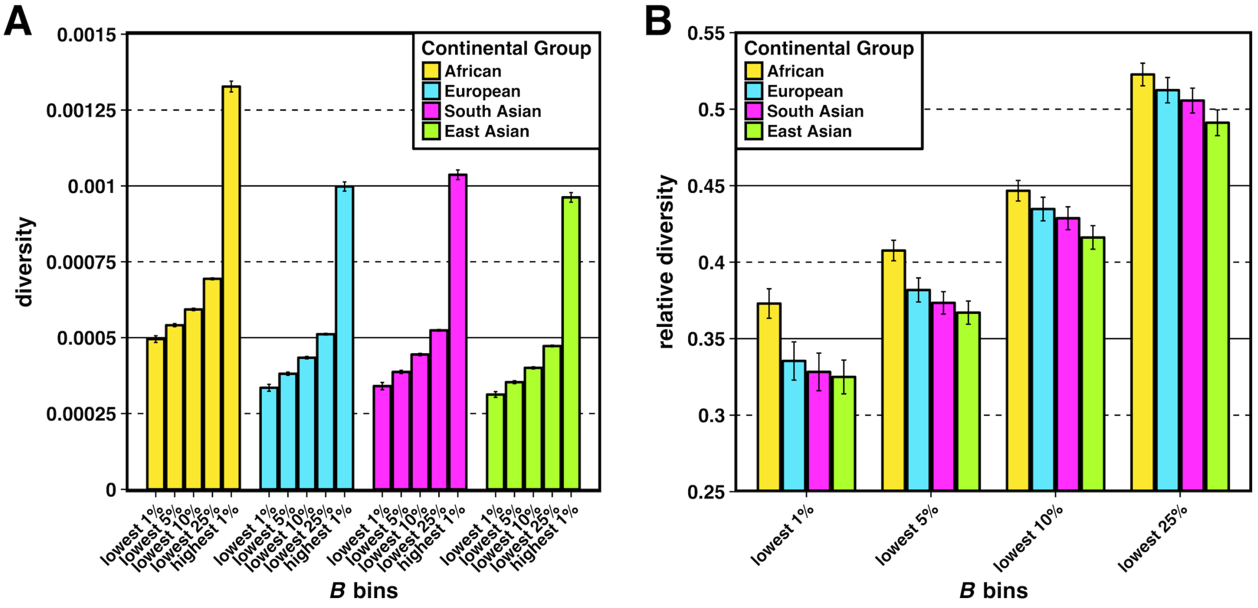

S5 Fig. Diversity for TGP continental groups without normalizing by divergence with Rhesus macaque.

(A) Diversity (π) measured across the lowest 1%, 5%, 10% and 25% *B* quantile bins (strong BGS) and the highest 1% *B* quantile bin (weak BGS). (B) Relative diversity (π/π_{min}) for the lowest 1%, 5%, 10%, and 25% *B* bins. Error bars represent ±1 SEM calculated from 1,000 bootstrapped datasets. See S1 Table for underlying data.

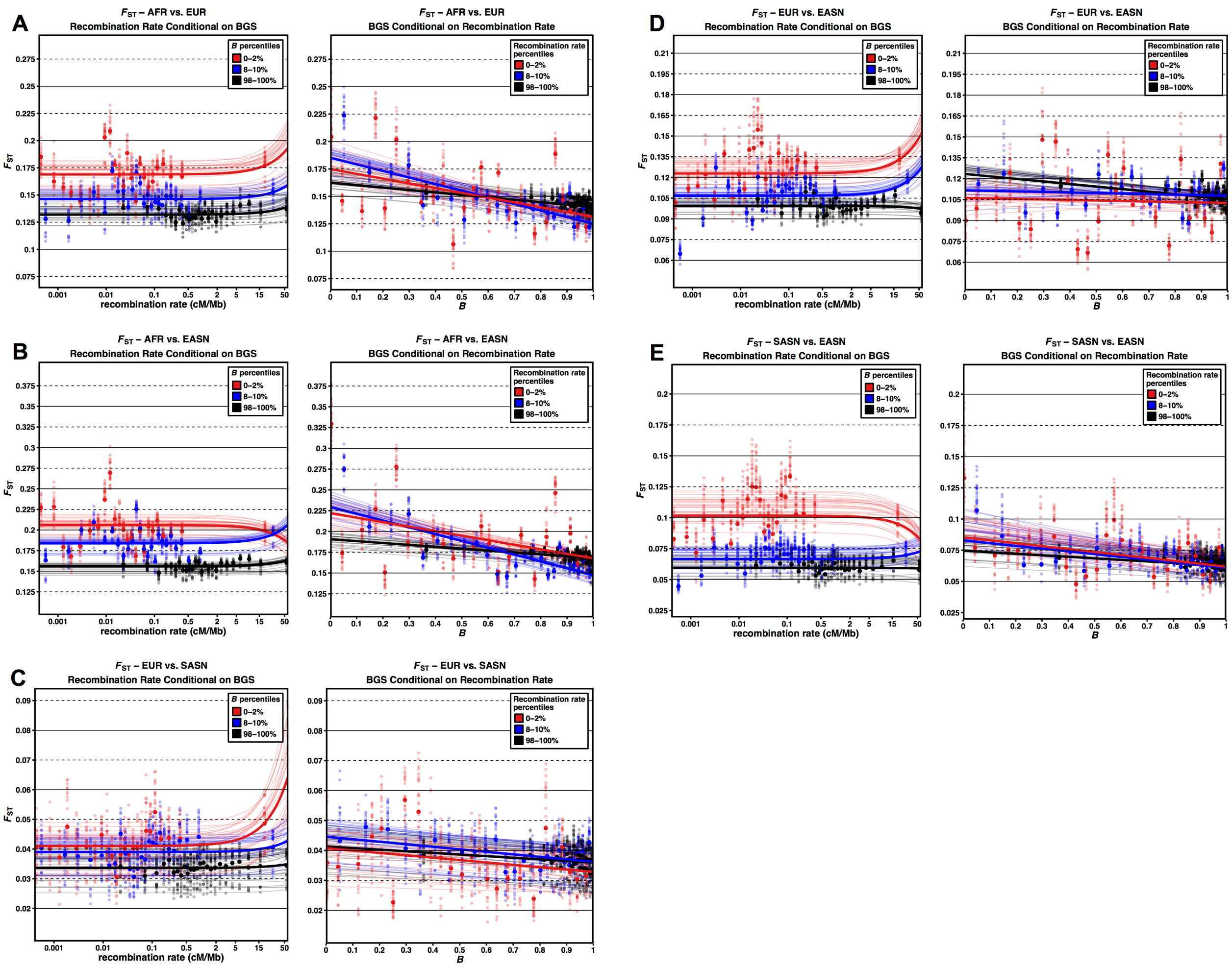

S6 Fig. F_{ST} measured across joint bins of *B* and recombination rate for different TGP continental groups.

The left panels of S6 Figs A-E show F_{ST} measured as a function of 25 4% recombination rate quantile bins conditional on three 2% *B* quantile bins (note log scale of x-axis for recombination rate). The right panels of S6 Figs A-E show F_{ST} measured as a function of 25 4% *B* quantile bins conditional on three 2% recombination rate quantile bins. The following continental group comparisons are shown for each plot: (A) African vs. European, (B) African vs. East Asian, (C) European vs. South Asian, (D) European vs. East Asian, (E) South Asian vs. East Asian. Smaller transparent points and lines show the F_{ST} estimates and corresponding lines of best fit (using linear regression) for each of the pairwise population comparisons within a particular pair of continental groups (25 comparisons total). Larger opaque points are mean F_{ST} estimates across all pairwise comparisons within a particular pair of continental groups (bold lines showing their corresponding lines of best fit).

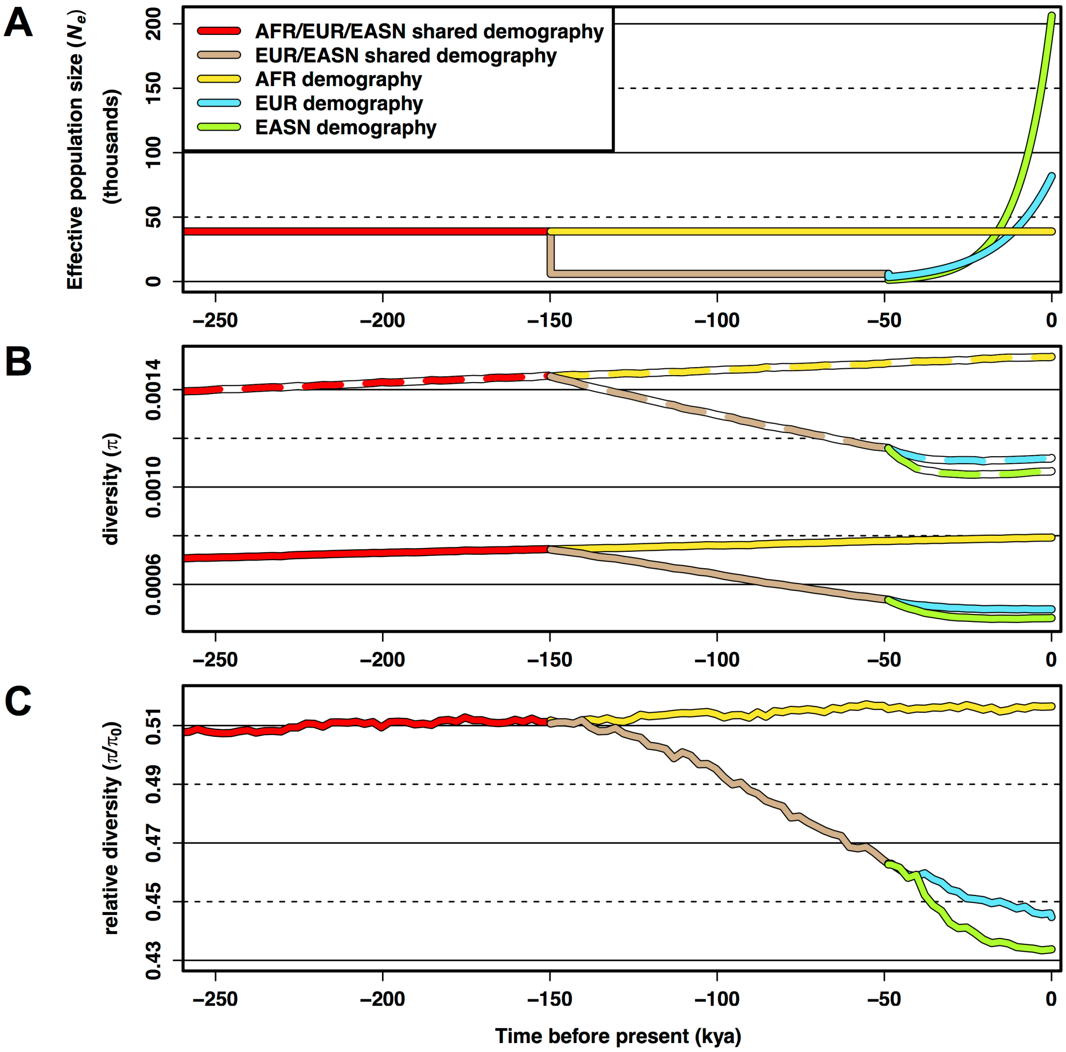

S7 Fig. Simulations of diversity and relative diversity under BGS using a human demographic model without migration.

(A) Inferred demographic model from Complete Genomics TGP data. The demographic model used for the simulations in S7 Fig are identical to those used for Fig 5, except that migration parameters between all populations are set to 0. (B) Simulated diversity at neutral sites across populations as a function of time under our inferred demographic model without BGS (π_0 - dashed colored lines) and with BGS (π - solid colored lines). (C) Relative diversity (π/π_0) measured by taking the ratio of diversity with BGS (π) to diversity without BGS (π_0) at each time point. Note that the x-axes in all three figures are on the same scale. Time is scaled using a human generation time of 25 years per generation. Simulation data was sampled every 100 generations (see S5 Table for exact values of mean π).

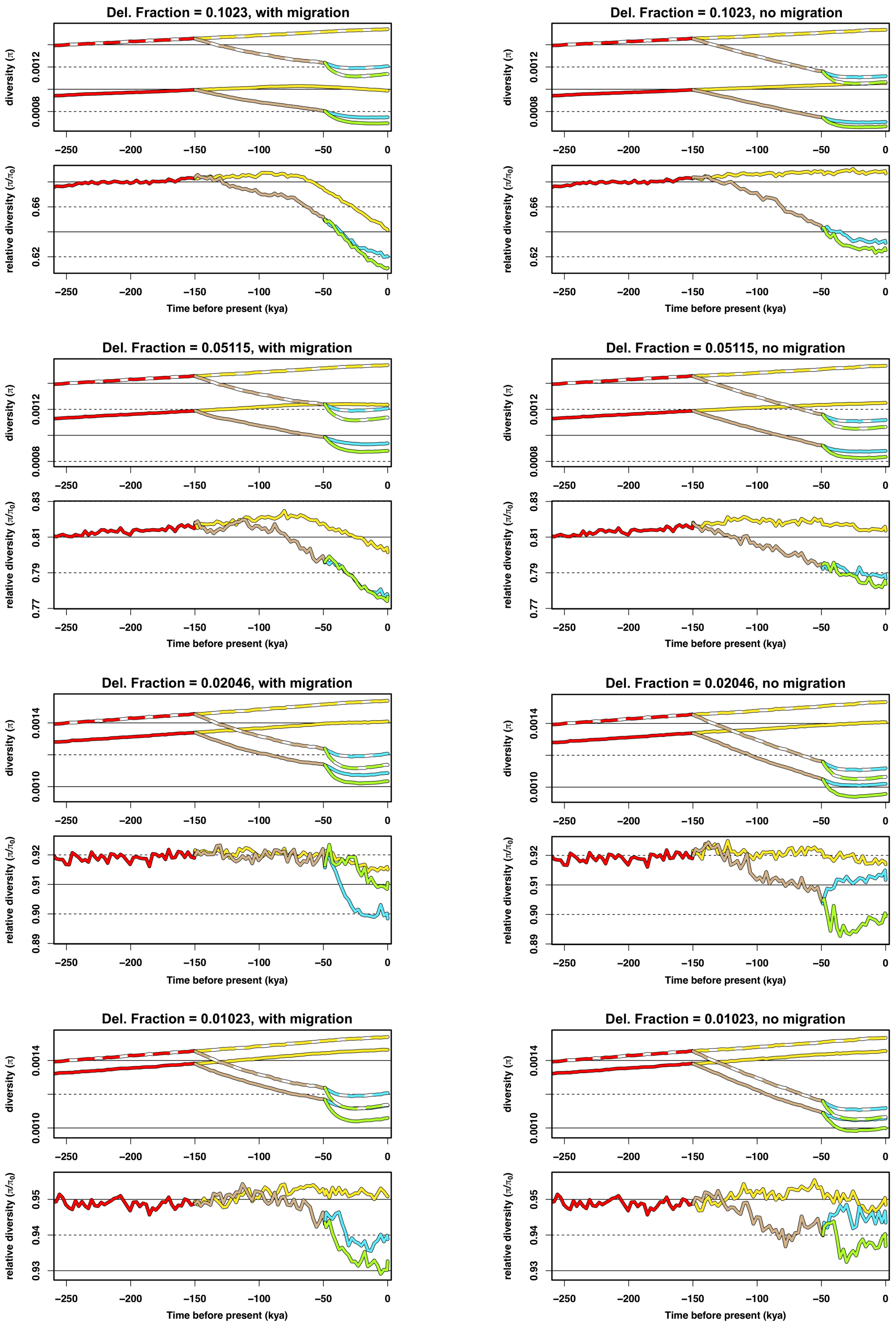
Del. Fraction = 0.1023, with migration
Del. Fraction = 0.1023, no migration
Del. Fraction = 0.05115, with migration
Del. Fraction = 0.05115, no migration
Del. Fraction = 0.02046, with migration
Del. Fraction = 0.02046, no migration
Del. Fraction = 0.01023, with migration
Del. Fraction = 0.01023, no migration
diversity (π)
relative diversity (π/π0)
Time before present (kya)
−250
−200
−150
−100
−50
0

S8 Fig. Simulations of diversity and relative diversity under BGS using various fractions of sites experiencing deleterious mutation.

Values for the deleterious site fraction are provided in the title for each set of plots. Left column plots show results of simulations under a demographic model with migration between all human populations. Right column plots show results of simulations under a demographic model with no migration. Colored lines represent different populations though time and are identical to those in Fig 5 and S7 Fig. The demographic model used is also identical to that in Fig 5 (for simulations with migration) and S7 Fig (for simulations without migration). Simulation data was sampled every 100 generations (see S5 Table for exact values of mean π).

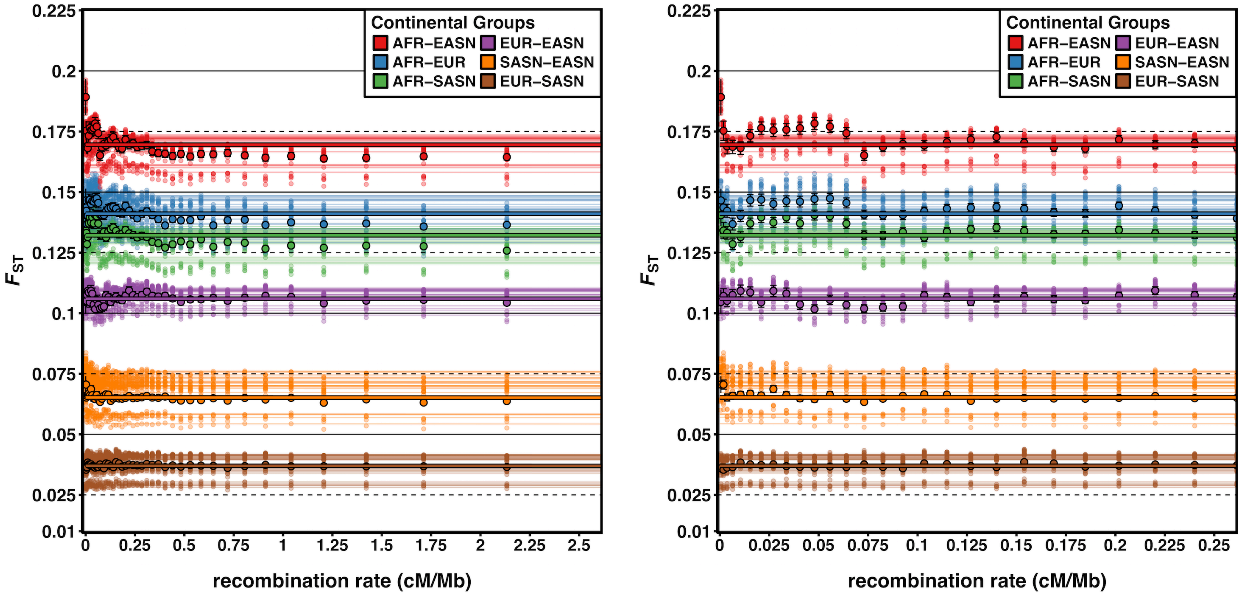

S9 Fig. F_{ST} is not correlated with recombination rate.

F_{ST} between TGP populations measured across 2% recombination rate quantile bins. The right panel of S9 Fig displays a narrower range of recombination rates to show detail. Smaller transparent points and lines show the estimates and corresponding lines of best fit (using linear regression) for F_{ST} between every pairwise population comparison within a particular pair of continental groups (25 pairwise comparisons each). Larger opaque points and lines are mean F_{ST} estimates and lines of best fit across all population comparisons within a particular pair of continental groups. Error bars represent ±1 SEM calculated from 1,000 bootstrapped datasets.

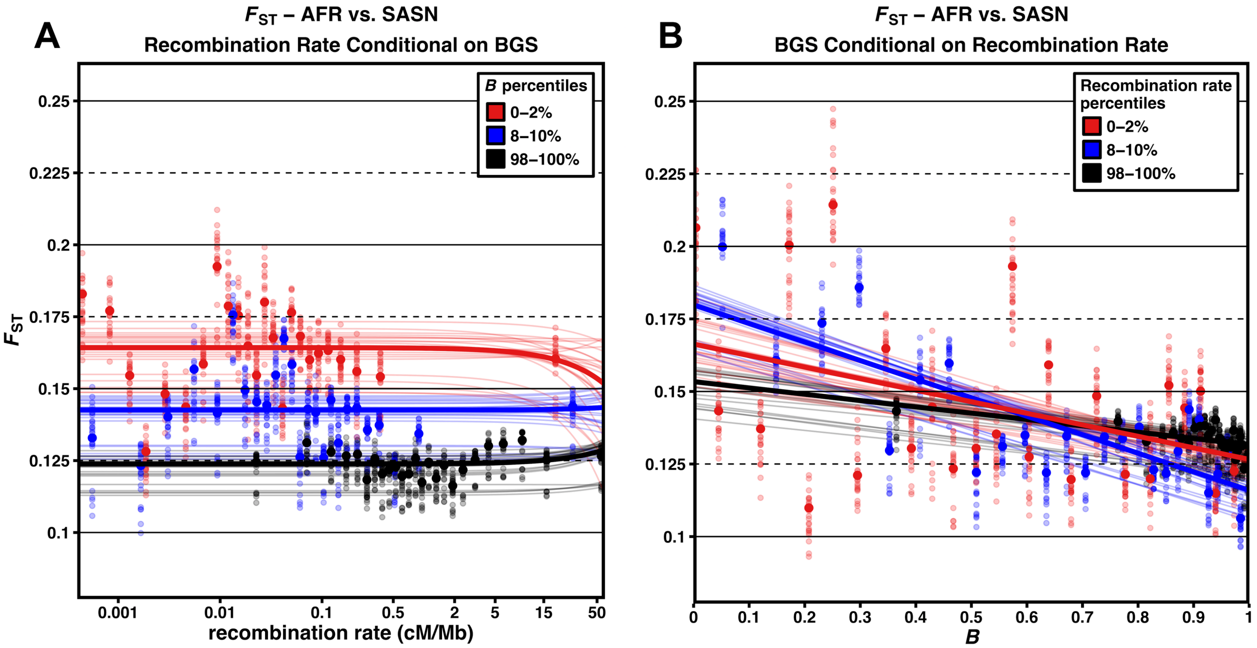

S10 Fig. F_{ST} between African (AFR) and South Asian (SASN) populations jointly across *B* and recombination rate.

(A) F_{ST} as a function of 25 recombination rate bins (4% quantile bins) conditional on three different 2% *B* quantile bins (note log scale of x-axis for recombination rate). (B) F_{ST} as a function of 25 *B* bins (4% quantile bins) conditional on three different 2% recombination rate quantile bins. Smaller transparent points and lines show the F_{ST} estimates and corresponding lines of best fit (using linear regression) for each of the pairwise comparisons of AFR vs. SASN Thousand Genomes Project (TGP) populations (25 comparisons total). Larger opaque points are mean F_{ST} estimates across all pairwise comparisons of AFR vs. SASN TGP populations (with bold lines showing their corresponding lines of best fit).

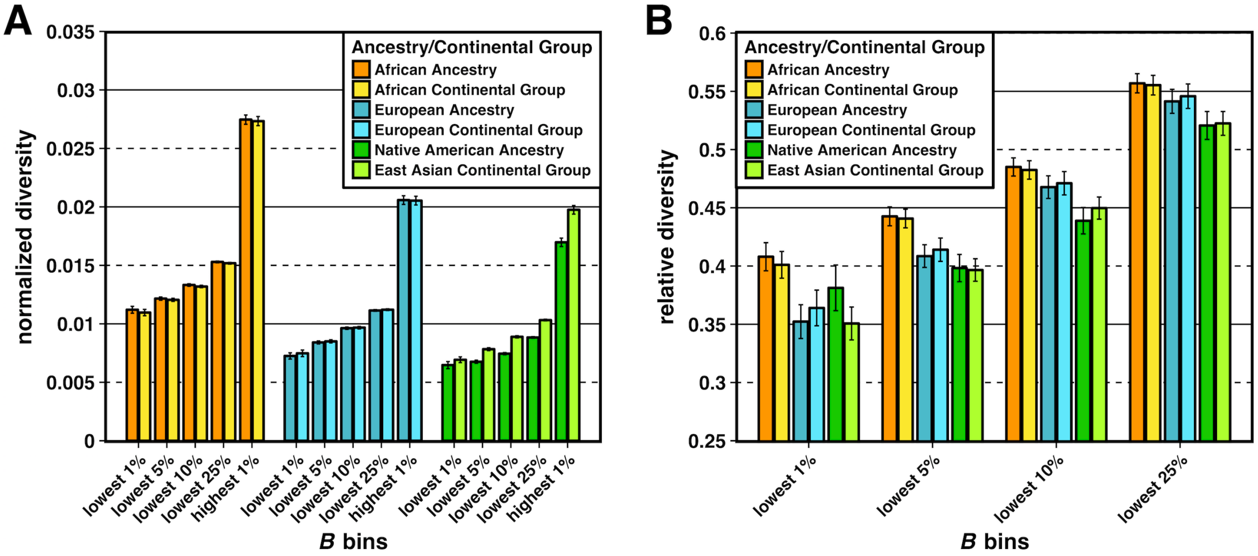

S11 Fig. Comparing patterns of diversity between local ancestry segments of admixed samples and continental groups.

(A) Normalized diversity (heterozygosity/divergence) and (B) Relative diversity: the ratio of normalized diversity in the lowest *B* quantile bins (strong BGS) in (A) to normalized diversity in the highest 1% *B* quantile bin (weak BGS) in (A). Local ancestry segments include African, European, and Native American ancestries. Continental groups include African, European, and East Asian. Error bars represent ±1 SEM calculated from 1,000 bootstrapped datasets. See S4 Table for underlying data.

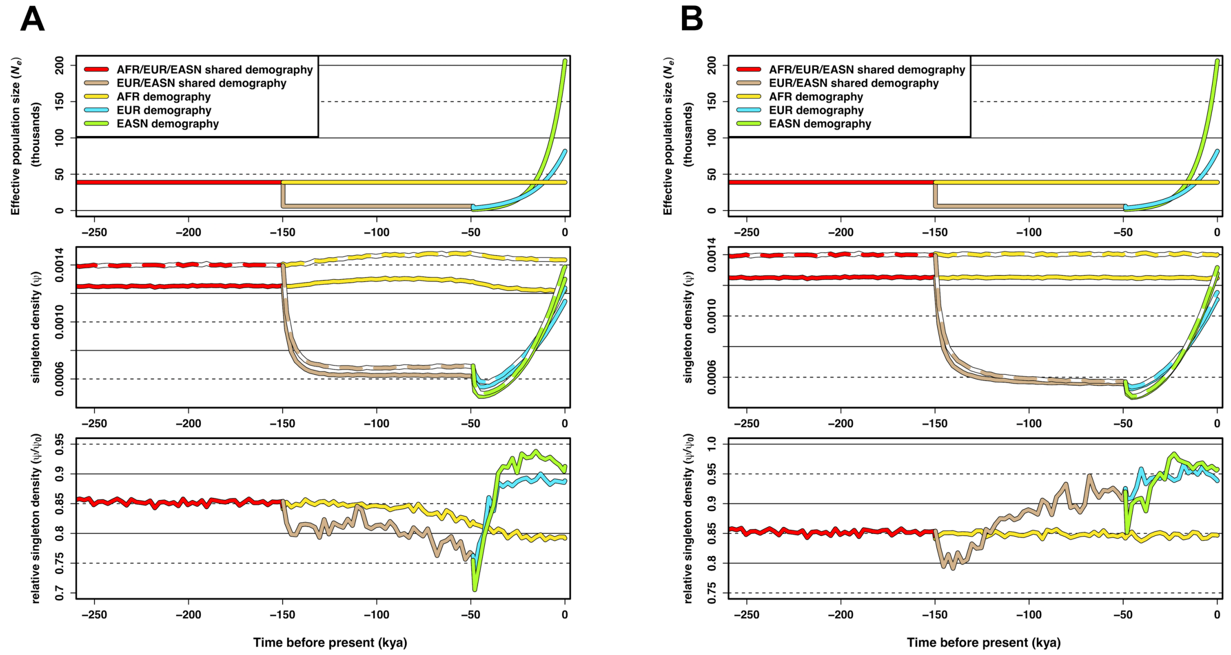

S12 Fig. Simulations of singleton density and relative singleton density.
(A) Results of simulations under a demographic model with migration between all human populations. (B) Results of simulations under a demographic model with no migration. The second row of (A) and (B) shows measurements of singleton density (i.e., number of singletons observed per site) from simulations without BGS (ψ_0 - dashed colored lines) and with BGS (ψ - solid colored lines). The bottom row of (A) and (B) shows corresponding relative singleton density (ψ/ψ_0) measured by taking the ratio of singleton density with BGS (ψ) to singleton density without BGS (ψ_0) at each sampled generation time point. The simulation data used for these measurements is identical to that of Fig 5 (for simulations with migration) and S7 Fig (for simulations without migration). See S6 Table for exact values of mean ψ.

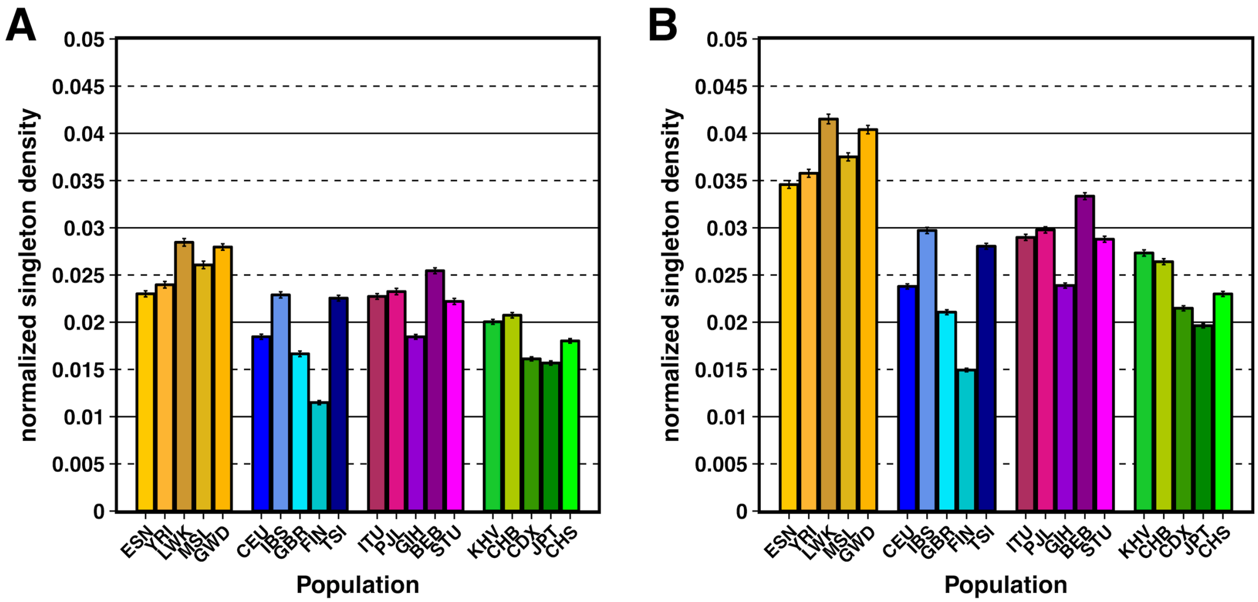

S13 Fig. Singleton density for the lowest and highest 1% *B* quantile bins for non-admixed populations of the Thousand Genomes Project (TGP).

(A) Normalized singleton density (ψ/divergence) measured across the lowest 1% *B* quantile bin (strong BGS). (B) Normalized singleton density measured across the highest 1% *B* quantile bin (weak BGS). TGP population labels are indicated below each bar (see Table L in S1 Text for population label descriptions), with African populations colored by gold shades, European populations colored by blue shades, South Asian populations colored by violet shades, and East Asian populations colored by green shades. Error bars represent ±1 SEM calculated from 1,000 bootstrapped datasets. See S3 Table for underlying data.
